# Healthcare-associated infections caused by chlorhexidine-tolerant *Serratia marcescens* carrying a promiscuous IncHI2 multi-drug resistance plasmid in a veterinary hospital

**DOI:** 10.1371/journal.pone.0264848

**Published:** 2022-03-17

**Authors:** Joanne L. Allen, Nicholas P. Doidge, Rhys N. Bushell, Glenn F. Browning, Marc S. Marenda

**Affiliations:** 1 Asia-Pacific Centre for Animal Health, Faculty of Veterinary and Agricultural Sciences, The University of Melbourne, Parkville, Victoria, Australia; 2 Asia-Pacific Centre for Animal Health, Faculty of Veterinary and Agricultural Sciences, The University of Melbourne, Werribee, Victoria, Australia; Tianjin University, CHINA

## Abstract

The bacterium *Serratia marcescens* can cause opportunistic infections in humans and in animals. In veterinary settings, the diversity, reservoirs and modes of transmission of this pathogen are poorly understood. The phenotypes and genotypes of *Serratia* spp. isolated from dogs, cats, horses, a bird and a rabbit examined at an Australian veterinary hospital between 2008 and 2019 were characterised. The isolates were identified as *S*. *marcescens* (n = 15) or *S*. *ureilytica* (n = 3) and were placed into four distinct phylogenetic groups. Nine quasi-clonal isolates associated with post-surgical complications in different patients displayed high levels of resistance to the antimicrobials fluoroquinolones, cephalosporins, aminoglycosides, and to the disinfectant chlorhexidine. A *Serratia* sp. with a similar resistance profile was also isolated from chlorhexidine solutions used across the Hospital, suggesting that these infections had a nosocomial origin. A genomic island encoding a homolog of the *Pseudomonas* MexCD-OprJ biocide efflux system was detected in the chlorhexidine-tolerant *Serratia*. The nine multi-drug resistant *Serratia* isolates also possessed a Ser-83-Ile mutation in GyrA conferring fluoroquinolone resistance, and carried a large IncHI2 conjugative plasmid encoding antimicrobial and heavy metal resistances. This replicon was highly similar to a plasmid previously detected in a strain of *Enterobacter hormaechei* recovered from the Hospital environment. IncHI2 plasmids are commonly found in *Enterobacteriaceae*, but are rarely present in *Serratia* spp., suggesting that this plasmid was acquired from another organism. A chlorhexidine-tolerant *Serratia* isolate which lacked the IncHI2 plasmid was used in mating experiments to demonstrate the transfer of multi-drug resistance from a *E*. *hormaechei* donor. This study illustrates the importance of environmental surveillance of biocide-resistance in veterinary hospitals.

## Introduction

*Serratia marcescens* is a Gram negative bacterium belonging to the *Yersiniaceae* family in the order *Enterobacterales*. It is found in the environment (including soil, water, sludge and compost), plants, insects, and clinical specimens from humans and animals. In humans, it is a cause of opportunistic and nosocomial infections, with increasing concerns for neonatal units of hospital [1]. *S*. *marcescens* is able to survive in harsh conditions, including in the biocide chlorhexidine [2]. In health care settings, solutions of chlorhexidine gluconate are commonly used as a skin disinfectant, and can be contaminated by environmental bacteria such as *Pseudomonas*, *Burkholderia*, *Ralstonia*, *Achromobacter*, and *Serratia* spp. [3]. Outbreaks of *S*. *marcescens* infections have been attributed to chlorhexidine solutions in human hospitals [4,5], and more recently in veterinary hospitals [6].

Although *S*. *marcescens* are intrinsically or frequently resistant to antibiotics such as tetracyclines, ampicillin, amoxicillin-clavulanate, 1^st^ generation cephalosporins, and colistin, many clinical isolates are susceptible to trimethoprim/sulfamides, aminoglycosides, third generation cephalosporins, carbapenems and fluoroquinolones [7]. However, opportunistic infections caused by multi-drug resistant *S*. *marcescens* can restrict the range of acceptable therapeutic options in companion animals.

The phylogeny of the genus *Serratia* is complex, and strain diversity within the *S*. *marcescens* species is poorly understood. Biochemical phenotypic sub-typing methods have been developed [8], but these approaches are now superseded by DNA sequence typing [9], or full genome sequencing and phylogenetic analysis. Comparative genomic studies have explored the relatedness of *S*. *marcescens* strains isolated from humans, insects, and the environment [10] but very limited sequence data is available for isolates from domestic animals. In particular, the taxonomic classification, phylogenetic diversity and repertoires of antimicrobial or biocide resistances in these isolates is largely unknown, despite serious therapeutic and zoonotic consequences.

Transfer of antimicrobial resistance genes (ARGs) by mobile genetic elements such as conjugative plasmids can produce multi-drug resistant bacteria with the potential to cause intractable infections in immunocompromised patients. In human health settings, nosocomial infections have been associated with the colonisation of hospital sinks by multi-resistant organisms [11] and the exchange of genetic material between different bacterial species in these environments [12]. This is a particular concern with biocide-tolerant organisms that can persist in hospitals despite routine disinfection programs.

This problem was recently illustrated in the veterinary hospital of the University of Melbourne (U-Vet) by the detection of a large IncHI2 conjugative plasmid harboured by a multi-drug resistant *Enterobacter hormachei* which had contaminated a hand-wash basin [13]. This plasmid carried heavy metal resistance genes along with several ARGs of high concern, including an extended spectrum beta lactamase (ESBL) and a colistin resistance gene, associated with insertion sequences and transposons. A thorough disinfection protocol eventually eliminated the organism from the sink, and no nosocomial infection could be attributed to this organism amongst the hospital patients. However, this discovery highlighted the importance of actively monitoring transmissible antimicrobial resistances amongst opportunistic pathogens from hospital patients. Here, a collection of *Serratia* spp. isolated from U-Vet patients between 2008 and 2019 was examined to assess their taxonomic identification, genomic diversity, and ARGs. A quasi-clonal group of strains isolated from complicated surgical procedures, which could survive in chlorhexidine and carried a IncHI2 plasmid highly similar to the one harboured by the *E*. *hormachei* strain found in the hospital environment, was characterised.

## Materials and methods

### 1. Bacterial strains and culture conditions

Culture plates were obtained from Micromedia (Edwards). All strains were isolated from clinical specimens by plating on Columbia sheep blood agar (SBA) and MacConkey agar (MAC) followed by incubation at 37˚C overnight with 5% CO_2_. Presumptive identification of Gram negative rods, oxidase negative, non-lactose fermenters as *Serratia* spp. was performed with the API rapid 32E kit (BioMerieux). Pure cultures of isolates were stored at -80˚C using the Protect beads system (Thermo Fisher Scientific). Production of Extended Spectrum Beta Lactamase (ESBL) was tested by sub-culture on Brilliance-ESBL agar plates (Oxoid). Antimicrobial susceptibility testing was performed at the time of isolation by disc diffusion according to the Calibrated Dichotomous Susceptibility (CDS) method [14] and afterward on thawed stock cultures by broth microdilution using Sensititre COMPGN1F plates on an ArisX2 system (Thermo Fisher Scientific).

### 2. DNA sequencing and genome assembly

Illumina-platform sequencing of *S*. *marcescens* genomic DNAs was performed at the Australian Genome Research Facility, Melbourne, Australia. Reads were filtered for quality scores >20 and adapters were removed using Trim Galore v0.4.4 [15].

Nanopore sequencing was performed on genomic DNAs purified using the Wizard HWM genomic DNA prep kit (Promega). The libraries were prepared with the rapid barcoding kit SQK-RBK004 (Oxford Nanopore) and loaded on a MinION MK-Ib device fitted with flowcell R9.4. Live rapid basecalling was performed with MinKnow 20.13.3 using guppy version 4.2.2 (Oxford Nanopore), followed by filtering of reads with quality >7, demultiplexing and barcode removal with guppy_barcoder (Oxford Nanopore). Reads of more than 1 kb were processed with Filtlong [16] using their corresponding Illumina datasets as reference sequences.

Long reads only assemblies were performed with Flye 2.8.1 [17]. All other assemblies were performed with Unicycler v0.4.7 [18]. For strains CM2012_028, CM2015_854, CM2016_324, CM2017_569, and CM2019_254, hybrid genome assemblies were performed using the Illumina and Nanopore sequence reads as input datasets, and the Flye output fasta file as a guide, with the parameter “—existing_long_read_assembly”. For all other strains, Illumina reads only were used for assemblies. Following assembly, the resulting fasta sequence files were annotated with Prokka version 1.14.6 [19].

### 3. Comparative genome analysis and phylogeny

All-against-all genome average nucleotide identity was calculated with FastANI [20] and the resulting matrix of percentage identities was visualised in R (https://www.r-project.org/) using the package heatmap3 [21], with the “dist” function and the method “complete” for hierarchical clustering.

Core Single Nucleotide Polymorphism (SNP) analysis was performed on a curated set of *Serratia* spp. genomes with the Harvest suite of programs [22] using the -x parameter for filtering out SNPs potentially present in regions of recombination. Individual SNP analysis of selected genomes was performed with snippy [23].

For phylogenetic analyses, the genes *adk*, *aro*C, *aro*K, *dna*A, *dna*J, *dna*K, *gyr*B, *par*E, *pur*A, *rec*R, *rho*, *rpo*B and *rpo*H were extracted from publicly available complete genomes of *Serratia* spp. and compiled into multi-FASTA files. Genomes that did not contain a full set of query sequences were discarded from the analysis. Multiple alignments were constructed separately for each multi-FASTA file with MUSCLE [24], then concatenated into a single alignment file and analysed with MEGA [25] to infer a consensus tree by using the Maximum Likelihood method and General Time Reversible model with 100 bootstrap replicates. Trees were visualised and annotated with iTOL [26].

### 4. Genome contents analysis

Antimicrobial resistance genes and plasmids were predicted with ABRicate using the database resfinder and plasmidfinder, respectively [27]. The importance rating of antimicrobials for which a resistance was predicted was reported using the ASTAG classification [28]. Plasmid similarity search was performed on the online Plasmid Database (PLSDB) server [29] with the “mash_dist” strategy.

Sequence alignments were performed and visualised with BRIG [30], Progressive Mauve [31], and Clinker [32]. Prophages were identified with the online tool, PHASTER [33]. Genomic islands were explored with the online tool IslandViewer 4 [34]. Integrative Conjugative Elements (ICEs) were detected with the online tool ICEFinder [35]. Pan-genome analysis was performed with the Roary pipeline [36] with default presets (minimum identity for blastp 95%, core genes possessed by at least 99% isolates). Predicted protein sequences were analysed with the online tool KofamKOALA [37] from the Kyoto Encyclopedia of Genes and Genomes [38]. A set of 753 experimentally confirmed biocide resistance genes downloaded from the online database BacMet [39] to produce a Blast database which was used to search all predicted gene products encoded by *Serratia* spp. genomes, using BlastP version 2.9.0+.

### 5. Chlorhexidine resistance assays

To mimic the disinfection protocols used in the U-Vet, the effect of a transient exposure to chlorhexidine was assessed as follows. A loopful of pure culture grown overnight at 37°C on agar plates was mixed thoroughly in 0.85% sterile saline (2.5 mL). Uninoculated 0.85% sterile saline was used as negative control. After adjustment of the OD_600_ to 0.5, each suspension (100 μL) was placed into 2 microfuge tubes. Sterile water or a 1% Chlorexidine-gluconate solution (100 μL), routinely used in the Hospital, was added to the bacteria and mixed by inversion. After incubation at room temperature for 5 minutes, the disinfectant was neutralised by adding 800 μL Letheen Broth (Thermo Fisher Scientific) to the treated suspensions. Aliquots (200 μL) were transferred in duplicate into sterile flat-bottom 96 well plates (Nunc) and incubated at 37°C. The plate ODs were read at regular intervals on a MultiSkan plate reader (Thermo Fisher Scientific) using a 620 nm filter. Individual growth data were analysed with the R package ‘growthcurver’ to compare the separate plots [40]. The approximate generation time during the logarithmic phase was estimated by dividing the time interval (in minutes) of 2 timepoints between the first and the second hour of incubation by the difference between the log2 of the OD values at these timepoints.

To evaluate the Minimum Inhibitory Concentration (MIC) and Minimum Bactericidal Concentration (MBC) of chlorhexidine, a 0.5% solution of the disinfectant was serially diluted with a two-fold ratio in Cation Adjusted Muller-Hinton Broth (Thermo Fisher Scientific) and each dilution (100 μL) was dispensed into 96 well plates. To prepare the inoculum, bacterial suspensions were prepared from fresh agar plate cultures using sterile demineralised water and adjusted to 0.5 Mc Farland with a calibrated nephelometer (Thermo Fisher Scientific). Each suspension (15 μL) was added to Cation Adjusted Muller-Hinton Broth (1.5 mL), and this inoculum (100 μL) was mixed with the serially diluted chlorhexidine. The plates were incubated at 37°C overnight, the turbidity of the medium was assessed visually. An aliquot from each well (10 μL) was spotted on MacConkey plates and incubated at 37°C overnight.

### 6. Mating experiments

*Enterobacter hormaechei* strain CM2018_216 (donor) and *S*. *marcescens* strain CM2017_569 (recipient) were grown at 37°C for 18 hours on Columbia sheep blood agar. A 1 μL sterile plastic loop was used to harvest 3–5 colonies from each plate and resuspended in 0.85% saline solution (2.5 mL). For mating experiments, a 1:10 mixture of CM2018_216 (5 μL) and of CM2017_569 (50 μL) was cultured on a nutrient agar plate. As controls, each suspension (5 μL) was used to inoculate 2 separate nutrient agar plates. After 24 hours of incubation at 25°C, 3 loopfuls of culture were harvested with a 1 μL sterile plastic loop and transferred in a sterile microfuge tube containing of 0.85% saline solution (50 μL) to form a dense suspension. To inactivate the donor strain, chlorhexidine solution (1%, 50 μL) was added to the suspension. After 15 minutes of incubation at room temperature, Letheen Broth (400 μL) was added to the mixture and the total contents of the tube were spread on nutrient agar. Once the plates had dried, paper discs containing 10 ug of cefpodoxime or 10 ug of ceftazidime were placed on the centre of the plate to select for ESBL producers. Following 18 hours incubation at 37°C, satellite colonies growing in the zone of inhibition around the discs were selected and purified on SBA. The putative transconjugants were confirmed as *S*. *marcescens* with the rapid API 32E kit. The ESBL phenotype of the transconjugants was assessed by double disk diffusion synergy test [41].

### 7. Sequence accession numbers

The sequences from the 18 *Serratia* isolates were deposited in the NCBI database under the BioProject accession number PRJNA784350, with the following BioSample accession numbers: SAMN23485795, SAMN23485796, SAMN23485797, SAMN23485798, SAMN23485799, SAMN23485800, SAMN23485801, SAMN23485802, SAMN23485803, SAMN23485804, SAMN23485805, SAMN23485806, SAMN23485807, SAMN23485808, SAMN23485809, SAMN23485810, SAMN23485811 and SAMN23485812. The nucleotide accession numbers are: CP091120 (CM2019_254), CP091121 (CM2016_324), CP091122 (CM2012_028), CP091123 and CP091124 to CP091130 (CM2017_569), CP091125 (CM2015_854).

## Results

### 1. A group of Serratia spp. isolated from various hospitalised animals are resistant to high importance antimicrobials

Over the period 2008–2020, the clinical microbiology laboratory of the Veterinary Clinic and Hospital (U-Vet) at the University of Melbourne carried out 4536 bacterial identifications from animal patients. Isolation of a *Serratia* spp. was reported in 30 (0.7%) cases. The cultures were obtained from unrelated animals (11 dogs, 9 cats, 6 horses, 1 rabbit and 1 bird) except for 3 isolates which were collected a few weeks apart from the same cat (Table 1). The infections appeared to be sporadic, with 0 to 4 isolates reported in any year.

**Table 1:** Clinical history and key phenotypic characters of *Serratia* spp. isolates.

Accession	Submission	Host	History^1^	Specimen	API biocode	Red^2^	SF^3^	W^4^	Enr^5^	ESBL^6^	Seq.^7^
CM2008_163	14/03/2008	Avian #1	Necrotic hepatitis budgerigar	Liver swab, post mortem	6 3 5 1 0 3 6 6 6 0 1	-	S	S	S	-	I.
CM2012_028	16/01/2012	Feline #1	Urinary Tract Infection	Urine, cystocentesis	6 2 5 1 0 3 6 6 6 0 1	+	S	S	S	-	I./N.
CM2012_118	17/02/2012	Feline #1	Urinary Tract Infection	Urine, cystocentesis	ND	+	S	S	S	-	I.
CM2012_298	1/05/2012	Equine #1	Serous fluid in ear	Guttural pouch biopsy	6 3 5 1 0 3 6 6 6 0 1	-	S	R	S	-	I.
CM2012_306	4/05/2012	Feline #1	Urinary Tract Infection	Urine, cystocentesis	ND	+	S	S	S	-	I.
CM2014_932	12/12/2014	Equine #2	Fetlock arthroscopy (S)	Synovial fluid	6 2 5 1 0 1 6 0 2 0 1	-	R	R	R	+	I.
CM2015_078	18/02/2015	Feline #2	Tibial fracture repair (S)	Screws	6 2 5 1 0 3 6 2 2 0 1	-	R	R	R	+	I.
CM2015_137	12/03/2015	Canine #1	Endotracheal ventilation (ICU)	Tracheal wash	6 2 5 1 0 1 6 2 2 0 1	-	R	R	R	+	I.
CM2015_244	13/04/2015	Feline #3	Wound debridement and tail amputation (S)	Wound swab	6 2 5 0 0 1 6 0 2 0 1	-	R	R	R	+	I.
CM2015_854	28/09/2015	Canine #2	Amputation for osteosarcoma (S)	Wound fluid	6 2 5 1 0 1 6 2 2 0 1	-	R	R	R	+	I./N.
CM2016_091	10/02/2016	Canine #3	Pyrexia after cruciate surgery (S)	Blood	6 2 5 1 0 1 6 2 2 0 0	-	R	S	R	+	I.
CM2016_261	12/04/2016	Feline #4	Herniated bladder (S)	Abdominal and right flank swab	6 2 5 1 0 3 6 2 2 0 1	-	R	R	R	+	I.
CM2016_324	28/04/2016	Feline #5	Cystic Kidney Disease and pyelonephritis	Free catch urine	6 3 5 1 0 3 6 6 6 0 1	-	S	S	S	-	I./N.
CM2016_384	16/05/2016	Canine #4	Exploratory laparotomy (S)	Peritoneal fluid	6 2 4 1 0 1 6 2 2 0 1	-	R	R	R	+	I.
CM2017_569	23/06/2017	Canine #5	Urothelial cell carcinoma	Free catch urine	6 3 5 1 0 3 6 6 6 0 1	-	R	S	S	-	I./N.
CM2017_728	7/08/2017	Equine #3	Prosthetic laryngoplasty (S)	Swab from infected site	6 2 5 1 0 1 6 2 2 0 1	-	R	R	R	+	I.
CM2019_254	11/03/2019	Equine #4	Suspect strangles	Guttural pouch biopsy	6 2 5 0 0 3 6 6 6 2 1	-	S	S	S	-	I./N.
CM2019_352	4/04/2019	Rabbit #1	Chronic dacrocystitis	Nasal flush and tearduct swab	6 3 5 1 0 1 6 6 6 2 1	-	S	S	S	-	I.

1: History: “S” denotes Post-surgery complications.

2: Red: Presence of red pigment.

3: SF: Sulfafurazole Resistance (R) or susceptibility (S).

4:W: Trimethoprim Resistance (R) or susceptibility (S).

5: Enr: Enrofloxacin Resistance (R) or susceptibility (S).

6: ESBL: Presence of an Extended Spectrum Beta-Lactamase.

7: Seq: Whole Genome Sequencing and Assembly: I., Illumina only, I./N. Illumina/Nanopore.

The retrospective examination of antimicrobial susceptibility reports of a set of 18 *Serratia* spp. isolates kept in collection (Table 1) revealed two main resistance patterns. Nine isolates were resistant to sulfafurazole/trimethoprim, enrofloxacin, and 3^rd^ generation cephalosporins. Moreover, they grew on Extended Spectrum Beta Lactamase (ESBL) detection agar plates. These 9 U-Vet multi-drug resistant isolates (hereafter referred to as MDR) were overwhelmingly associated with post-surgical complications in diverse hospitalised patients, whereas no commonality in the circumstances of infections was evident in the other isolates, which were susceptible to the above mentioned drug panel, and did not grow on ESBL plates.

Sensititre broth microdilution results confirmed that these MDR isolates had higher Minimum Inhibitory Concentration (MIC) values with 3^rd^ generation cephalosporins and fluoroquinolones, as well as with gentamicin and trimethoprim-sulfamide combination (Table 2).

**Table 2:** Minimum inhibitory concentration of *Serratia* spp. isolates from the U-Vet.

Antimicrobic	Range (ug/mL)	CM2014_932	CM2015_078	CM2015_137	CM2015_854	CM2016_384	CM2017_728	CM2017_569	CM2008_163	CM2016_324	CM2019_254	CM2019_352	CM2012_028
Ampicillin	0.25–8	> 8	> 8	> 8	> 8	> 8	> 8	> 8	> 8	> 8	> 8	= 8	> 8
Amoxicillin/ Clavulanic_Acid	0.25/0.12-8/4	= 8	> 8	> 8	> 8	> 8	> 8	> 8	> 8	> 8	> 8	> 8	> 8
Cefalexin	0.5–16	> 16	> 16	> 16	> 16	> 16	> 16	> 16	> 16	> 16	> 16	> 16	> 16
Cefazolin	1–32	> 32	> 32	> 32	> 32	> 32	> 32	> 32	> 32	> 32	> 32	> 32	> 32
Cefovecin	0.25–8	> 8	> 8	> 8	> 8	> 8	= 8	= 4	= 2	= 1	= 2	= 0.5	= 2
Cefpodoxime	1–8	> 8	> 8	> 8	> 8	> 8	= 8	= 2	= 2	≤ 1	≤ 1	≤ 1	≤ 1
Ceftazidime	4–16	= 16	= 16	= 16	= 16	= 16	= 8	≤ 4	≤ 4	≤ 4	≤ 4	≤ 4	≤ 4
Imipenem	1–8	≤ 1	≤ 1	≤ 1	≤ 1	≤ 1	≤ 1	≤ 1	≤ 1	≤ 1	≤ 1	≤ 1	≤ 1
Piperacillin/ Tazobactam	8/4-64/4	≤ 8	≤ 8	≤ 8	≤ 8	≤ 8	≤ 8	≤ 8	≤ 8	≤ 8	≤ 8	≤ 8	≤ 8
Amikacin	4–32	≤ 4	≤ 4	≤ 4	≤ 4	≤ 4	≤ 4	= 8	≤ 4	≤ 4	≤ 4	≤ 4	≤ 4
Gentamicin	0.25–8	> 8	> 8	> 8	> 8	> 8	> 8	= 4	= 1	= 0.5	= 1	= 0.5	= 0.5
Enrofloxacin	0.12–4	> 4	> 4	> 4	> 4	> 4	> 4	= 0.5	≤ 0.12	≤ 0.12	= 0.25	= 0.25	≤ 0.12
Marbofloxacin	0.12–4	= 4	> 4	= 4	= 4	> 4	= 4	= 0.25	≤ 0.12	≤ 0.12	≤ 0.12	≤ 0.12	≤ 0.12
Orbifloxacin	1–8	> 8	> 8	> 8	> 8	> 8	> 8	= 2	≤ 1	≤ 1	= 2	≤ 1	≤ 1
Pradofloxacin	0.25–2	> 2	> 2	> 2	> 2	> 2	> 2	≤ 0.25	≤ 0.25	≤ 0.25	≤ 0.25	≤ 0.25	≤ 0.25
Doxycycline	0.25–8	> 8	> 8	> 8	> 8	> 8	> 8	= 8	> 8	= 8	= 8	= 4	= 8
Tetracycline	4–16	> 16	> 16	> 16	> 16	> 16	> 16	= 8	> 16	> 16	> 16	> 16	= 16
Chloramphenicol	2–32	> 32	> 32	> 32	= 8	> 32	> 32	= 8	= 16	= 16	= 16	= 8	= 8
Trimethoprim/ Sulfamethoxazole	0.5/9.5-4/76	> 4	> 4	> 4	> 4	> 4	> 4	≤ 0.5	≤ 0.5	≤ 0.5	≤ 0.5	≤ 0.5	≤ 0.5

No resistances were noted for carbapenems and piperacillin/tazobactam in any of the isolates. As expected, resistances considered to be intrinsic to the genus *Serratia*, namely against ampicillin, amoxicillin-clavulanate, first generation cephalosporins and tetracycline, were observed in all isolates.

The genomic DNAs extracted from these 18 animal isolates were entirely sequenced using the Illumina platform and the reads were partially assembled into contigs in order to explore the presence of antimicrobial resistance genes (ARGs). The program ABRicate detected the ARGs *aac*(6’), *bla*SST/SRT and *tet*(41) in all isolates. Moreover, the 9 MDR strains were predicted to carry several supplementary ARGs, including the class A ESBL gene *bla*SHV-12, as well as resistance genes for aminoglycosides, tetracyclines and trimethoprim/sulfamides (see below).

Finally, sequence analysis of the predicted GyrA protein revealed the presence of a Serine-83-Isoleucine mutation commonly associated with Quinolone Resistance Determining Region (QRDR) in all 9 MDR isolates, and none of the other strains. The analysis of the ParC sequences did not reveal any mutation known to increase resistance to fluoroquinolones in any of the isolates.

### 2. Veterinary *Serratia* spp. isolates are placed in diverse genomic groups by Average Nucleotide Identity analysis

All animal isolates from U-Vet were originally identified as *S*. *marcescens* using the API rapid 32E kit, with minor variations in the biochemical profiles. The 9 MDR isolates were consistently noted as negative for D-Maltose and for 4-nitrophenyl-beta-D-galactopyranoside (PNPG) with this test, whereas the other isolates were all positive for these two traits, yielding two distinct groups of biocodes (Table 1).

**Fig 1:**
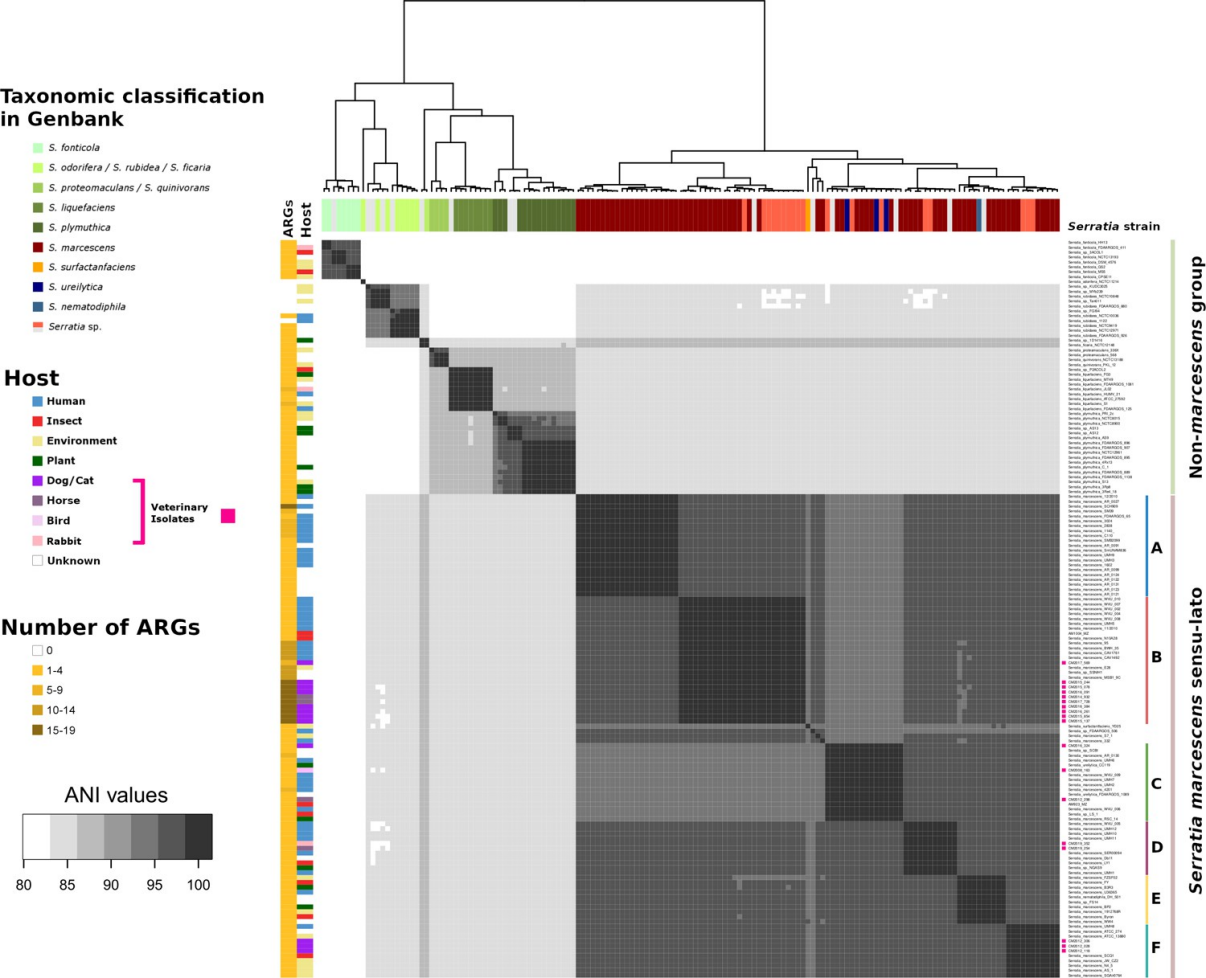
Heatmap of all-against-all ANI values for 133 complete *Serratia* spp. genomes and 18 veterinary isolates, showing distinct intra-specific subgroups within *S*. *marcescens sensu lato*. The top dendrogram was generated from a distance matrix calculated from the ANI values using the “dist” function (euclidean method) followed by hierarchical clustering using the “hclust” function (complete method) in R. The ANI values are represented by a scale of grey. The darkest shade represents ANI values >97.5%, which is used as the lowest limit to define groups A-F within the *S*. *marcescens sensu lato* group. The strain names and ANI groups are indicated on the right side of the map and their corresponding accession numbers are listed in the S1 Table. The GenBank files headers were used to extract the reported taxonomic classification (top sidebar) and the host or origin (left sidebar) of each strain, and this information was color-coded accordingly. The 18 animal isolates from the U-vet are indicated by a pink square on the right side of the map. The number of ARGs per genome was calculated from individual ABRicate reports and color-coded on the far-left sidebar.

To conclusively identify these isolates at the species level, pairwise Average Nucleotide Identities (ANI) of the entire genome sequences from the 18 animal isolates, 131 publicly available *Serratia* species (S1 Table), and 2 recently characterised insect isolates from the Melbourne Zoo were analysed. Hierarchical clustering of the all-against-all ANI values of each genome separated *S*. *marcescens* from other species, namely *S*. *fonticola*, *S*. *odorifera*, *S*. *rubidaea*, *S*. *ficaria*, *S*. *proteomaculans*, *S*. *quinovorans*, *S*. *liquefaciens* and *S*. *plymuthica* (Fig 1). The remainder of the genomes consisted predominantly of *S*. *marcescens* strains, but also contained 3 *S*. *ureilytica*, 1 *S*. *nematodiphila*, and several unclassified strains reported as “*Serratia* sp.” in the NCBI database. This ensemble of genomes, referred to as *S*. *marcescens sensu lato*, was tentatively sub-divided into 6 groups with intra-group ANI values ranging from 97.5% to 100%, hereafter named A to F, which were consistently higher than inter-group ANI values (Fig 1).

The group A contained only complete genomes from human isolates. The U-Vet animal isolates were present in the groups B, C, D, & F. The 9 U-Vet MDR isolates all fell in group B, which also contained human, environmental and insect isolates. These genomes were all predicted to carry high numbers of ARGs compared to all the other groups (Fig 1).

Moreover, 3 isolates (CM2008_163 from a bird, CM2012_298 from a horse, and CM2016_324 from a cat), which all fell in group C, were classified as *S*. *ureilytica*, an environmental species recently associated with insect mortalities. Two other publicly available *S*. *ureilytica* genomes were also present in the group C, one from a plant and one from an unknown origin.

### 3. Single Nucleotide Polymorphism analysis shows a quasi-clonal relationship among the MDR Serratia spp. isolated from diverse animals

**Fig 2:**
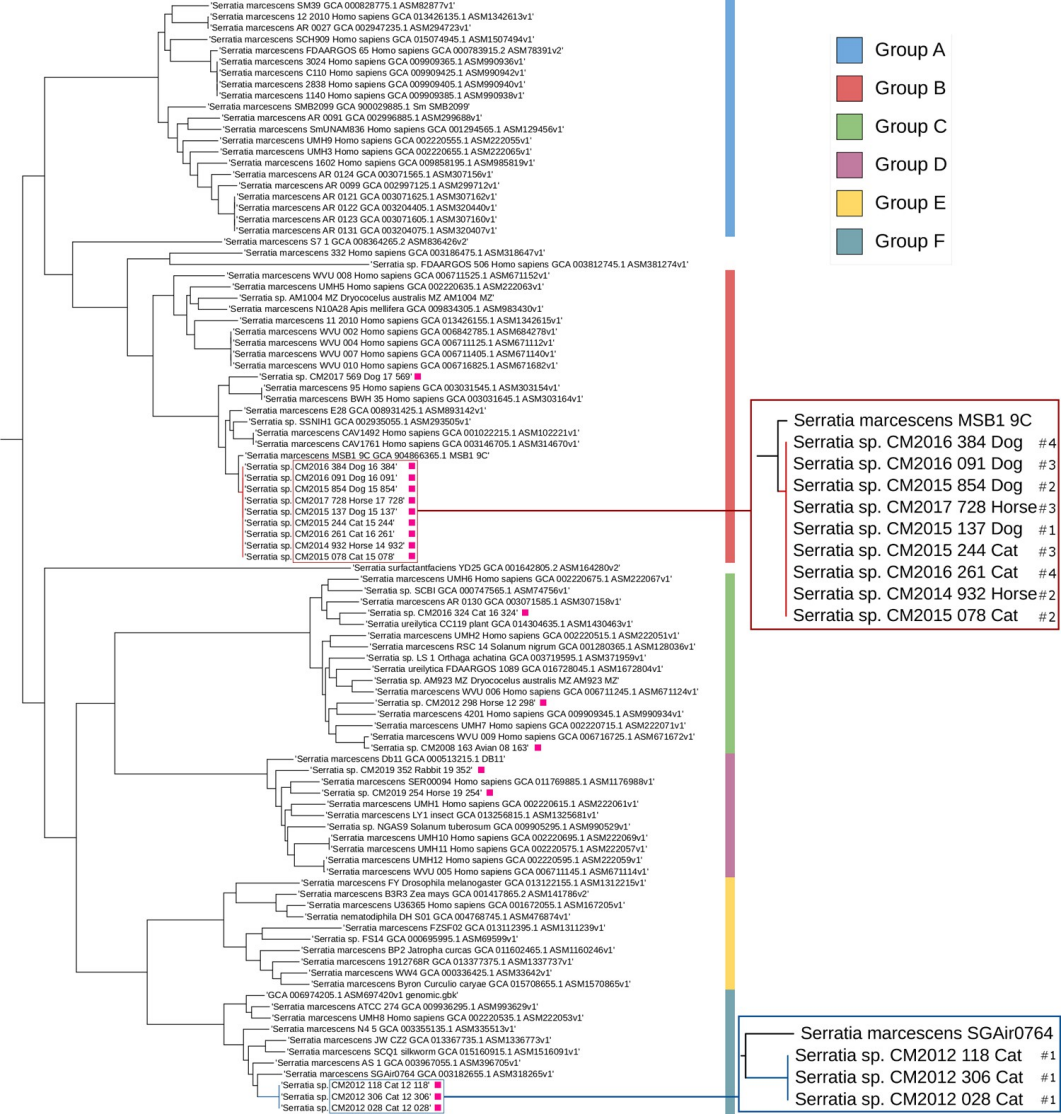
Core-genome phylogeny inferred from SNP analysis of the *S*. *marcescens* “*sensu-lato”* group, suggesting a quasi-clonal relation amongst the MDR isolates. An unrooted tree was generated from the multiple alignment containing high-quality SNP, Indels, and structural variations within the core genome, by Parsnp, from 81 complete genomes using strain ATCC_13880 as a reference and excluding potential recombination sites. The resulting tree was retrieved from the Parsnp output files and decorated with iTOL. The species, strain names, host and accession numbers extracted from GenBank are specified for each node. The 18 animal isolates from the U-Vet are indicated by a pink square.

The unrooted tree from core Single Nucleotide Polymorphisms (SNPs) present amongst the 18 U-Vet animal isolates and 81 *S*. *marcescens* “*sensu-lato”* complete sequences identified by ANI analysis supported the delineation of these genomes into the 6 groups previously found (Fig 2). Moreover, 2 groups of U-Vet animal isolates displayed quasi-clonal relationship in this tree. Firstly, in the group F, 2 out of the 3 isolates obtained from the same patient (cat #1) over a 2.5 month period did not show any SNP between them, while the third isolate carried only one nucleotide insertion and one SNP. Secondly, in the group B, the 9 MDR isolates were clustered in a clade with extremely short branch lengths (Fig 2).

The SNP analysis of the Illumina reads from the 9 MDR isolates with Snippy, using the canine strain CM2015_854 as the reference genome, revealed only 4 to 14 variations, mostly classified as SNPs or insertions, amongst the other strains (Table 3).

**Table 3:** SNP analysis of the group of MDR *Serratia* spp. isolates and comparison with their closest relative in our collection, strain CM2017_569.

TYPE	Strain vs. Reference CM15_854								
	CM2014_932	CM2015_078	CM2015_137	CM2015_244	CM2016_091	CM2016_261	CM2016_384	CM2017_728	CM2017_569
Single Nucleotide Polymorphism	4	3	7	2	6	3	7	8	15121
Insertion	5	1	6	3	4	1	5	3	24
Deletion							1	1	28
Combination of snp/mnp			1	1	1	1	1	1	1627
Multiple Nuclotide Polymorphism									132
Total	9	4	14	6	11	5	14	13	16932

The genome with the closest core SNP profile to these MDR isolates, *S*. *marcescens* strain MSB1_9C, was found in a human hospital ICU in Australia, and also carries multi-drug resistances (Fig 2). Only one other U-Vet isolate, CM2017_569, was present in the same ANI group B; however, its genome had significantly more SNPs relative to strain CM2015_854 (Table 3) and was placed more distantly in the tree (Fig 2).

### 4. Chlorhexidine resistance in Serratia spp. isolated from hospitalised animals supports a nosocomial origin of infections

The very low number of SNPs among the 9 MDR isolates, and their common origin from post-surgical complications from otherwise unrelated U-Vet patients, strongly suggested that these animals had been infected by the same strain circulating and persisting in the hospital premises. This prompted a review of all routine environmental surveillance reports collected in the U-Vet for the period 2014–2017.

One record indicated the isolation of a *S*. *marcescens* strain in a sample of chlorhexidine solution submitted on 23/03/2017 for sterility testing. The organism was identified with a rapid API 32E kit and was noted as D‐Maltose and PNPG negative, two traits which were also present in the 9 MDR strains from hospitalised animals. The isolate was not kept in collection at the time and therefore could not undergo genotyping, but this finding opened the hypothesis that the MDR strains were chlorhexidine-resistant and were transmitted during surgical procedures by the disinfectant solutions, causing sporadic nosocomial infections.

To test this hypothesis, the *Serratia* strains from U-Vet were assessed for their ability to grow in broth containing increasing concentrations of chlorhexidine. The individual MIC and Minimum Bactericidal Concentration (MBC) of these isolates were compared to an *E*. *coli* control strain and a multi-drug resistant *Enterobacter hormaechei* strain, CM2018_216, found recently in a sink of the same hospital. All 9 MDR strains, as well as strain CM2017_569 (which is susceptible to fluoroquinolones and does not produce ESBLs, but falls into the group B like the other MDR strains) were able to grow in a 1/4 dilution of the 1% chlorhexidine solution (i.e. at 0.25%), while the growth of the non-MDR strains was visibly inhibited at or below a 1/128 dilution (i.e. 0.008%), with the exception of strain CM2008-163, which grew in the disinfectant up to the 1/64 dilution. The MBCs were also above 0.25% for all MDR strains and CM2017-569 but ranged from 0.004% to 0.125% for the other strains (Table 4).

**Table 4:** Chlorhexidine Minimum Inhibitory Concentrations and Minimum Bactericidal Concentrations for *Serratia* spp. isolates, showing a higher resistance to this biocide within the MDR strains compared to the other strains.

Strain^1^	MIC^2^	MBC^3^
*E*. *coli* K12 (Control)	≤ 0.0002%	≤ 0.0002%
CM2008_163 (*)	= 0.031%	= 0.125%
CM2012_028	= 0.001%	= 0.004%
CM2012_118	= 0.001%	= 0.004%
CM2012_306	= 0.001%	= 0.004%
CM2014_932 (MDR)	> 0.25%	> 0.25%
CM2015_078 (MDR)	> 0.25%	> 0.25%
CM2015_137 (MDR)	> 0.25%	> 0.25%
CM2015_244 (MDR)	> 0.25%	> 0.25%
CM2015_854 (MDR)	> 0.25%	> 0.25%
CM2016_091 (MDR)	> 0.25%	> 0.25%
CM2016_261 (MDR)	> 0.25%	> 0.25%
CM2016_324 (*)	= 0.004%	= 0.063%
CM2016_384 (MDR)	> 0.25%	> 0.25%
CM2017_728 (MDR)	> 0.25%	> 0.25%
CM2017_569 (**)	> 0.25%	> 0.25%
CM2019_254	= 0.008%	= 0.031%
CM2019_352	= 0.008%	= 0.004%
*E*. *ho*. CM2018_216	= 0.002%	= 0.008%

1: Strain: A single asterisk denotes *S*. *ureilytica*; a double asterisk denotes the chlorhexidine-resistant, non-MDR strain CM2017_569; *E*. *ho* denotes *E*. *hormaechei* strain CM2018_216.

2: MIC: Minimum inhibitory concentration.

3: MBC: Minimum bactericidal concentration.

**Fig 3:**
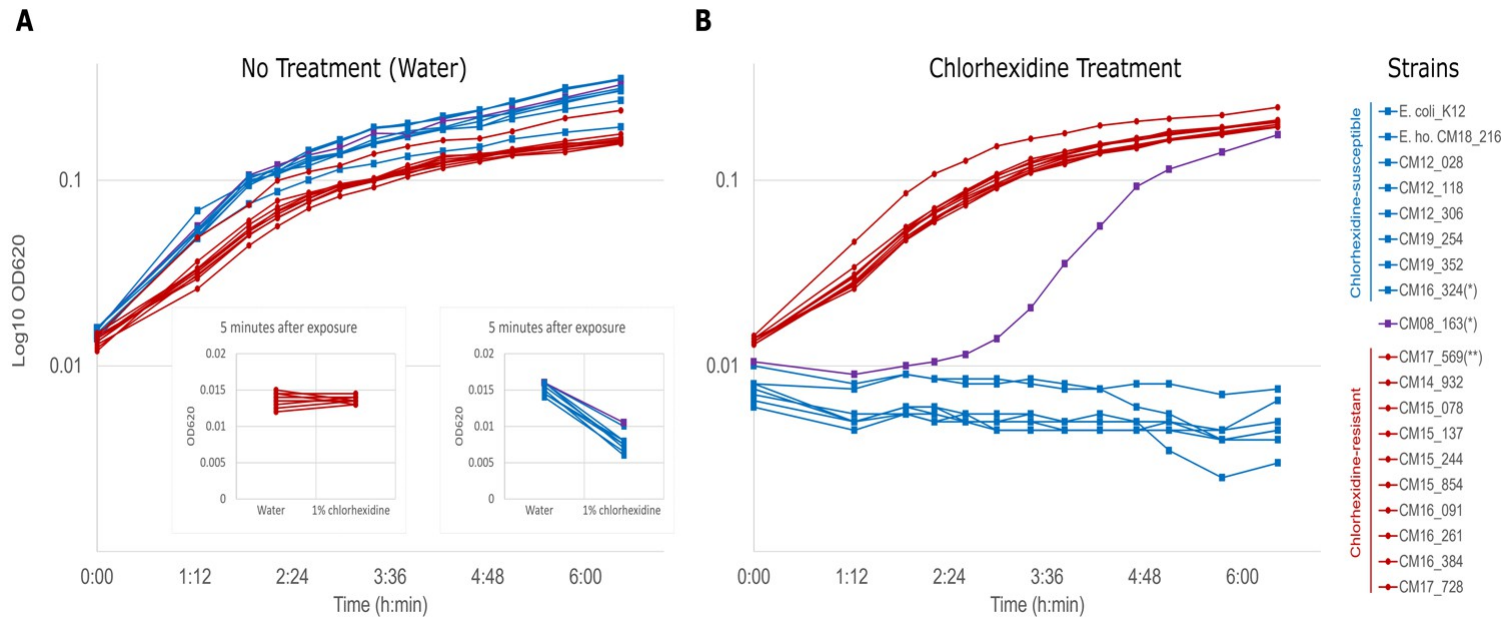
Effect of chlorhexidine exposure on the growth of *Serratia* isolates from the U-Vet, indicating the ability of the resistant strains to recover from routine disinfection protocols. Duplicate cultures of each isolate were incubated in a 96-well plate. The average of the two OD620nm values after blank subtraction are plotted against time on a log-transformed scale. The single asterisk * indicates *S*. *ureilytica* isolates. A: Bacterial suspensions exposed to sterile water for 5 minutes, then diluted 1:10 in Letheen Broth. B: Bacterial suspensions exposed to 0.5% chlorhexidine for 5 minutes, then diluted 1:10 in Letheen broth. Red lines, filled circles: Chlorhexidine-resistant *Serratia* isolates including all 9 MDR isolates and the non-MDR strain CM2017_569 (double asterisk **). Blue lines, filled squares: Chlorhexidine-susceptible *Serratia* strains, *E*. *hormachei* strain CM2018_216, and control strain *E*. *coli* K12. Purple line, filled squares: *S*. *ureilytica* strain CM2008_163, showing intermediate resistance to Chlorhexidine. Insets indicate the OD620nm values shortly after the dilution step in Letheen broth (time 0).

To better mimic the conventional use of chlorhexidine in veterinary settings, which can be applied on skin or other surfaces and rinsed or removed shortly after contact, the MIC/MBC results were confirmed by assessing the ability of the *Serratia* spp. U-Vet isolates to recover and grow after 5 minutes exposure to 1% chlorhexidine solution, followed by neutralisation/dilution of the disinfectant with Letheen broth (Fig 3). As a negative control, the same strains were treated with sterile water, instead of 1% chlorhexidine (Fig 3A).

In the first hour following the disinfectant treatment, the MDR *Serratia* strains, as well as strain CM2017_569, started to grow in Letheen broth. By contrast, no growth was observed with all other strains as well as an *E*. *coli* K12 control strain, with the exception of *S*. *ureilytica* strain CM2008_168 which exhibited a delayed growth of approximately 3.5 hours (Fig 3B). Comparative individual analysis of the growth curves by the R package ‘growthcurver’ confirmed that all chlorhexidine-resistant strains had very similar growth characteristics to each other, regardless of being initially exposed to water or to the disinfectant, whereas the chlorhexidine-susceptible strains grew only after the water treatment, with variable kinetics (S2 Fig).

Between the first and second hour following exposure, the 9 MDR isolates and the strain CM2017_569 had estimated doubling times that were similar for chlorhexidine and water treatments, with averages of 53 minutes (range 49–65) and 46 minutes (range 44–53), respectively. By contrast, no growth was apparent for the other isolates in the chlorhexidine group, but the same isolates displayed an average doubling time of 41 minutes (range: 37–43 minutes) under the no-treatment (water) condition.

Immediately after treatment, the OD_620nm_ of the chlorhexidine-susceptible strains was lower in the disinfectant condition compared to the water-only condition, while the chlorhexidine-tolerant strains had similar OD_620nm_ values regardless of the treatment group (Fig 3A insets).

### 5. The MDR isolates carry a promiscuous IncHI2 plasmid prevalent in *Enterobacteriaceae*, but rarely found in *Serratia* genomes

**Fig 4:**
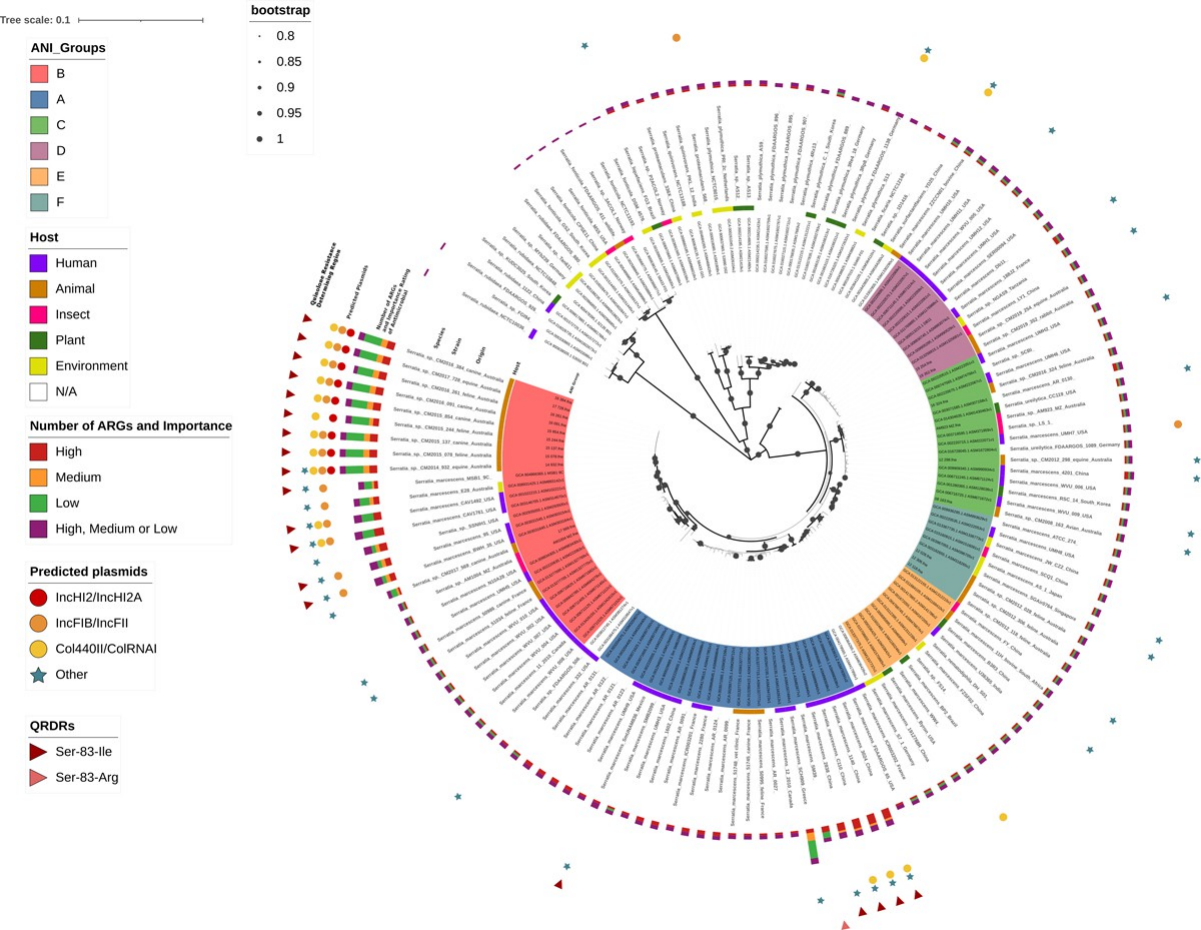
Phylogenetic analysis of *Serratia* spp. genomes, demonstrating the distribution of unusual antimicrobial resistances patterns and plasmid incompatibility groups in phylogenetically related strains. A maximum likelihood tree was generated from 147 concatenated multiple alignments of 13 house-keeping genes representing 19074 positions with MEGA using the General Time Reversible model with discrete Gamma distribution and Invariable sites (GTR+G+I), selected by the lowest Bayesian Information Criterion (BIC) score. A bootstrap consensus tree was inferred from 100 replicates. Bootstrap values are represented by branch thickness and circles with proportional diameter. The presence of ARGs and/or plasmids in the genomes were predicted by ABRicate using the databases resfinder and plasmidfinder, respectively. The unrooted tree was decorated with iTOL. Nodes indicate the species, strain and origin of each isolate as reported in the corresponding GenBank file. Bars represent the number of ARGs with importance rating. Circles or stars represent the incompatibility group of plasmid(s). Triangles indicate the presence of a Quinolone Resistance Determining Region (QRDR) in the GyrA protein sequence.

To better describe the diversity of veterinary *Serratia* isolates and confirm the ANI and core SNP results, a phylogenetic tree was constructed from 147 concatenated sequence alignments of conserved genes, which were extracted from the the U-Vet isolates and the set of 131 complete genomes previously analysed supplemented with a further 11 partial genomes from animals downloaded from the NCBI database (S2 Table). The *S*. *marcescens sensu lato* strains were separated from the other species and grouped into individual clades representing the 6 ANI groups previously described (Fig 4). As expected, the 9 U-Vet MDR isolates were all placed in a single branch of highly related genomes within the group B.

Since resistance to fluoroquinolones was a concerning feature of the 9 MDR isolates for animals, other publicly available *Serratia* genomes were searched to detect similar sequence signatures to evaluate their frequency of occurrence in this genus. Chromosomal QRDRs (which are not predicted by Abricate) were assessed in a sequence alignment of the protein GyrA extracted from all 131 complete genomes previously examined. The Serine 83 was replaced by an Isoleucine in only a few closely related strains from group A, i.e. 4 human isolates from China, and from group B, i.e. 1 human hospital environmental isolate from Australia, and 4 human isolates from the USA (Fig 4). In addition, a single isolate, SM39, possessed an arginine residue at the same position. Other mutations associated with quinolone resistance in GyrA and ParC were not identified in the genome dataset. Overall, chromosomal mutations conferring quinolone resistance appeared to be relatively rare in published *Serratia* spp. genomes and were confined to a few human clinical isolates and the present collection of animal isolates.

Isolates predicted by ABRicate to carry high numbers of ARGs (up to 20) were clustered within a sub-clade in the group B, which included the 9 U-Vet MDR strains and 7 human or environmental isolates (2 from Australia and 5 from the USA). In the group A, one human isolate from Greece found in 1988 was also predicted to carry 19 ARGs (Fig 4). Sequence searches using the plasmid-finder database in ABRicate revealed that most of these multi-resistant isolates carried Col440II/ColRNAI plasmids (in both the groups A and B) and IncFIB/IncFII plasmids (in the group B). Furthermore, a third type of plasmid, classified as IncHI2/IncHI2A, was predicted to be present in the 9 MDR U-Vet isolates, but in none of the other 131 genomes from NCBI.

The genomes of 5 U-Vet strains representing the different groups identified through phenotypic and phylogentic analyses were completely circularised using both Illumina and Nanopore reads, in order to ascertain the localisation of ARGs on replicons. In the group B, the MDR strain CM2015_854 genome was assembled into a 5556144 bp chromosome and 5 plasmids: a 295330 bp IncHI2, a 193603 bp IncFIB, and three cryptic plasmids (4820 bp Col440II, 4357 bp Rep1 and 3223 bp ColRNAI). The strain CM2017_569 posessed a 5228298 bp chromosome and a 4747 bp Col(Ye4449) plasmid. No plasmid was identified in the strains CM2016-324 (group C) CM2019_254 (Group D) and CM2012_028 (group F), which possessed respectively a 5172927 bp, 5099636 bp and 5052702 bp chromosome as sole replicon. The IncHI2 plasmid found in strain CM2015_854 carried 15 ARGs, including the ESBL gene *bla*SHV-12, whereas all other genomes had only predicted ARGs on their chromosomes (Table 5).

**Table 5:** Predicted ARGs in fully assembled genomes of representative *Serratia* spp. isolates, showing the high number of predicted resistance markers carried by a IncHI2 plasmid in strain CM2015_854.

Location	Gene	Group B		Group C	Group D	Group F	Total
		CM2015_854	CM2017_569	CM2016_324	CM2019_254	CM2012_028	
Chromosome	*aac*(3)-Ia		1				1
Chromosome	*aac*(6’)	1	1	1	1	1	5
Chromosome	*aadA*6		1				1
Chromosome	*blaSST*/*SRT*	1	1	1	1	1	5
Chromosome	*oqxB*	1	1	1	1	1	5
Chromosome	*sul*1		1				1
Chromosome	*tet*(41)	1	1	1	1	1	5
**sub-total **		**4**	**7**	**4**	**4**	**4**	**23**
IncHI2 Plasmid	*aac*(3)-II	1					1
IncHI2 Plasmid	*aac*(6’)-Iic	1					1
IncHI2 Plasmid	*aad*A2	1					1
IncHI2 Plasmid	*aph*(3’)-Ia	1					1
IncHI2 Plasmid	*aph*(3”)-Ib	1					1
IncHI2 Plasmid	*aph*(6)-Id	1					1
IncHI2 Plasmid	*arr*	1					1
IncHI2 Plasmid	*bla*SHV-12	1					1
IncHI2 Plasmid	*bla*TEM-1	1					1
IncHI2 Plasmid	*dfrA*19	1					1
IncHI2 Plasmid	*ere*(A)	1					1
IncHI2 Plasmid	*mcr*-9.1	1					1
IncHI2 Plasmid	*sul*1	2					2
IncHI2 Plasmid	*tet*(D)	1					1
**sub-total**		**15**	** **	** **	** **	** **	**15**
**Total**		**19**	**7**	**4**	**4**	**4**	**38**

Since IncHI2 plasmids were not detected in any of the other 131 complete *Serratia* spp. genomes examined previously, a larger set of 671 complete and partial *S*. *marcescens* genomes was downloaded from the NCBI database and a search for plasmids belonging to the same incompatibility group, using ABRicate and the database plasmid-finder was conducted. Only 6 other human clinical isolates (from Romania, the USA and Austria) were predicted to carry an IncHI2/IncHI2A plasmid (Table 6).

**Table 6:** Sequenced humans isolates of *Serratia* that are predicted to carry a IncHI2/IncHI2A plasmid, illustrating the rarity of the incompatibility group within this genus.

Accession	Species	Strain	Host	Source	Country	Year	No ARGs
GCA_900108835.1_SM1978	Serratia_marcescens	SM1978		clinical	_	2016	20
GCA_002738185.1_ASM273818v1	Serratia_marcescens	9580	Homo_sapiens	urine	Romania	2015	18
GCA_002886905.1_ASM288690v1	Serratia_marcescens	YDC107_2	Homo_sapiens		USA	-	18
GCA_002887105.1_ASM288710v1	Serratia_marcescens	YD509_2	Homo_sapiens		USA	-	18
GCA_002250685.1_ASM225068v1	Serratia_marcescens	at10508	Homo_sapiens	lavage	Austria	2017	15
GCA_007954045.1_ASM795404v1	Serratia_marcescens	KCJ3K309	Homo_sapiens		USA	2019	14

Five of these strains also carried high numbers of ARGs and were phylogenetically related to the U-Vet Australian animal strains, albeit more distantly (S1 Fig). These results strongly suggested that IncHI2 plasmids are rarely present in *Serratia* spp. genomes. An online search was performed on the Mash_Dist Plasmid Database (PLSDB) server, using the IncHI2 plasmid from strain CM2015-845 as a query sequence. This returned 405 hits in various bacterial genera, including *Escherichia* (n = 151), *Salmonella* (n = 127), *Enterobacter* (n = 75), *Klebsiella* (n = 21), *Citrobacter* (n = 12), and *Leclercia* (n = 7). Only one isolate of *Serratia* carrying a IncHI2 plasmid (NCBI Reference Sequence NC_005211.1, plasmid R478) was identified by the PLSDB database, suggesting that the MDR isolates were derived from a *Serratia* strain that had acquired this IncHI2 plasmid within the hospital and had persisted as a quasi-clonal population in the chlorhexidine solutions.

Finally, the high MICs and MBCs values of chlorhexidine in strain CM2017-569, which is phylogenetically close to the 9 MDR isolates but does not possess an IncHI2 plasmid and has a different antimicrobial resistance profile, indicated that the disinfectant tolerance observed previously was not dependent on the plasmid, and may be controlled by other determinants.

### 6. The IncHI2 plasmid from MDR *Serratia* isolates is highly similar to a conjugative plasmid from *Enterobacter* spp. previously isolated in the same veterinary hospital

The discovery of a widely promiscuous IncHI2 plasmid in the 9 MDR *Serratia* isolates from animal patients treated at the U-Vet prompted us to compare its sequence to the conjugative IncHI2 plasmid pCM2018-416 (GenBank accession CP050312.1), which was characterised in an environmental strain of *Enterobacter hormaechei* previously found in a handwashing sink of the Hospital, as well as similar plasmid sequences downloaded from the online PLSDB repository.

**Fig 5:**
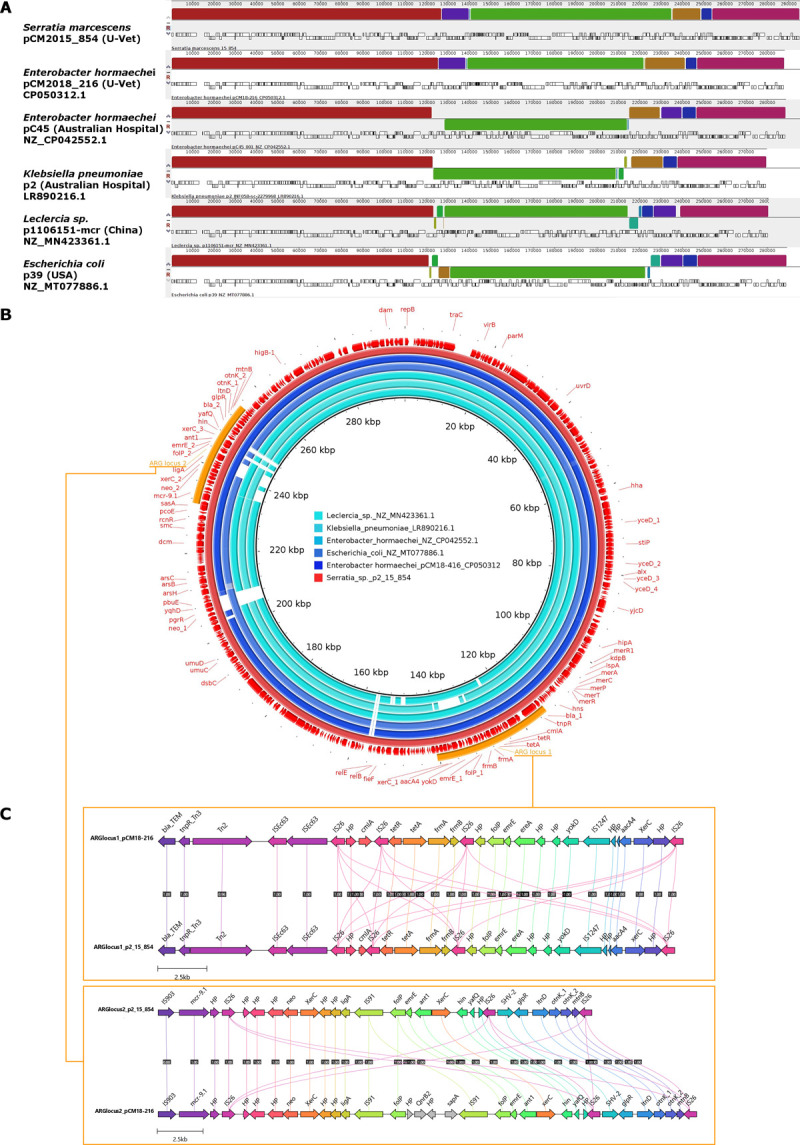
Comparison of IncHI2 plasmids from MDR *Serratia* spp., *E*. *hormachei*, *Klebsiella pneumoniae* and *E*. *coli*, showing the high similarity between the two replicons found at the U-Vet, and suggesting a common origin for this mobile genetic element within the hospital. A) Mauve sequence alignments, showing local blocs of co-linearity and structural conservation between plasmids pCM2015_854 from *Serratia* (top), pCM2018_216 from *E*. *hormachei* (second top) and other most-closely related plasmids. B) Comparative alignments of plasmid sequences by BRIG showing the conservation of antimicrobial resistance loci. The four outermost rings (red-orange) correspond to the plasmid carried by the representative MDR *Serratia* strain CM2015_854, used as a reference. The inner rings (shades of blue) represent highly similar plasmids from other *Enterobacteriaceae*. From outer to inner rings: 1- plasmid pCM2015_854 annotations (red), 2- ARG loci (orange), 3- map of annotated features (red), 4- nucleotide sequence (red), 5- plasmid pCM2018_216 sequence (dark blue), 6- to 8- other plasmids sequences (medium to light blue). C) Local genetic maps of ARG loci of plasmids pCM2015_854 from *Serratia* and pCM2018_216 from *E*. *hormachei*.

Sequence alignments revealed that the *Serratia* and *Enterobacter* plasmids from the U-Vet were highly similar to each other, showing strong structural co-linearity and encoding conserved repertoires of ARGs across two loci identically positioned on the replicon (Fig 5). By contrast, the four closest plasmids identified in public databases by the Mash-Dist PLSDB server, namely pC45, p2, p1106151-mcr and p39, carried respectively by *E*. *hormaechei*, *Klebsiella pneumoniae*, *Leclercia* spp. and *E*. *coli*, all had large inversions and rearrangements compared to the two resistance plasmids from the U-Vet (Fig 5A). The plasmid sequences pC45 and p2 also originated from an Australian hospital, while p1106151 from China and p39 from the USA. All six plasmids had similar backbones, but some genetic variability was observed within the ARG loci (Fig 5B).

The two plasmids from the U-Vet had a nearly identical organisation, except for 2 short segments. Firstly, a 8.7 kb sequence encoding the aminoglycoside 3’-phosphotransferase Neo, HTH-type transcriptional regulator PgrR, alcohol dehydrogenase YqhD and purine efflux pump PbuE was present only in pCM2015_854 from *Serratia*. This region was surrounded by two tandem copies of the ISNCY family transposase *ISBcen27* while a single copy was present in the *E*. *hormachei* plasmid. Secondly, a 2.7 kb sequence carrying the quinolone resistance marker *qnrB2* was present solely in the ARG locus-2 of pCM2018-416 *E*. *hormachei*. This region was surrounded by two direct repeated sequences containing the transposase IS91, while a single copy of the same IS91-carrying sequence was present in the *S*. *marcescens* plasmid (Fig 5C). These two differences suggested that limited deletions/insertions recombinations had occurred in the two replicons found in the U-Vet.

Of note, the *E*. *hormachei* strain harbouring the IncHI2 plasmid in U-Vet was readily inactivated by chlorhexidine (Table 4 and Fig 3), whereas the *S*. *marcescens* strain CM2017-569, which could survive in the disinfectant and was phylogenetically related to, but distinct from, the nine plasmid-carrying MDR clonal isolates, did not possess this replicon nor displayed a multi-drug resistance phenotype. This observation suggested that an antimicrobial-susceptible, chlorhexinine-tolerant strain of *Serratia* could have aquired the IncHI2 resistance plasmid from an *Enterobacteriaceae* such as the *E*. *hormachei* strain present in the hospital environment, and had persisted in the disinfectant.

The results of a mating experiment performed with the donor strain *E*. *hormachei* CM2018_216 and the recipient strain *S*. *marcescens* CM2017_569 supported this hypothesis. Following dual selection by chlorhexidine and third generation cephalosporins, 2 randomly selected potential transconjugants, T4 and T6, were purified and identified as *S*. *marcescens* using the rapid API32E kit, showing the same biocode as the recipient strain CM2017_569. Moreover, T4 and T6 had a high MIC value with amikacin (8 ug/mL), a feature uniquely present in CM2017_569 within the strain collection (Table 2). The transconjugants also displayed ESBL production phenotypes on plates (S3 Fig) and had high MICs with third generation cephalosporins, chloramphenicol, gentamicin and trimethoprim/sulfamide, indicating that the *E*. *hormachei* resistance plasmid pCM2018_216 had been transferred into the recipient strain CM2017_569 (Table 7).

**Table 7:** Comparison of MICs values (μg/mL) of donor strain *E*. *hormachei* CM2018_216, recipient strain *S*. *marcescens* CM2017_569 and 2 *Serratia* transconjugants T4 and T6, demonstrating the occurrence of transfer of resistance *in vitro*.

Antimicrobic	E. hormaechei CM2018_216	S. marcescens CM2017_569	Transconjugant T4	Transconjugant T6
Amikacin	≤ 4	= 8	= 8	= 8
Cefovecin	> 8	= 4	> 8	> 8
Cefpodoxime	> 8	= 2	> 8	> 8
Ceftazidime	> 16	≤ 4	> 16	> 16
Chloramphenicol	> 32	= 8	> 32	> 32
Gentamicin	> 8	= 4	> 8	> 8
Trimethoprim/Sulfamethoxazole	> 4	≤ 0.5	> 4	> 4

### 7. Two distinct strains of chlorhexidine-tolerant *Serratia* sp. share putative mobile genetic elements carrying biocide resistance genes

Although plasmid sequences were detected in the strains CM2015_854 and CM2017_569, no extra chromosomal replicons were predicted to be present in the other *Serratia* strains. To explore the presence of other Mobile Genetic Elements such as prophages and Integrative Conjugative Element (ICEs), the online tools IslandViewer4, Phaster, and ICEFinder were used to analyse the fully assembled chromosomal sequences of strains CM2015_854, CM2017_569, CM2016_324, CM2019_254, and CM2012_028.

The strain CM2015_854 was predicted to contain a 47.2Kb intact prophage, as well as 11 other regions carrying various incomplete phage sequences, and 3 CRISPR regions with 12, 40 and 72 repeat units. Strain CM2017_569 was not predicted to harbor an intact prophage, but it carried 6 partial phage sequences. Strains CM2012_028, CM2019_254, and CM2016_324 were all predicted to contain one intact prophage each, of 40.1 kb, 34.8 kb, and 45.7 kb, as well as 3, 1 and 2 partial phage sequences, respectively. The strain CM2015_854 was also predicted to possess four ICEs with a Type 4 secretion system (T4SS) of 111 kb, 83 kb, 79 kb and 88 kb on its chromosome. Three of these regions were also predicted to carry incomplete phage sequences. Strain CM2017_569 was predicted to possess two ICE-containing chromosomal regions of 72 kb and 245 kb, the larger region also carrying an incomplete phage sequence. Furthermore, one 92 kb putative ICE was found in strain CM2016_324, while no ICEs were found in strains CM2012_028 and CM2019_254.

**Fig 6:**
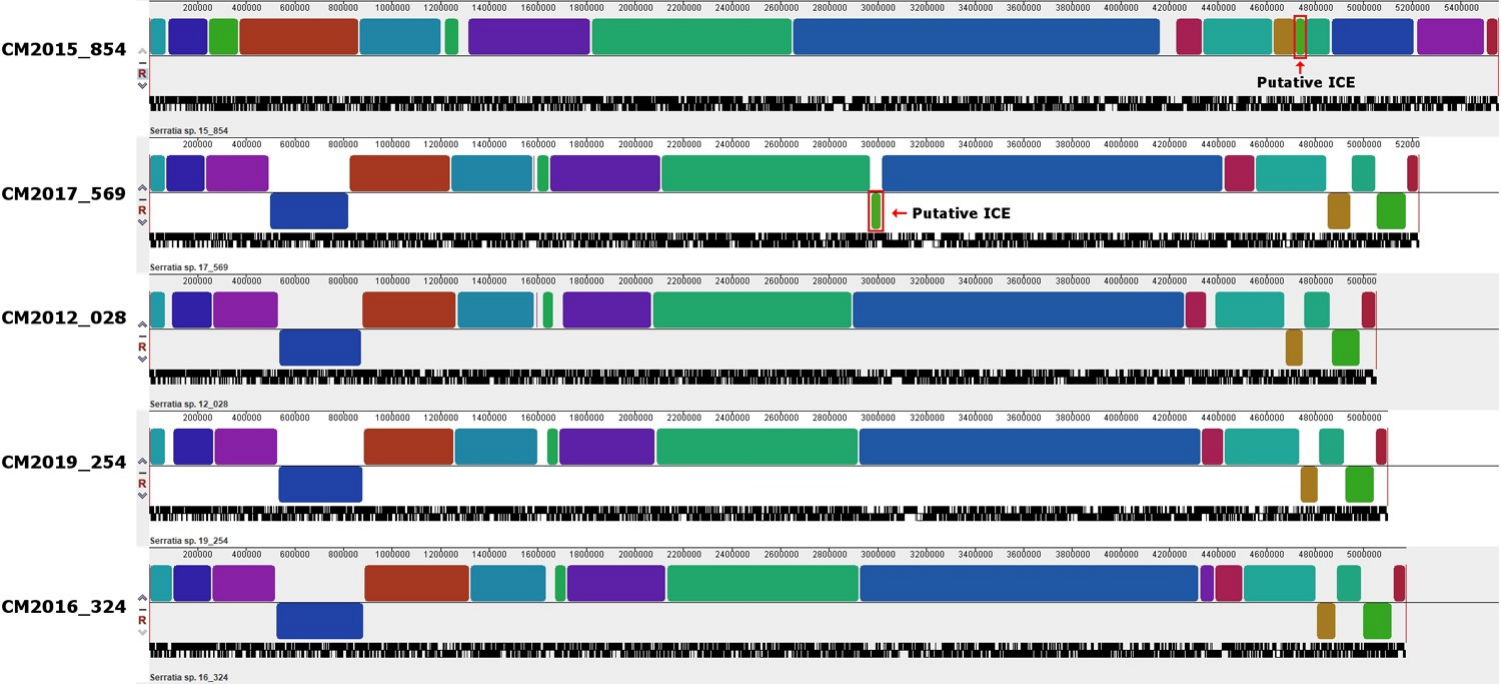
Progressive Mauve alignment of the chromosomes from representative U-vet *Serratia* isolates, showing the presence of an Integrative Conjugative Element in two chlorhexidine-tolerant strains. The position of the predicted ICE region in CM2015_854 and CM2017_569 is indicated by the red box. Locally colinear blocks are indicated in solid colors.

A Progressive-Mauve alignment of the chromosomes revealed that both chlorhexidine-tolerant strains CM2015_854 and CM2017_569 possessed a nearly identical chromosomal region of 43878 bp which was absent from the chlorhexidine-susceptible strains. This region was embedded in the 88 kb and 245 kb regions that carried a putative ICE in the 2 strains and contained genes controlling resistance to biocides. Apart from the presence of this ICE-associated region in CM2017_569, the chromosomes of all four non-MDR strains (CM2017_569, CM2016_324, CM2019_254, and CM2012_028) were substantially co-linear. By contrast, the MDR strain CM2015_854 chromosome showed various rearrangements of sequence blocks and it was significantly longer compared to the other strains (Fig 6).

Furthermore, a pan-genome analysis of the 5 genomes by the program Roary identified 139 protein sequences with a putative function (i.e. excluding hypothetical proteins), which were present in the 2 chlorhexidine-tolerant strains CM2015_854 and CM2017_569, but absent from the four others genomes. Out of these 139 sequences, 41 were associated with genomic islands carrying phages and/or ICEs sequences (S4 Table). The function annotation of these 139 sequences by KofamKOALA revealed that 11 genes, all present within predicted genomic islands, were putatively involved in resistance to biocides and/or encoded multidrug efflux systems. In particular, four gene products encoded by the above-mentioned 43878bp ICE-associated region were homologous to bacterial multispecies proteins conferring resistance to biocides, namely the major facilitator superfamily (MFS) methyl viologen transporter (WP_023123531) and the tripartite resistance-nodulation-cell division (RND) efflux system consisting of a periplasmic adaptor (WP_055315664), a permease (WP_055312464), and an outer membrane subunit (WP_060431991). Finally, a search of all predicted coding sequence products from the U-Vet strains as query sequences against a database of 753 experimentally confirmed biocide resistance proteins with BlastP showed that these four proteins had sequence matches with the MFS efflux pump AmvA (AedF) from *Acinetobacter baumannii*, the RND membrane fusion protein MexC, multidrug transporter MexD, and outer membrane protein OprJ from *Pseudomonas aeruginosa* in the chlorhexidine-tolerant strains only.

## Discussion

*S*. *marcescens* is a well-known nosocomial pathogen, causing disease outbreaks in human hospitals [7], with important concerns for high-risk settings such as surgical or neonatal intensive care units [42,43]. Most *Serratia* genomes represent human clinical isolates or originate from plant, insects or the environment. By comparison, very few isolates have been characterised in domestic animals. This study demonstrated the association of phylogenetically diverse strains of *S*. *marcescens* and *S*. *ureilytica* with opportunistic and/or sporadic infections in a wide range of companion animals. While *S*. *marcescens* infections have been reported in dogs and cats [6], *S*. *ureilytica* is mainly considered as an environmental organism [44] and little is known on its virulence on domestic or wild animals. The 3 strains of *S*. *ureilytica* described in this study were isolated from potentially contaminated sites in a bird (necrotic hepatitis), a cat (free catch urine), and a horse (guttural pouch). Therefore, it is unclear whether this species is a primary pathogen of veterinary importance. By contrast, the isolations of *S*. *marcescens* in moderate to heavy pure cultures from post-surgical complications in various animals, and from urinary infections in a cat and a dog, confirms the pathogenic potential of these strains.

The persistence of *Serratia* spp. in disinfectants such as chlorhexidine has been reported in human [5] and veterinary [6] hospitals. The ability to survive in biocides creates additional challenges for the prevention and control of nosocomial infections in clinical care environments. This problem is compounded by multi-drug resistance, particularly if high importance rating antimicrobials are affected. Most *Serratia* genomes appear to carry modest numbers of ARGs. Our results confirmed this trend, with half of the isolates in the collection susceptible to medium and high importance antimicrobials commonly used to treat animals. However, in this study we discovered a group of quasi-clonal multi-drug resistant *S*. *marcescens* isolated from post-surgical infections. The isolates survived in the presence of chlorhexidine and were able to grow normally after a 5 mins exposure to the disinfectant. These results suggest that chlorhexidine-based conventional disinfection protocols used prior to surgery would not have inhibited the growth of the *Serratia* involved in the complications. The sporadic nature of the cases, wide range of infected animals, diversity of owners, and long durations between microbiological cultures made it very difficult to recognise the ongoing transmission of *Serratia* while it was occurring, despite active and passive infectious risk surveillance programs in place in the veterinary hospital. This highlights the importance of keeping excellent patient records and extensive collections of clinical isolates to conduct retrospective investigations on the historical linkages of potentially nosocomial infections.

The presence in veterinary premises of promiscuous plasmids and other mobile genetic elements carrying high importance ARGs should be taken seriously, because of the risks of dissemination and accumulation of multiple resistances into a wide range of microorganisms. The present study strongly suggests that a promiscuous IncHI2 plasmid encoding resistances against several antimicrobials of high importance was acquired by a *Serratia* strain in the hospital environment, through conjugation with a different bacterial species. While colistin resistance is intrinsic in *S*. *marcescens* and therefore the role of *mcr*-9.1 was not explored here, the presence of the ESBL gene *bla*SHV-12 on the IncHI2 plasmid was of particular concern, because third generation cephalosporins are used occasionally in companion animals and resistances to this high importance antimicrobial should be closely monitored. One of the few IncHI2 MDR plasmids characterised in a *Serratia* clinical isolate is R478, which was found in the 1960’s in a hospital patient [45]. The plasmid R478 confers resistance to tetracyclines, chloramphenicol, aminoglycosides and heavy metals but does not encode ESBLs. The origin of the IncHI2 MDR plasmid in the 9 clonal *Serratia* isolates reported in this study is difficult to ascertain, but it is worth noting that IncHI2 plasmids possess a wide range of bacterial hosts in the *Enterobacteriaceae* family whereas they have been rarely sequenced in *Serratia* spp., a member of the *Yersiniaceae* family. Since IncHI2 plasmids are commonly found in *Enterobacter* spp., a simple scenario is that the MDR *E*. *hormachei* carrying the IncHI2 plasmid discovered in the ICU hand wash sink [13] had previously contaminated the hospital and persisted undetected in the environment for several years, acting as an environmental reservoir and a conjugative donor for a *Serratia* strain present in the same premises. Equally, it is possible that both *Enterobacter* and *Serratia* independently acquired the IncHI2 plasmid from another environmental donor, yet to be identified, in the same premises.

Biocide efflux in *Serratia* spp. is controlled by various mechanisms, including the MFS transporter SmfY [46], ABC transporter SmdAB [47], and RND systems similar to the AcrAB-TolC [48]. The *Serratia* RND pumps include SdeAB [49], SdeCDE [50], and SdeXY [51]. These systems also involve the outer membrane protein HasF, a TolC homolog, and the expression regulator SdeR, a MarA homolog [52]. All *Serratia* isolates characterised in this study were predicted to harbour several types of RND efflux systems. However, a remarkable feature of the chlorhexidine-tolerant strains, i.e. CM2015_854 and CM2017-569, was the presence of a genomic island encoding homologs of the MFS transporter AmvA and the RND biocide efflux pump MexCD-OprJ. The expression of *amv*A in *A*. *baumanii* and *E*. *coli* confers a modest increase of resistance to chlorhexidine [53]. In *P*. *aeruginosa*, the tripartite RND efflux system MexCD-OprJ is induced by, and confers resistance to, chlorhexidine [54]. Therefore, it is possible that the homologs of these proteins in the chlorhexidine-tolerant *Serratia* contribute to their ability to survive within the disinfectant and to cause sporadic infections following surgery. The exploration of the transmissibility of the RND MexCD-OprJ system and its role in biocide resistance among strains of *Serratia* spp. is beyond the scope of this study, and will require further investigations.

It is difficult to determine whether the precursor of the *Serratia* clone which acquired the MDR IncHI2 plasmid was chlorhexidine-tolerant before or after the plasmid transfer. The isolation of strain CM2014_932 from a horse provides evidence of the acquisition of an IncHI2 plasmid by a *Serratia* host as early as, or before, December 2014. While no chlorhexidine-susceptible strain of *Serratia* carrying the IncHI2 plasmid was found in other patients, there is no evidence to suggest that this replicon cannot be acquired by such a strain, resulting in multiple antimicrobial resistances. The isolation of a chlorhexidine-tolerant strain devoid of the IncHI2 plasmid (CM2017_569) from a dog indicates that chlorhexidine-tolerant strains can emerge independently of the plasmid acquisition. Interestingly, the phylogenetically divergent *S*. *marcescens* strains CM2017-569, CM2012_028, CM2019_254 and *S*. *ureilytica* strain CM2016_324 possessed largely co-linear chromosomes, similar in organisation to the isolate MSB1_9C (RefSeq accession NZ_LR890657) found in an Australian hospital environment. By contrast, the strain CM2015_854, which is phylogenetically close to CM2017-569 and MSB1_9C, had a similar but somewhat distinct chromosomal organisation, likely caused by several genomic rearrangements. The nine clonal isolates represented by CM2015_854 also carried the quinolone resistance mutation Ser-83-Ile in the GyrA protein sequence. Resistance to fluoroquinolones through well-defined chromosomal QRDRs [55] has been reported in relatively few clinical isolates of *S*. *marcescens* [56,57]. The GyrA mutation reported here is relatively rare in these genomes, and it has been found so far only in human isolates from China and the USA, as well as in MSB1_9C. Taken together, these observations could suggest that a *Serratia* strain present in the veterinary hospital environment had independently acquired the *mexCD-oprJ* operon from an ICE, a MDR IncHI2 plasmid from an *Enterobacteriaceae* donor, and a GyrA mutation leading to fluoroquinolone resistance.

A limitation of this study is the relatively low number of isolates examined, which made it difficult to appreciate the diversity of Australian veterinary *Serratia*. Since the 3 isolates recovered from the same cat (#1) were virtually identical to each other, and the 9 MDR isolates represented a clonal population, only 8 distinct genotypes were effectively explored in this study, including 3 *S*. *ureilytica*. Nevertheless, we were able to place these veterinary isolates into several distinct phylogenetic groups, and to match their phylogenetic diversity to different biochemical profiles. A second limitation is that the tolerance to other biocides was not assessed. At the U-Vet hospital, disinfection protocols for patients and for materials involve various biocides, which may include chlorhexidine-gluconate as well as other products, depending on the purpose of the operating procedure. A thorough comparison of the efficacy of the different biocides commonly used in our premises (e.g. ethanol, detergents, QACs, oxidising agents) as well as other disinfection methods (e.g. conventional or steam cleaning), separately or in combination, would be required to gain a meaningful understanding of the risks of healthcare-associated infections caused by *Serratia* spp. and similar organisms in veterinary settings.

## Conclusions

This study illustrates the potential of *Serratia* spp. to survive in the environment of veterinary hospitals, contaminate disinfectant or antiseptic solutions, and acquire multiple drug resistances through diverse mobile genetic elements. As a consequence, *Serratia* spp. can cause serious opportunistic infections in animals and hinder routine hygiene and disinfection procedures, as well as successful antimicrobial therapy. Routine active and passive surveillance programs are essential tools to detect and control this pathogen in veterinary settings.

## Supporting information

S1 Fig Phylogenetic diversity and plasmid distribution in *S*. *marcescens* genomes. A maximum likelihood tree was generated from concatenated multiple alignments with MEGA using the General Time Reversible model with discrete Gamma distribution and Invariable sites (GTR+G+I), selected by the lowest Bayesian Information Criterion (BIC) score. Top: entire tree derived from the analysis of 671 complete and partial genomes from NCBI. Bottom: close-up showing the position of 9 MDR isolates in the tree. The presence and incompatibility groups of plasmids were predicted by ABRicate with the database plasmidfinder. The presence of the ARGs *mcr*-9.1 and *bla*SHV-12 was predicted by ABRicate with the database resfinder. The unrooted tree was decorated with iTOL.
(TIFF)

S2 Fig Individual growth curves of *Serratia* isolates. The chlorhexidine-susceptible (top panel) and chlorhexidine-resistant (bottom panel) isolates were exposed to water (upper row plots) or disinfectant (lower row plots) for 5 minutes, followed by neutralisation-dilution. Data points from individual ODs measured during a 8 hour time-course experiment for each strain were plotted with the R package ‘Growthcurver’.
(TIFF)

S3 Fig Production of ESBLs by a *Serratia* transconjugant. Disc diffusion assay showing the antimicrobial resistance phenotypes of the donor strain *E*. *hormachei* CM2018_216 (left) the recipient strain *S*. *marcescens* CM2017_569 (middle) and the *Serratia* transconjugant T6 (right). The production of ESBL is indicated by a reduced zone of inhibition and/or a “keyhole effect” between the cephalosporin discs Cefotaxime (CTX), Ceftazidime (CAZ) or Cefpodoxime (CPD) placed on the left side of the plate and the Clavulanate disc (AMC) placed in the middle. The discs placed on the right side of the plate contain Gentamicin (CN), Chloramphenicol (C) and Sulfamethoxazole/Trimethoprim (SXT).
(TIFF)

S1 Table NCBI complete genomes of Serratia spp. used for the phylogenetic analysis. (DOCX)

S2 Table NCBI partial genomes of *S*. *marcescens* isolated from animals. (DOCX)

S3 Table NCBI partial and complete *S*. *marcescens* genomes used for the extended phylogenetic and plasmid analysis. (DOCX)

S4 Table Genes present in the chlorhexidine-tolerant strains CM2015_854 and CM2017_569 but absent from the chlorhexidine-susceptible strains. (DOCX)

## References

[1] Saralegui C, Ponce-Alonso M, Pérez-Viso B, Moles Alegre L, Escribano E, Lázaro-Perona F, et al. Genomics of Serratia marcescens Isolates Causing Outbreaks in the Same Pediatric Unit 47 Years Apart: Position in an Updated Phylogeny of the Species. Front Microbiol. 2020;11(451):1–15. doi: 10.3389/fmicb.2020.00451 32296400 PMC7136904

[2] Marrie TJ, Costerton JW. Prolonged survival of Serratia marcescens in chlorhexidine. Appl Environ Microbiol. 1981;42(6):1093–102. doi: 10.1128/aem.42.6.1093-1102.1981 7032422 PMC244159

[3] Weber DJ, Rutala WA, Sickbert-Bennett EE. Outbreaks associated with contaminated antiseptics and disinfectants. Antimicrob Agents Chemother. 2007;51(12):4217–24. doi: 10.1128/AAC.00138-07 17908945 PMC2167968

[4] de Frutos M, López-Urrutia L, Domínguez-Gil M, Arias M, Muñoz-Bellido JL, Eiros JM, et al. Brote de Serratia marcescens producido por clorhexidina acuosa al 2% contaminada. Enferm Infecc Microbiol Clin. 2017;35(10):624–9. doi: 10.1016/j.eimc.2016.06.016 27495382

[5] Vigeant P, Loo VG, Bertrand C, Dixon C, Hollis R, Pfaller MA, et al. An outbreak of Serratia marcescens infections related to contaminated chlorhexidine. Infect Control Hosp Epidemiol. 1998;19(10):791–4. doi: 10.1086/647728 9801292

[6] Keck N, Dunie-merigot A, Dazas M, Hirchaud E, Laurence S, Gervais B, et al. Long-lasting nosocomial persistence of chlorhexidine-resistant Serratia marcescens in a veterinary hospital. Vet Microbiol. 2020;245:108686. doi: 10.1016/j.vetmic.2020.108686 32456825

[7] Mahlen SD. Serratia infections: from military experiments to current practice. Clin Microbiol Rev. 2011;24(4):755–91. doi: 10.1128/CMR.00017-11 21976608 PMC3194826

[8] Grimont PA, Grimont F. Biotyping of Serratia marcescens and its use in epidemiological studies. J Clin Microbiol. 1978;8(1):73–83. doi: 10.1128/jcm.8.1.73-83.1978 353073 PMC275117

[9] Bourdin T, Monnier A, Benoit ME, Bedard E, Prevost M, Quach C, et al. A High-Throughput Short Sequence Typing Scheme for Serratia marcescens Pure Culture and Environmental DNA. Appl Environ Microbiol. 2021;87(24):e0139921. doi: 10.1128/AEM.01399-21 34586910 PMC8612290

[10] Abreo E, Altier N. Pangenome of Serratia marcescens strains from nosocomial and environmental origins reveals different populations and the links between them. Sci Rep. 2019;9(1):46. doi: 10.1038/s41598-018-37118-0 30631083 PMC6328595

[11] Garratt I, Aranega-Bou P, Sutton JM, Moore G, Wand ME. Long-Term Exposure to Octenidine in a Simulated Sink Trap Environment Results in Selection of Pseudomonas aeruginosa, Citrobacter, and Enterobacter Isolates with Mutations in Efflux Pump Regulators. Appl Environ Microbiol. 2021;87(10):e00210–21. doi: 10.1128/AEM.00210-21 33674437 PMC8117763

[12] Weingarten RA, Johnson RC, Conlan S, Ramsburg AM, Dekker JP, Lau AF, et al. Genomic Analysis of Hospital Plumbing Reveals Diverse Reservoir of Bacterial Plasmids Conferring Carbapenem Resistance. mBio. 2018;9(1):e02011–17. doi: 10.1128/mBio.02011-17 29437920 PMC5801463

[13] Kamathewatta K, Bushell R, Rafa F, Browning G, Billman-Jacobe H, Marenda M. Colonization of a hand washing sink in a veterinary hospital by an Enterobacter hormaechei strain carrying multiple resistances to high importance antimicrobials. Antimicrob Resist Infect Control. 2020;9(1):163. doi: 10.1186/s13756-020-00828-0 33087168 PMC7580002

[14] Bell SM. The CDS disc method of antibiotic sensitivity testing (calibrated dichotomous sensitivity test). Pathology. 1975;7(4 Suppl):Suppl 1–48. doi: 10.3109/00313027509082602 772573

[15] Krueger F. Trim Galore; [cited 2021]. Database: Github [Internet]. Available from: https://github.com/FelixKrueger/TrimGalore.

[16] Wick RR. Filtlong; [cited 2021]. Database: Github [Internet]. Available from: https://github.com/rrwick/Filtlong.

[17] Kolmogorov M, Yuan J, Lin Y, Pevzner PA. Assembly of long, error-prone reads using repeat graphs. Nat Biotechnol. 2019;37(5):540–6. doi: 10.1038/s41587-019-0072-8 30936562

[18] Wick RR, Judd LM, Gorrie CL, Holt KE. Unicycler: Resolving bacterial genome assemblies from short and long sequencing reads. PLoS Comput Biol. 2017;13(6):e1005595. doi: 10.1371/journal.pcbi.1005595 28594827 PMC5481147

[19] Seemann T. Prokka: rapid prokaryotic genome annotation. Bioinformatics. 2014;30(14):2068–9. doi: 10.1093/bioinformatics/btu153 24642063

[20] Jain C, Rodriguez-R LM, Phillippy AM, Konstantinidis KT, Aluru S. High throughput ANI analysis of 90K prokaryotic genomes reveals clear species boundaries. Nat Commun. 2018;9(1):5114. doi: 10.1038/s41467-018-07641-9 30504855 PMC6269478

[21] Zhao S, Guo Y, Sheng Q, Shyr Y. Heatmap3: an improved heatmap package with more powerful and convenient features. BMC Bioinformatics. 2014;15(10):P16. doi: 10.1186/1471-2105-15-S10-P16

[22] Treangen TJ, Ondov BD, Koren S, Phillippy AM. The Harvest suite for rapid core-genome alignment and visualization of thousands of intraspecific microbial genomes. Genome Biology. 2014;15(11):524. doi: 10.1186/s13059-014-0524-x 25410596 PMC4262987

[23] Seemann T. Rapid haploid variant calling and core genome alignment; [cited 2021]. Database: Github [Internet]. Available from: https://github.com/tseemann/snippy.

[24] Edgar RC. MUSCLE: multiple sequence alignment with high accuracy and high throughput. Nucleic Acids Res. 2004;32(5):1792–7. doi: 10.1093/nar/gkh340 15034147 PMC390337

[25] Kumar S, Stecher G, Li M, Knyaz C, Tamura K. MEGA X: Molecular Evolutionary Genetics Analysis across Computing Platforms. Mol Biol Evol. 2018;35(6):1547–9. doi: 10.1093/molbev/msy096 29722887 PMC5967553

[26] Letunic I, Bork P. Interactive Tree Of Life (iTOL) v5: an online tool for phylogenetic tree display and annotation. Nucleic Acids Res. 2021;49(W1):W293–W296. doi: 10.1093/nar/gkab301 33885785 PMC8265157

[27] Seemann T. ABRicate, Mass screening of contigs for antimicrobial resistance or virulence genes; [cited 2021]. Database: Github [Internet]. Available from: https://github.com/tseemann/abricate.

[28] Anonymous. Importance Ratings and Summary of Antibacterial Uses in Humans in Australia; 2015. Database. Available from: https://www.amr.gov.au/resources/importance-ratings-and-summary-antibacterial-uses-humans-australia.

[29] Galata V, Fehlmann T, Backes C, Keller A. PLSDB: a resource of complete bacterial plasmids. Nucleic Acids Res. 2019;47(D1):D195–d202. doi: 10.1093/nar/gky1050 30380090 PMC6323999

[30] Alikhan N-F, Petty NK, Ben Zakour NL, Beatson SA. BLAST Ring Image Generator (BRIG): simple prokaryote genome comparisons. BMC Genomics. 2011;12(1):402. doi: 10.1186/1471-2164-12-402 21824423 PMC3163573

[31] Darling AE, Mau B, Perna NT. progressiveMauve: multiple genome alignment with gene gain, loss and rearrangement. PLoS One. 2010;5(6):e11147. doi: 10.1371/journal.pone.0011147 20593022 PMC2892488

[32] Gilchrist CLM, Chooi Y-H. clinker & clustermap.js: automatic generation of gene cluster comparison figures. Bioinformatics. 2021;37(16): 2473–75. doi: 10.1093/bioinformatics/btab007 33459763

[33] Arndt D, Grant JR, Marcu A, Sajed T, Pon A, Liang Y, et al. PHASTER: a better, faster version of the PHAST phage search tool. Nucleic Acids Res. 2016;44(W1):W16–21. doi: 10.1093/nar/gkw387 27141966 PMC4987931

[34] Bertelli C, Laird MR, Williams KP, Simon Fraser University Research Computing Group, Lau BY, Hoad G, et al. IslandViewer 4: expanded prediction of genomic islands for larger-scale datasets. Nucleic Acids Res. 2017;45(W1):W30–W5. doi: 10.1093/nar/gkx343 28472413 PMC5570257

[35] Liu M, Li X, Xie Y, Bi D, Sun J, Li J, et al. ICEberg 2.0: an updated database of bacterial integrative and conjugative elements. Nucleic Acids Res. 2019;47(D1):D660–D5. doi: 10.1093/nar/gky1123 30407568 PMC6323972

[36] Page AJ, Cummins CA, Hunt M, Wong VK, Reuter S, Holden MT, et al. Roary: rapid large-scale prokaryote pan genome analysis. Bioinformatics. 2015;31(22):3691–3. doi: 10.1093/bioinformatics/btv421 26198102 PMC4817141

[37] Aramaki T, Blanc-Mathieu R, Endo H, Ohkubo K, Kanehisa M, Goto S, et al. KofamKOALA: KEGG Ortholog assignment based on profile HMM and adaptive score threshold. Bioinformatics. 2020;36(7):2251–2. doi: 10.1093/bioinformatics/btz859 31742321 PMC7141845

[38] Kanehisa M, Goto S. KEGG: kyoto encyclopedia of genes and genomes. Nucleic Acids Res. 2000;28(1):27–30. doi: 10.1093/nar/28.1.27 10592173 PMC102409

[39] Pal C, Bengtsson-Palme J, Rensing C, Kristiansson E, Larsson DG. BacMet: antibacterial biocide and metal resistance genes database. Nucleic Acids Res. 2014;42(Database issue):D737–43. doi: 10.1093/nar/gkt1252 24304895 PMC3965030

[40] Sprouffske K, Wagner A. Growthcurver: an R package for obtaining interpretable metrics from microbial growth curves. BMC Bioinformatics. 2016;17(1):172. doi: 10.1186/s12859-016-1016-7 27094401 PMC4837600

[41] Jarlier V, Nicolas MH, Fournier G, Philippon A. Extended broad-spectrum beta-lactamases conferring transferable resistance to newer beta-lactam agents in Enterobacteriaceae: hospital prevalence and susceptibility patterns. Rev Infect Dis. 1988;10(4):867–78. doi: 10.1093/clinids/10.4.867 3263690

[42] Yoon HJ, Choi JY, Park YS, Kim CO, Kim JM, Yong DE, et al. Outbreaks of Serratia marcescens bacteriuria in a neurosurgical intensive care unit of a tertiary care teaching hospital: a clinical, epidemiologic, and laboratory perspective. Am J Infect Control. 2005;33(10):595–601. doi: 10.1016/j.ajic.2005.01.010 16330308

[43] Johnson J, Quach C. Outbreaks in the neonatal ICU: a review of the literature. Curr Opin Infect Dis. 2017;30(4):395–403. doi: 10.1097/QCO.0000000000000383 28582313 PMC8020806

[44] Bhadra B, Roy P, Chakraborty R. Serratia ureilytica sp. nov., a novel urea-utilizing species. Int J Syst Evol Microbiol. 2005;55(Pt 5):2155–8. doi: 10.1099/ijs.0.63674-0 16166724

[45] Gilmour MW, Thomson NR, Sanders M, Parkhill J, Taylor DE. The complete nucleotide sequence of the resistance plasmid R478: defining the backbone components of incompatibility group H conjugative plasmids through comparative genomics. Plasmid. 2004;52(3):182–202. doi: 10.1016/j.plasmid.2004.06.006 15518875

[46] Shahcheraghi F, Minato Y, Chen J, Mizushima T, Ogawa W, Kuroda T, et al. Molecular cloning and characterization of a multidrug efflux pump, SmfY, from Serratia marcescens. Biol Pharm Bull. 2007;30(4):798–800. doi: 10.1248/bpb.30.798 17409524

[47] Matsuo T, Chen J, Minato Y, Ogawa W, Mizushima T, Kuroda T, et al. SmdAB, a heterodimeric ABC-Type multidrug efflux pump, in Serratia marcescens. J Bacteriol. 2008;190(2):648–54. doi: 10.1128/JB.01513-07 18024518 PMC2223691

[48] Anes J, McCusker MP, Fanning S, Martins M. The ins and outs of RND efflux pumps in Escherichia coli. Front Microbiol. 2015;6:587. doi: 10.3389/fmicb.2015.00587 26113845 PMC4462101

[49] Kumar A, Worobec EA. Cloning, sequencing, and characterization of the SdeAB multidrug efflux pump of Serratia marcescens. Antimicrob Agents Chemother. 2005;49(4):1495–501. doi: 10.1128/AAC.49.4.1495-1501.2005 15793131 PMC1068610

[50] Begic S, Worobec EA. Characterization of the Serratia marcescens SdeCDE multidrug efflux pump studied via gene knockout mutagenesis. Can J Microbiol. 2008;54(5):411–6. doi: 10.1139/w08-019 18449226

[51] Chen J, Kuroda T, Huda MN, Mizushima T, Tsuchiya T. An RND-type multidrug efflux pump SdeXY from Serratia marcescens. J Antimicrob Chemother. 2003;52(2):176–9. doi: 10.1093/jac/dkg308 12837741

[52] Begic S, Worobec EA. The role of the Serratia marcescens SdeAB multidrug efflux pump and TolC homologue in fluoroquinolone resistance studied via gene-knockout mutagenesis. Microbiology. 2008;154(2):454–61. doi: 10.1099/mic.0.2007/012427-0 18227249

[53] Rajamohan G, Srinivasan VB, Gebreyes WA. Molecular and functional characterization of a novel efflux pump, AmvA, mediating antimicrobial and disinfectant resistance in Acinetobacter baumannii. J Antimicrob Chemother. 2010;65(9):1919–25. doi: 10.1093/jac/dkq195 20573661

[54] Fraud S, Campigotto AJ, Chen Z, Poole K. MexCD-OprJ multidrug efflux system of Pseudomonas aeruginosa: involvement in chlorhexidine resistance and induction by membrane-damaging agents dependent upon the AlgU stress response sigma factor. Antimicrob Agents Chemother. 2008;52(12):4478–82. doi: 10.1128/AAC.01072-08 18838593 PMC2592872

[55] Drlica K, Zhao X. DNA gyrase, topoisomerase IV, and the 4-quinolones. Microbiol Mol Biol Rev. 1997;61(3):377–92. doi: 10.1128/mmbr.61.3.377-392.1997 9293187 PMC232616

[56] Kim JH, Cho EH, Kim KS, Kim HY, Kim YM. Cloning and nucleotide sequence of the DNA gyrase gyrA gene from Serratia marcescens and characterization of mutations in gyrA of quinolone-resistant clinical isolates. Antimicrob Agents Chemother. 1998;42(1):190–3. doi: 10.1128/AAC.42.1.190 9449286 PMC105481

[57] Weigel LM, Steward CD, Tenover FC. gyrA mutations associated with fluoroquinolone resistance in eight species of Enterobacteriaceae. Antimicrob Agents Chemother. 1998;42(10):2661–7. doi: 10.1128/AAC.42.10.2661 9756773 PMC105915

