# Supplementary figures and images for: Healthcare-associated infections caused by chlorhexidine-tolerant Serratia marcescens carrying a promiscuous IncHI2 multi-drug resistance plasmid in a veterinary hospital
Source: PLoS One. 2022 Mar 17;17(3):e0264848. doi: 10.1371/journal.pone.0264848 (PMC8929579; doi:10.1371/journal.pone.0264848)

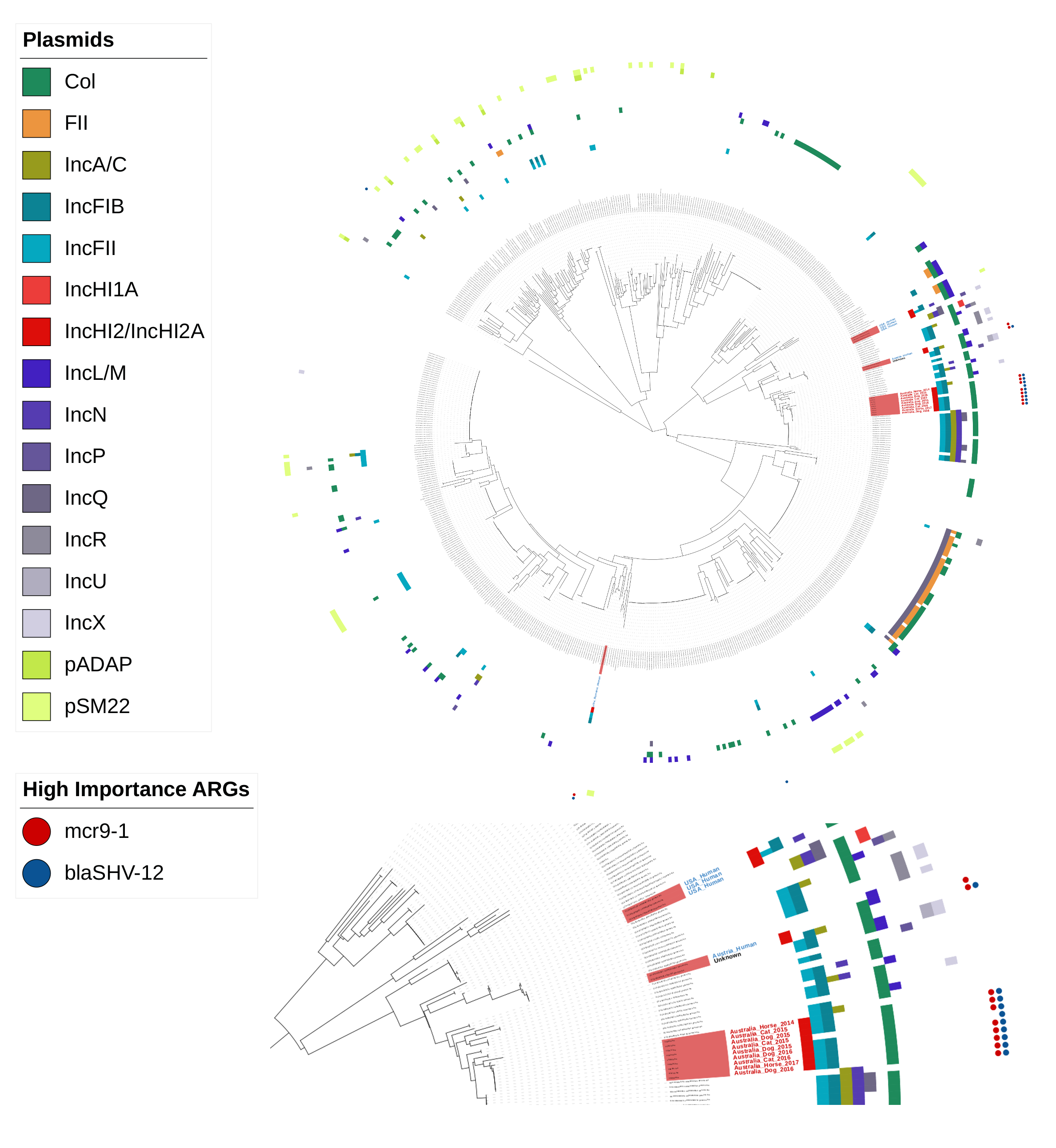

Supplement: S1 Fig — A maximum likelihood tree was generated from concatenated multiple alignments with MEGA using the General Time Reversible model with discrete Gamma distribution and Invariable sites (GTR+G+I), selected by the lowest Bayesian Information Criterion (BIC) score. Top: entire tree derived from the analysis of 671 complete and partial genomes from NCBI. Bottom: close-up showing the position of 9 MDR isolates in the tree. The presence and incompatibility groups of plasmids were predicted by ABRicate with the database plasmidfinder. The presence of the ARGs mcr-9.1 and blaSHV-12 was predicted by ABRicate with the database resfinder. The unrooted tree was decorated with iTOL. (TIFF) [file pone.0264848.s001.tiff]

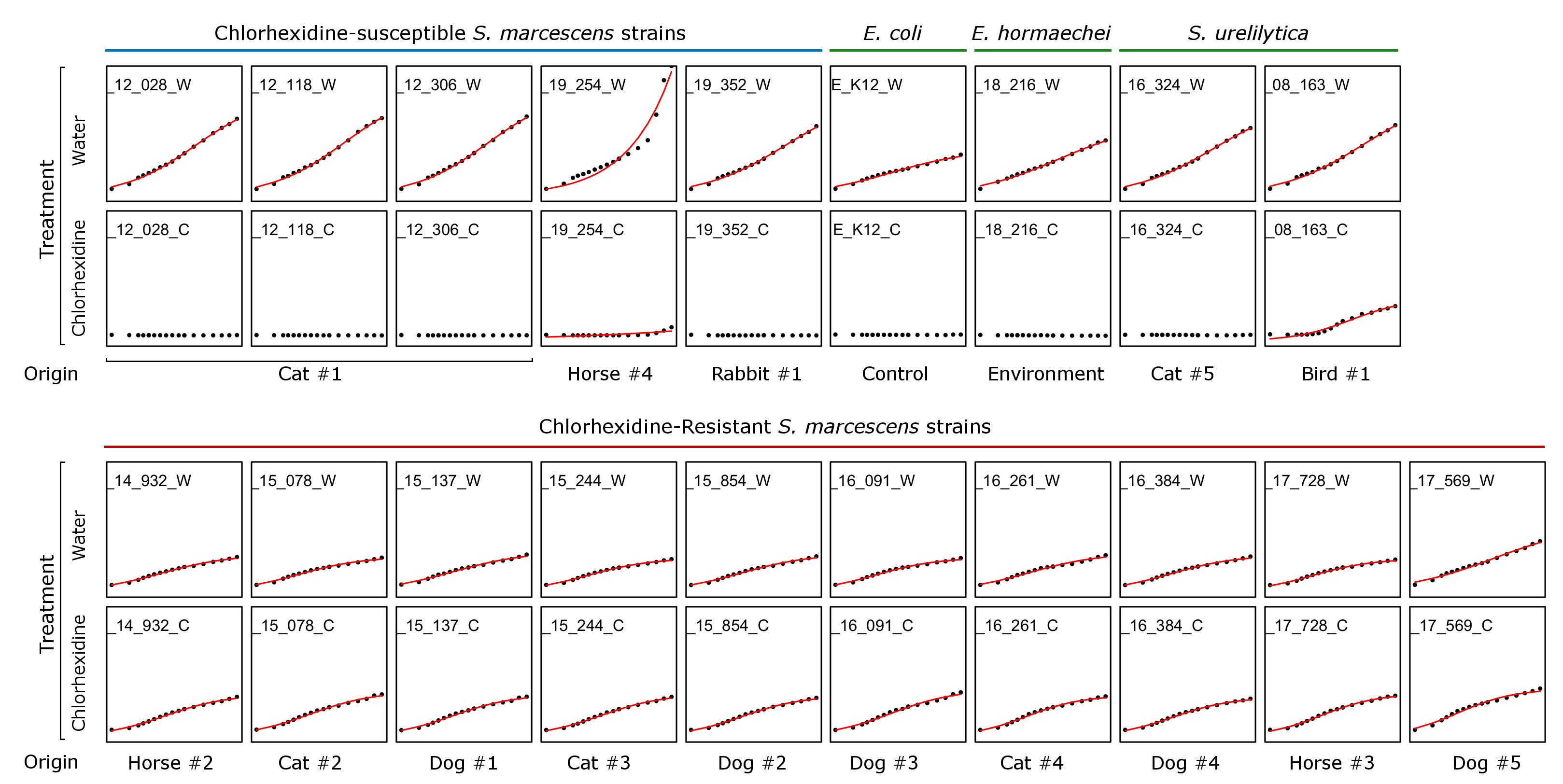

Supplement: S2 Fig — The chlorhexidine-susceptible (top panel) and chlorhexidine-resistant (bottom panel) isolates were exposed to water (upper row plots) or disinfectant (lower row plots) for 5 minutes, followed by neutralisation-dilution. Data points from individual ODs measured during a 8 hour time-course experiment for each strain were plotted with the R package ‘Growthcurver’. (TIFF) [file pone.0264848.s002.tiff]

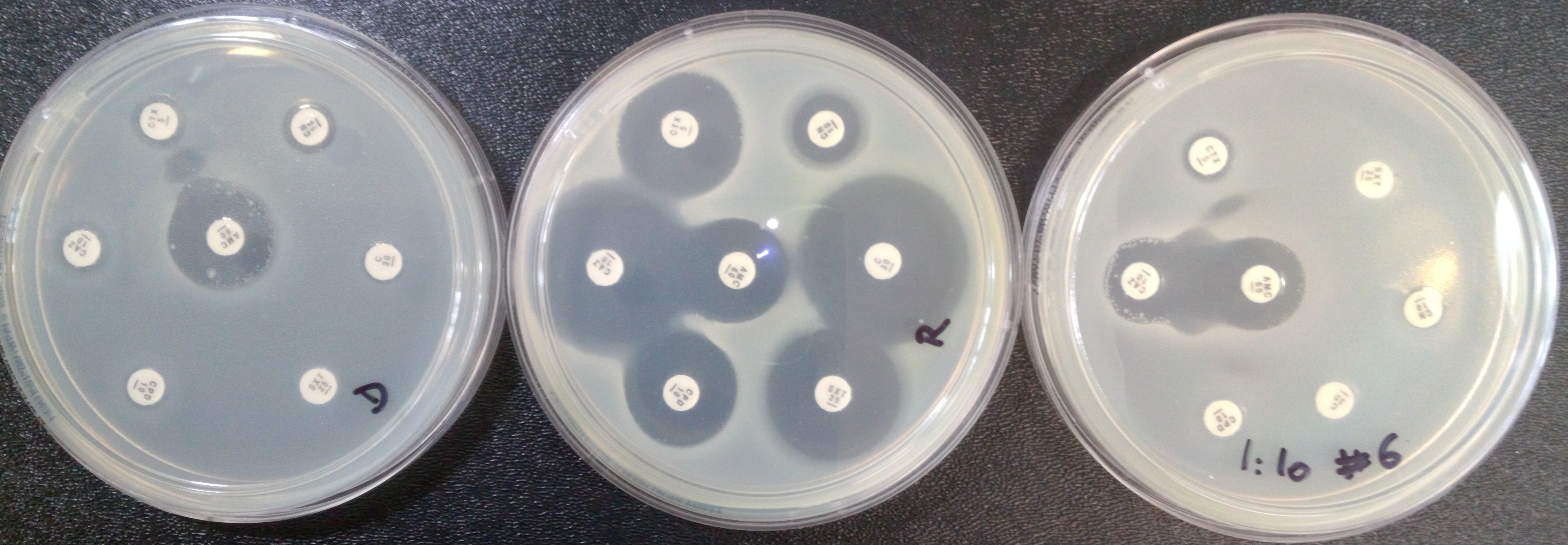

Supplement: S3 Fig — Disc diffusion assay showing the antimicrobial resistance phenotypes of the donor strain E. hormachei CM2018_216 (left) the recipient strain S. marcescens CM2017_569 (middle) and the Serratia transconjugant T6 (right). The production of ESBL is indicated by a reduced zone of inhibition and/or a “keyhole effect” between the cephalosporin discs Cefotaxime (CTX), Ceftazidime (CAZ) or Cefpodoxime (CPD) placed on the left side of the plate and the Clavulanate disc (AMC) placed in the middle. The discs placed on the right side of the plate contain Gentamicin (CN), Chloramphenicol (C) and Sulfamethoxazole/Trimethoprim (SXT). (TIFF) [file pone.0264848.s003.tiff]
