# Supplementary material for: Healthcare-associated infections caused by chlorhexidine-tolerant Serratia marcescens carrying a promiscuous IncHI2 multi-drug resistance plasmid in a veterinary hospital
Source: PLoS One. 2022 Mar 17;17(3):e0264848. doi: 10.1371/journal.pone.0264848 (PMC8929579; doi:10.1371/journal.pone.0264848)
Supplement: S1 Table — (DOCX) [file pone.0264848.s004.docx]

Table S1. NCBI complete genomes of *Serratia* spp. used for the phylogenetic analysis

| Genbank_Accession | Organism | Strain | Host | Isolation_source | Country | Year |
| --- | --- | --- | --- | --- | --- | --- |
| GCA_900187015.1_50465_F01 | Serratia_ficaria | NCTC12148 | - | - | - | 1900/1981 |
| GCA_001006005.1_ASM100600v1 | Serratia_fonticola | DSM_4576 | - | water | - | 1979 |
| GCA_001514455.1_ASM151445v1 | Serratia_fonticola | GS2 | Sesame | soil | South_Korea | 2015 |
| GCA_002588845.1_ASM258884v1 | Serratia_fonticola | FDAARGOS_411 | Wildlife | Liver_tissue | - | - |
| GCA_005489985.1_ASM548998v1 | Serratia_fonticola | MS5 | Aedes_aegypti | - | USA_Las_Cruces | 2017 |
| GCA_011691375.1_ASM1169137v1 | Serratia_fonticola | CPSE11 | - | root_of_Codonopsis_pilosula | China_Gansu | 2019 |
| GCA_013284015.1_ASM1328401v1 | Serratia_fonticola | HH13 | - | - | - | 2009 |
| GCA_900638145.1_56433_F01 | Serratia_fonticola | NCTC13193 | - | not_available_to_be_reported_later | - | - |
| GCA_000975245.1_ASM97524v1 | Serratia_liquefaciens | HUMV_21 | Homo_sapiens | skin_ulcer | Spain_Santander | 2009 |
| GCA_001559135.2_ASM155913v2 | Serratia_liquefaciens | FDAARGOS_125 | Homo_sapiens | - | USA_DC | 2013 |
| GCA_003074975.2_ASM307497v2 | Serratia_liquefaciens | JL02 | cattle | milk_from_cattle_farm | China_Jilin | 2017 |
| GCA_006970665.1_ASM697066v1 | Serratia_liquefaciens | FG3 | Stachytarpheta_glabra | - | Brazil_Minas_Gerais | 2016 |
| GCA_008364325.2_ASM836432v2 | Serratia_liquefaciens | S1 | - | Mixed_salads | Germany | 2015 |
| GCA_014495865.1_ASM1449586v1 | Serratia_liquefaciens | MT49 | - | groundwater_from_well | USA_Oak_Ridge_Tennessee | 2013 |
| GCA_016728005.1_ASM1672800v1 | Serratia_liquefaciens | FDAARGOS_1081 | - | - | Germany_Langen | - |
| GCA_000422085.1_ASM42208v1 | Serratia_liquefaciens | ATCC_27592 | - | - | - | - |
| GCA_000783915.2_ASM78391v2 | Serratia_marcescens | FDAARGOS_65 | Homo_sapiens | Endotracheal_aspirate | USA | 2013 |
| GCA_001022215.1_ASM102221v1 | Serratia_marcescens | CAV1492 | Homo_sapiens | Respiratory | USA_Virginia | 2011 |
| GCA_001280365.1_ASM128036v1 | Serratia_marcescens | RSC_14 | Solanum_nigrum | - | South_Korea | 2013 |
| GCA_001294565.1_ASM129456v1 | Serratia_marcescens | SmUNAM836 | Homo_sapiens | bronchial_aspirate | Mexico_Distrito_Federal | 2005 |
| GCA_001417865.2_ASM141786v2 | Serratia_marcescens | B3R3 | Zea_mays | - | China_Shandong | 2011 |
| GCA_001672055.1_ASM167205v1 | Serratia_marcescens | U36365 | Homo_sapiens | Urine | India_Vellore_TN | 2015 |
| GCA_002220515.1_ASM222051v1 | Serratia_marcescens | UMH2 | Homo_sapiens | University_of_Michigan_Health_System | USA_Michigan | 2014 |
| GCA_002220535.1_ASM222053v1 | Serratia_marcescens | UMH8 | Homo_sapiens | University_of_Michigan_Health_System | USA_Michigan | 2013 |
| GCA_002220555.1_ASM222055v1 | Serratia_marcescens | UMH9 | Homo_sapiens | - | USA_Michigan | 2014 |
| GCA_002220575.1_ASM222057v1 | Serratia_marcescens | UMH11 | Homo_sapiens | University_of_Michigan_Health_System | USA_Michigan | 2014 |
| GCA_002220595.1_ASM222059v1 | Serratia_marcescens | UMH12 | Homo_sapiens | University_of_Michigan_Health_System | USA_Michigan | 2014 |
| GCA_002220615.1_ASM222061v1 | Serratia_marcescens | UMH1 | Homo_sapiens | - | USA_Michigan | 2013 |
| GCA_002220635.1_ASM222063v1 | Serratia_marcescens | UMH5 | Homo_sapiens | - | USA_Michigan | 2014 |
| GCA_002220655.1_ASM222065v1 | Serratia_marcescens | UMH3 | Homo_sapiens | University_of_Michigan_Health_System | USA_Michigan | 2014 |
| GCA_002220675.1_ASM222067v1 | Serratia_marcescens | UMH6 | Homo_sapiens | University_of_Michigan_Health_System | USA_Michigan | 2013 |
| GCA_002220695.1_ASM222069v1 | Serratia_marcescens | UMH10 | Homo_sapiens | University_of_Michigan_Health_System | USA_Michigan | 2014 |
| GCA_002220715.1_ASM222071v1 | Serratia_marcescens | UMH7 | Homo_sapiens | - | USA_Michigan | 2013 |
| GCA_002947235.1_ASM294723v1 | Serratia_marcescens | AR_0027 | - | - | - | - |
| GCA_002996885.1_ASM299688v1 | Serratia_marcescens | AR_0091 | - | - | - | - |
| GCA_002997125.1_ASM299712v1 | Serratia_marcescens | AR_0099 | - | - | - | - |
| GCA_003031545.1_ASM303154v1 | Serratia_marcescens | 95 | Homo_sapiens | sputum | USA_Boston | 2015 |
| GCA_003031645.1_ASM303164v1 | Serratia_marcescens | BWH_35 | Homo_sapiens | sputum | USA_Boston | 2012 |
| GCA_003071565.1_ASM307156v1 | Serratia_marcescens | AR_0124 | - | - | - | - |
| GCA_003071585.1_ASM307158v1 | Serratia_marcescens | AR_0130 | - | - | - | - |
| GCA_003071605.1_ASM307160v1 | Serratia_marcescens | AR_0123 | - | - | - | - |
| GCA_003071625.1_ASM307162v1 | Serratia_marcescens | AR_0121 | - | - | - | - |
| GCA_003146705.1_ASM314670v1 | Serratia_marcescens | CAV1761 | Homo_sapiens | Peri-rectal | USA_Virginia | 2014 |
| GCA_003182655.1_ASM318265v1 | Serratia_marcescens | SGAir0764 | - | air | Singapore | 2016 |
| GCA_003186475.1_ASM318647v1 | Serratia_marcescens | 332 | Homo_sapiens | Wound | USA_Boston | 2016 |
| GCA_003204075.1_ASM320407v1 | Serratia_marcescens | AR_0131 | - | - | - | - |
| GCA_003204405.1_ASM320440v1 | Serratia_marcescens | AR_0122 | - | - | - | - |
| GCA_003355135.1_ASM335513v1 | Serratia_marcescens | N4_5 | - | soil | USA_New_Jersey | 1995 |
| GCA_003967055.1_ASM396705v1 | Serratia_marcescens | AS_1 | - | soil | Japan_Tochigi | - |
| GCA_006711125.1_ASM671112v1 | Serratia_marcescens | WVU_004 | Homo_sapiens | blood | USA_Morgantown | 2019 |
| GCA_006711145.1_ASM671114v1 | Serratia_marcescens | WVU_005 | Homo_sapiens | blood | USA_Morgantown | 2019 |
| GCA_006711245.1_ASM671124v1 | Serratia_marcescens | WVU_006 | Homo_sapiens | blood | USA_Morgantown | 2019 |
| GCA_006711405.1_ASM671140v1 | Serratia_marcescens | WVU_007 | Homo_sapiens | blood | USA_Morgantown | 2019 |
| GCA_006711525.1_ASM671152v1 | Serratia_marcescens | WVU_008 | Homo_sapiens | blood | USA_Morgantown | 2019 |
| GCA_006716725.1_ASM671672v1 | Serratia_marcescens | WVU_009 | Homo_sapiens | blood | USA_Morgantown | 2019 |
| GCA_006716825.1_ASM671682v1 | Serratia_marcescens | WVU_010 | Homo_sapiens | blood | USA_Morgantown | 2019 |
| GCA_006842785.1_ASM684278v1 | Serratia_marcescens | WVU_002 | Homo_sapiens | blood | USA_Morgantown | 2018 |
| GCA_008364265.2_ASM836426v2 | Serratia_marcescens | S7_1 | - | Mixed_salads | Germany | 2015 |
| GCA_008931425.1_ASM893142v1 | Serratia_marcescens | E28 | - | Ensuite_7/8 | Australia_Sydney | 2012 |
| GCA_009834305.1_ASM983430v1 | Serratia_marcescens | N10A28 | Apis_mellifera | - | USA_Connecticut | 2011 |
| GCA_009858195.1_ASM985819v1 | Serratia_marcescens | 1602 | Homo_sapiens | - | China_Zhengzhou | 2018 |
| GCA_009909345.1_ASM990934v1 | Serratia_marcescens | 4201 | Homo_sapiens | sputum | China | 2019 |
| GCA_009909365.1_ASM990936v1 | Serratia_marcescens | 3024 | Homo_sapiens | blood | China | 2018 |
| GCA_009909385.1_ASM990938v1 | Serratia_marcescens | 1140_ | Homo_sapiens | body_fluid | China | 2018 |
| GCA_009909405.1_ASM990940v1 | Serratia_marcescens | 2838 | Homo_sapiens | body_fluid | China | 2018 |
| GCA_009909425.1_ASM990942v1 | Serratia_marcescens | C110 | Homo_sapiens | sputum | China | 2018 |
| GCA_009936295.1_ASM993629v1 | Serratia_marcescens | ATCC_274 | - | - | - | - |
| **GCA_011602465.1_ASM1160246v1** | Serratia_marcescens | BP2 | Jatropha_curcas | seeds | Brazil | 2010 |
| GCA_011769885.1_ASM1176988v1 | Serratia_marcescens | SER00094 | Homo_sapiens | sputum | USA_Pennsylvania | 2017 |
| GCA_013112395.1_ASM1311239v1 | Serratia_marcescens | FZSF02 | - | soil | China_Fuzhou | 2014 |
| GCA_013122155.1_ASM1312215v1 | Serratia_marcescens | FY | Drosophila_melanogaster | - | China_Shanxi | 2016 |
| GCA_013256815.1_ASM1325681v1 | Serratia_marcescens | LY1 | insect | - | China_muchuan | 2019 |
| GCA_013367735.1_ASM1336773v1 | Serratia_marcescens | JW_CZ2 | - | rhizosphere_soil_of_tea_tree | China_anhui | 2014 |
| GCA_013377375.1_ASM1337737v1 | Serratia_marcescens | 1912768R | - | rhizosphere_soil_in_ginger_field | China_Sichuan | 2015 |
| GCA_013426135.1_ASM1342613v1 | Serratia_marcescens | 12_2010 | Homo_sapiens | platelet_concentrate | Canada_Ottawa | 2010 |
| GCA_013426155.1_ASM1342615v1 | Serratia_marcescens | 11_2010 | Homo_sapiens | platelet_concentrate | Canada_Ottawa | 2010 |
| GCA_015074945.1_ASM1507494v1 | Serratia_marcescens | SCH909 | Homo_sapiens | Greece | Greece | 1988 |
| GCA_015160915.1_ASM1516091v1 | Serratia_marcescens | SCQ1 | silkworm | blood_from_silkworm | China_Chongqing | 2009 |
| GCA_015708655.1_ASM1570865v1 | Serratia_marcescens | Byron | Curculio_caryae | pupal_cells | USA_Georgia_Byron | 2014 |
| GCA_904866365.1_MSB1_9C | Serratia_marcescens | MSB1_9C | - | - | - | - |
| GCA_000828775.1_ASM82877v1 | Serratia_marcescens | SM39 | - | - | - | - |
| GCA_900029885.1_Sm_SMB2099 | Serratia_marcescens | SMB2099 | - | clinical_isolate | - | - |
| GCA_006974205.1_ASM697420v1 | Serratia_marcescens | ATCC_13880 | - | pond_water | USA | 1969 |
| GCA_000513215.1_DB11 | Serratia_marcescens | Db11 | - | - | - | - |
| GCA_000336425.1_ASM33642v1 | Serratia_marcescens | WW4 | - | - | - | - |
| GCA_004768745.1_ASM476874v1 | Serratia_nematodiphila | DH_S01 | - | - | - | - |
| GCA_900635445.1_28869_A02 | Serratia_odorifera | NCTC11214 | - | not_available_to_be_reported_later | - | - |
| GCA_001663115.1_ASM166311v1 | Serratia_plymuthica | 3Rp8 | Brassica_napus | organic_material | Germany_Braunschweig | 1998 |
| GCA_001663135.1_ASM166313v1 | Serratia_plymuthica | 3Re4_18 | Solanum_tuberosum_L._cv._Cilena | organic_material | Germany_Bonn | 2001 |
| GCA_013122215.1_ASM1312221v1 | Serratia_plymuthica | C_1 | Capsicum_annuum | Red_pepper_grown_commercial_farm | South_Korea_Naju | 1998 |
| GCA_016027115.1_ASM1602711v1 | Serratia_plymuthica | FDAARGOS_907 | - | - | - | - |
| GCA_016027595.1_ASM1602759v1 | Serratia_plymuthica | FDAARGOS_896 | - | - | - | - |
| GCA_016027675.1_ASM1602767v1 | Serratia_plymuthica | FDAARGOS_895 | - | - | - | - |
| GCA_016027835.1_ASM1602783v1 | Serratia_plymuthica | FDAARGOS_889 | - | - | - | - |
| GCA_016726325.1_ASM1672632v1 | Serratia_plymuthica | FDAARGOS_1138 | - | - | Germany_Braunschweig | - |
| GCA_900478125.1_28193_B02 | Serratia_plymuthica | NCTC12961 | - | - | - | - |
| GCA_900635625.1_31436_B01 | Serratia_plymuthica | NCTC8900 | - | not_available_to_be_reported_later | - | - |
| GCA_900637965.1_53990_D02 | Serratia_plymuthica | NCTC8015 | - | Canal_water | - | - |
| GCA_000176835.2_ASM17683v2 | Serratia_plymuthica | 4Rx13 | - | - | - | - |
| GCA_000214235.1_ASM21423v1 | Serratia_plymuthica | AS9 | - | - | - | - |
| GCA_000261045.2_ASM26104v2 | Serratia_plymuthica | PRI_2c | - | maize_rhizosphere_soil | Netherlands | 2004 |
| GCA_000438825.1_ASM43882v1 | Serratia_plymuthica | S13 | - | Styrian_pumpkin_anthrosphere | - | - |
| GCA_009660185.1_ASM966018v1 | Serratia_proteamaculans | 336X | - | leaf | China | 2017 |
| GCA_000018085.1_ASM1808v1 | Serratia_proteamaculans | 568 | - | - | - | - |
| GCA_004684265.1_ASM468426v1 | Serratia_quinivorans | PKL_12 | - | rhizospheric_soil_of_Picrorrhiza_kurroa | India_Lahaul-Spiti | 2015 |
| GCA_900638135.1_56433_G01 | Serratia_quinivorans | NCTC13188 | - | not_available_to_be_reported_later | - | - |
| GCA_001572725.1_ASM157272v1 | Serratia_rubidaea | 1122 | Homo_sapiens | sputum | China_Beijing | 2014 |
| GCA_016026735.1_ASM1602673v1 | Serratia_rubidaea | FDAARGOS_926 | - | - | - | - |
| GCA_016028475.1_ASM1602847v1 | Serratia_rubidaea | FDAARGOS_880 | - | - | - | - |
| GCA_900478395.1_32135_B01 | Serratia_rubidaea | NCTC10848 | - | - | - | - |
| GCA_900635665.1_31769_F01 | Serratia_rubidaea | NCTC9419 | - | not_available_to_be_reported_later | - | - |
| GCA_900638005.1_53550_B01 | Serratia_rubidaea | NCTC10036 | - | Finger | - | - |
| GCA_901472405.1_28771_E01 | Serratia_rubidaea | NCTC12971 | - | not_available_not_collected | - | - |
| GCA_003668775.1_ASM366877v1 | Serratia_sp. | 3ACOL1 | Cerambycidae_sp. | larvae | Norway | 2017 |
| GCA_009817885.1_ASM981788v1 | Serratia_sp. | KUDC3025 | Artemisia_japonica_subsp._littoricola_Kitam | rhizospheric_soil | South_Korea | 2017 |
| GCA_002966855.1_ASM296685v1 | Serratia_sp. | MYb239 | - | compost | Germany_Kiel | - |
| GCA_905188235.1_ASM90518823v1 | Serratia_sp. | Tan611 | - | activated_sludge | - | - |
| GCA_000330865.1_ASM33086v1 | Serratia_sp. | FGI94 | - | - | - | - |
| GCA_003641105.1_ASM364110v1 | Serratia_sp. | 1D1416 | Euonymus_japonicus | gall_tissue | - | 1972 |
| GCA_003691565.1_ASM369156v1 | Serratia_sp. | P2ACOL2 | Cerambycidae_sp. | larvae | Norway | 2017 |
| GCA_000214195.1_ASM21419v1 | Serratia_sp. | AS12 | rapeseed_plant | - | - | - |
| GCA_000214805.1_ASM21480v1 | Serratia_sp. | AS13 | rapeseed_plant | - | - | - |
| GCA_003812745.1_ASM381274v1 | Serratia_sp._(marcescens_sensu_lato) | FDAARGOS_506 | Homo_sapiens | Endotracheal_aspirate | - | 2015 |
| GCA_000695995.1_ASM69599v1 | Serratia_sp._(marcescens_sensu_lato) | FS14 | - | - | - | - |
| GCA_003719595.1_ASM371959v1 | Serratia_sp._(marcescens_sensu_lato) | LS_1 | Orthaga_achatina | - | - | 2016 |
| GCA_009905295.1_ASM990529v1 | Serratia_sp._(marcescens_sensu_lato) | NGAS9 | Solanum_tuberosum | rhizosphere_soil | Tanzania | 2018 |
| GCA_000747565.1_ASM74756v1 | Serratia_sp._(marcescens_sensu_lato) | SCBI | - | - | - | - |
| GCA_002935055.1_ASM293505v1 | Serratia_sp._(marcescens_sensu_lato) | SSNIH1 | - | - | USA | 2015 |
| GCA_001642805.2_ASM164280v2 | Serratia_surfactantfaciens | YD25 | - | rhizosphere_soil | China_Fujian | 2011 |
| GCA_014304635.1_ASM1430463v1 | Serratia_ureilytica | CC119 | plant | - | USA_TX_Corpus_Christi | 2017 |
| GCA_016728045.1_ASM1672804v1 | Serratia_ureilytica | FDAARGOS_1089 | - | - | Germany_Langen | - |
| AM1004_MZ | Serratia_sp. | AM1004_MZ | Dryococelus_australis_MZ | Melbourne_Zoo | Australia | 2015 |
| AM923_MZ | Serratia_sp. | AM923_MZ | Dryococelus_australis_MZ | Melbourne_Zoo | Australia | 2014 |
| This study | Serratia_sp. | CM2008_163 | Avian | Uvet_Melbourne | Australia | 2008 |
| This study | Serratia_sp. | CM2012_028 | Felis_catus | Uvet_Melbourne | Australia | 2012 |
| This study | Serratia_sp. | CM2012_118 | Felis_catus | Uvet_Melbourne | Australia | 2012 |
| This study | Serratia_sp. | CM2012_298 | Equus_caballus | Uvet_Melbourne | Australia | 2012 |
| This study | Serratia_sp. | CM2012_306 | Felis_catus | Uvet_Melbourne | Australia | 2012 |
| This study | Serratia_sp. | CM2014_932 | Equus_caballus | Uvet_Melbourne | Australia | 2014 |
| This study | Serratia_sp. | CM2015_078 | Felis_catus | Uvet_Melbourne | Australia | 2015 |
| This study | Serratia_sp. | CM2015_137 | Canis_domesticus | Uvet_Melbourne | Australia | 2015 |
| This study | Serratia_sp. | CM2015_244 | Felis_catus | Uvet_Melbourne | Australia | 2015 |
| This study | Serratia_sp. | CM2015_854 | Canis_domesticus | Uvet_Melbourne | Australia | 2015 |
| This study | Serratia_sp. | CM2016_091 | Canis_domesticus | Uvet_Melbourne | Australia | 2016 |
| This study | Serratia_sp. | CM2016_261 | Felis_catus | Uvet_Melbourne | Australia | 2016 |
| This study | Serratia_sp. | CM2016_324 | Felis_catus | Uvet_Melbourne | Australia | 2016 |
| This study | Serratia_sp. | CM2016_384 | Canis_domesticus | Uvet_Melbourne | Australia | 2016 |
| This study | Serratia_sp. | CM2017_569 | Canis_domesticus | Uvet_Melbourne | Australia | 2017 |
| This study | Serratia_sp. | CM2017_728 | Equus_caballus | Uvet_Melbourne | Australia | 2017 |
| This study | Serratia_sp. | CM2019_254 | Equus_caballus | Uvet_Melbourne | Australia | 2019 |
| This study | Serratia_sp. | CM2019_352 | Oryctolagus_cuniculus | Uvet_Melbourne | Australia | 2019 |
