# Supplementary material for: Healthcare-associated infections caused by chlorhexidine-tolerant Serratia marcescens carrying a promiscuous IncHI2 multi-drug resistance plasmid in a veterinary hospital
Source: PLoS One. 2022 Mar 17;17(3):e0264848. doi: 10.1371/journal.pone.0264848 (PMC8929579; doi:10.1371/journal.pone.0264848)
Supplement: S2 Table — (DOCX) [file pone.0264848.s005.docx]

TableS2. NCBI partial genomes of *S. marcescens* isolated from animals.

| Genbank_Accession | Organism | Strain | Host | Isolation_source | Country | Year |
| --- | --- | --- | --- | --- | --- | --- |
| GCA_004179515.1_ASM417951v1 | Serratia_marcescens | ICR003201 | Homo_sapiens | patient | France | 2016 |
| GCA_004179565.1_ASM417956v1 | Serratia_marcescens | ICR003202 | Homo_sapiens | patient | France | 2016 |
| GCA_008830745.1_ASM883074v1 | Serratia_marcescens | 188J2 | Homo_sapiens | rectal_swab | France | 2017 |
| GCA_008868645.1_ASM886864v1 | Serratia_marcescens | 2280 | Homo_sapiens | pulmonary_fluid | France | 2019 |
| GCA_013277695.1_ASM1327769v1 | Serratia_marcescens | 50986 | Canis_domesticus | - | France | 2009 |
| GCA_013277705.1_ASM1327770v1 | Serratia_marcescens | 51034 | Felis_catus | - | France | 2017 |
| GCA_013277715.1_ASM1327771v1 | Serratia_marcescens | 50995 | Felis_catus | - | France | 2014 |
| GCA_013277725.1_ASM1327772v1 | Serratia_marcescens | 51748 | - | vet clinic | France | 2019 |
| GCA_013280415.1_ASM1328041v1 | Serratia_marcescens | 51745 | Canis_domesticus | - | France | 2018 |
| GCA_011684105.1_ASM1168410v1 | Serratia_marcescens | 11H | bovine | feces | South_Africa | 2018 |
| GCA_013302905.1_ASM1330290v1 | Serratia_marcescens | ZZCCN01 | bovine | blood | China | 2018 |
|  |  |  |  |  |  |  |
