# Supplementary material for: Healthcare-associated infections caused by chlorhexidine-tolerant Serratia marcescens carrying a promiscuous IncHI2 multi-drug resistance plasmid in a veterinary hospital
Source: PLoS One. 2022 Mar 17;17(3):e0264848. doi: 10.1371/journal.pone.0264848 (PMC8929579; doi:10.1371/journal.pone.0264848)
Supplement: S3 Table — (DOCX) [file pone.0264848.s006.docx]

Table S3. NCBI partial and complete *S. marcescens* genomes used for the extended phylogenetic and plasmid analysis.

| accession1 | Strain | Host | Country | date |
| --- | --- | --- | --- | --- |
| GCA_000264275.1_ASM26427v1 | LCT_SM213 |  | _ | - |
| GCA_000292365.1_ASM29236v1 | W2_3 | Tilapia | Malaysia | - |
| GCA_000336425.1_ASM33642v1 | WW4 |  | _ | - |
| GCA_000342205.1_SerMar_1.0 | VGH107 | Homo_sapiens | Taiwan | Jul-2011 |
| GCA_000418815.1_S_marcescensMC620-1.0 | MC620 | Homo_sapiens | USA | - |
| GCA_000418835.1_S_marcescensMC6001-1.0 | MC6001 | Homo_sapiens | USA | - |
| GCA_000418855.2_S_marcescensMC6000-1.0 | MC6000 | Homo_sapiens | USA | - |
| GCA_000418875.1_S_marcescensMC460-1.0 | MC460 | Homo_sapiens | USA | - |
| GCA_000418895.1_S_marcescensMC459-1.0 | MC459 | Homo_sapiens | USA | - |
| GCA_000418915.1_S_marcescensMC458-1.0 | MC458 | Homo_sapiens | USA | - |
| GCA_000418935.1_S_marcescensAB42556419-isolate1-1.0 | AB42556419_isolate1 | Homo_sapiens | USA | - |
| GCA_000442375.1_S.marcescens_LCT-SM262 | LCT_SM262 |  | _ | - |
| GCA_000442455.1_S.marcescens_LCT-SM166 | LCT_SM166 |  | _ | - |
| GCA_000465615.2_GS_De_Novo_Assembly | EGD_HP20 |  | India | 09-Oct-2005 |
| GCA_000513215.1_DB11 | Db11 |  | _ | - |
| GCA_000521905.1_Serr_marc_BIDMC_50_V1 | BIDMC_50 |  | _ | - |
| GCA_000521925.1_Serr_marc_BIDMC_44_V1 | BIDMC_44 |  | _ | - |
| GCA_000633335.1_ASM63333v1 | H1q |  | Malaysia | - |
| GCA_000633555.1_ASM63355v1 | PH1a |  | Malaysia | - |
| GCA_000633695.1_Serr_marc_BIDMC_81_V1 | BIDMC_81 | Homo_sapiens | _ | 2013-09-18 |
| GCA_000633715.1_Serr_marc_BIDMC_80_V1 | BIDMC_80 | Homo_sapiens | _ | 2013-09-18 |
| GCA_000695485.1_smgenome | ATCC_14041 |  | USA | 2013 |
| GCA_000734475.1_ASM73447v1 | YDC563 | Homo_sapiens | USA | 30-Jan-2012 |
| GCA_000738535.1_ASM73853v1 | MCB | Oscheius_sp | South_Africa | 11-Apr-2013 |
| GCA_000739215.1_ASM73921v1 | NGS_ED_1015 | Homo_sapiens | United_Kingdom | 2013 |
| GCA_000743395.1_SAS_1 | CDC_813_60 |  | _ | - |
| GCA_000751195.1_Serratia_assembly | GCA_000751195 |  | _ | - |
| GCA_000783615.2_ASM78361v2 | FDAARGOS_79 | Homo_sapiens | USA | 03-Nov-2013 |
| GCA_000783915.2_ASM78391v2 | FDAARGOS_65 | Homo_sapiens | USA | 19-Oct-2013 |
| GCA_000783975.2_ASM78397v2 | FDAARGOS_62 | Homo_sapiens | USA | 15-Oct-2013 |
| GCA_000805875.1_SmRM66262_v1.0 | RM66262 | Homo_sapiens | Argentina | 2003 |
| GCA_000828775.1_ASM82877v1 | SM39 |  | _ | - |
| GCA_001007555.1_2015-04-28 | 90_166 |  | USA | 1990 |
| GCA_001022215.1_ASM102221v1 | CAV1492 | Homo_sapiens | USA | 2011-12 |
| GCA_001030265.1_Serr_marc_BWH57_V1 | BWH57 | Homo_sapiens | USA | 2014 |
| GCA_001034375.1_Serr_marc_BWH56_V1 | BWH56 | Homo_sapiens | USA | 2014 |
| GCA_001034395.1_Serr_marc_UCI87_V1 | UCI87 | Homo_sapiens | _ | 2014 |
| GCA_001034405.1_Serr_marc_UCI88_V1 | UCI88 | Homo_sapiens | _ | 2014 |
| GCA_001051865.1_ASM105186v1 | AH0650_Sm1 | Homo_sapiens | Australia | 20-Mar-2014 |
| GCA_001060335.1_ASM106033v1 | 1145_SMAR | Homo_sapiens | USA | - |
| GCA_001060585.1_ASM106058v1 | 1218_SMAR | Homo_sapiens | USA | - |
| GCA_001060625.1_ASM106062v1 | 1241_SMAR | Homo_sapiens | USA | - |
| GCA_001060655.1_ASM106065v1 | 1242_SMAR | Homo_sapiens | USA | - |
| GCA_001061145.1_ASM106114v1 | 1186_SMAR | Homo_sapiens | USA | - |
| GCA_001061195.1_ASM106119v1 | 1198_rep2_SMAR | Homo_sapiens | USA | - |
| GCA_001061225.1_ASM106122v1 | 1219_SMAR | Homo_sapiens | USA | - |
| GCA_001062235.1_ASM106223v1 | 1185_SMAR | Homo_sapiens | USA | - |
| GCA_001062285.1_ASM106228v1 | 1198_rep1_SMAR | Homo_sapiens | USA | - |
| GCA_001063125.1_ASM106312v1 | 276_SMAR | Homo_sapiens | USA | - |
| GCA_001063145.1_ASM106314v1 | 280_SMAR | Homo_sapiens | USA | - |
| GCA_001063175.1_ASM106317v1 | 286_SMAR | Homo_sapiens | USA | - |
| GCA_001063325.1_ASM106332v1 | 410_SMAR | Homo_sapiens | USA | - |
| GCA_001063375.1_ASM106337v1 | 420_SMAR | Homo_sapiens | USA | - |
| GCA_001064335.1_ASM106433v1 | 287_SMAR | Homo_sapiens | USA | - |
| GCA_001064345.1_ASM106434v1 | 290_SMAR | Homo_sapiens | USA | - |
| GCA_001064455.1_ASM106445v1 | 311_SMAR | Homo_sapiens | USA | - |
| GCA_001064715.1_ASM106471v1 | 370_SMAR | Homo_sapiens | USA | - |
| GCA_001064725.1_ASM106472v1 | 374_SMAR | Homo_sapiens | USA | - |
| GCA_001064835.1_ASM106483v1 | 395_SMAR | Homo_sapiens | USA | - |
| GCA_001064855.1_ASM106485v1 | 398_SMAR | Homo_sapiens | USA | - |
| GCA_001064975.1_ASM106497v1 | 454_SMAR | Homo_sapiens | USA | - |
| GCA_001065275.1_ASM106527v1 | 508_SMAR | Homo_sapiens | USA | - |
| GCA_001065325.1_ASM106532v1 | 532_SMAR | Homo_sapiens | USA | - |
| GCA_001065405.1_ASM106540v1 | 546_SSON | Homo_sapiens | USA | - |
| GCA_001065845.1_ASM106584v1 | 666_SMAR | Homo_sapiens | USA | - |
| GCA_001065935.1_ASM106593v1 | 684_SMAR | Homo_sapiens | USA | - |
| GCA_001066015.1_ASM106601v1 | 709_SMAR | Homo_sapiens | USA | - |
| GCA_001066945.1_ASM106694v1 | 698_SMAR | Homo_sapiens | USA | - |
| GCA_001067015.1_ASM106701v1 | 706_SMAR | Homo_sapiens | USA | - |
| GCA_001067375.1_ASM106737v1 | 790_SMAR | Homo_sapiens | USA | - |
| GCA_001068085.1_ASM106808v1 | 907_SMAR | Homo_sapiens | USA | - |
| GCA_001076625.1_ASM107662v1 | 294_SMAR | Homo_sapiens | USA | - |
| GCA_001280365.1_ASM128036v1 | RSC_14 | Solanum_nigrum | South_Korea | 2013 |
| GCA_001294565.1_ASM129456v1 | SmUNAM836 | Homo_sapiens | Mexico | Aug-2005 |
| GCA_001317205.2_56_S29v.02 | 945154301 | Homo_sapiens | South_Africa | 18-Apr-2013 |
| GCA_001317425.2_59_S30v.02 | 945174350 | Homo_sapiens | South_Africa | - |
| GCA_001417865.2_ASM141786v2 | B3R3 | Zea_mays | China | 2011 |
| GCA_001536325.1_12082_3_52 | 2880STDY5682955 | Homo_sapiens | United_Kingdom | 2008 |
| GCA_001536345.1_12082_2_67 | 2880STDY5682861 | Homo_sapiens | United_Kingdom | 2003 |
| GCA_001536365.1_12082_3_76 | 2880STDY5682980 | Homo_sapiens | United_Kingdom | 2009 |
| GCA_001536385.1_12082_3_28 | 2880STDY5682927 | Homo_sapiens | United_Kingdom | 2006 |
| GCA_001536405.1_12082_3_29 | 2880STDY5682928 | Homo_sapiens | United_Kingdom | 2006 |
| GCA_001536425.1_12082_3_92 | 2880STDY5682998 | Homo_sapiens | United_Kingdom | 2010 |
| GCA_001536445.1_12082_2_63 | 2880STDY5682856 | Homo_sapiens | United_Kingdom | 2003 |
| GCA_001536465.1_12082_2_70 | 2880STDY5682864 | Homo_sapiens | United_Kingdom | 2004 |
| GCA_001536485.1_12082_2_24 | 2880STDY5682815 | Homo_sapiens | United_Kingdom | 2001 |
| GCA_001536505.1_12082_2_64 | 2880STDY5682857 | Homo_sapiens | United_Kingdom | 2003 |
| GCA_001536525.1_12082_3_49 | 2880STDY5682952 | Homo_sapiens | United_Kingdom | 2008 |
| GCA_001536545.1_12082_3_88 | 2880STDY5682994 | Homo_sapiens | United_Kingdom | 2010 |
| GCA_001536565.1_12082_5_49 | 2880STDY5682999 | Homo_sapiens | United_Kingdom | 2010 |
| GCA_001536585.1_12082_5_39 | 2880STDY5682922 | Homo_sapiens | United_Kingdom | 2006 |
| GCA_001536605.1_12045_8_19 | 2880STDY5683026 | Homo_sapiens | United_Kingdom | 2008 |
| GCA_001536625.1_12082_3_56 | 2880STDY5682959 | Homo_sapiens | United_Kingdom | 2008 |
| GCA_001536645.1_12082_3_73 | 2880STDY5682977 | Homo_sapiens | United_Kingdom | 2009 |
| GCA_001536665.1_12082_3_57 | 2880STDY5682960 | Homo_sapiens | United_Kingdom | 2008 |
| GCA_001536685.1_12082_2_86 | 2880STDY5682882 | Homo_sapiens | United_Kingdom | 2004 |
| GCA_001536705.1_12082_3_61 | 2880STDY5682964 | Homo_sapiens | United_Kingdom | 2008 |
| GCA_001536725.1_12082_3_14 | 2880STDY5682906 | Homo_sapiens | United_Kingdom | 2005 |
| GCA_001536745.1_12082_3_86 | 2880STDY5682992 | Homo_sapiens | United_Kingdom | 2010 |
| GCA_001536765.1_12082_2_66 | 2880STDY5682859 | Homo_sapiens | United_Kingdom | 2003 |
| GCA_001536845.1_12082_3_30 | 2880STDY5682929 | Homo_sapiens | United_Kingdom | 2006 |
| GCA_001536895.1_12082_3_47 | 2880STDY5682950 | Homo_sapiens | United_Kingdom | 2007 |
| GCA_001536945.1_12082_3_63 | 2880STDY5682966 | Homo_sapiens | United_Kingdom | 2008 |
| GCA_001536965.1_12082_3_42 | 2880STDY5682942 | Homo_sapiens | United_Kingdom | 2007 |
| GCA_001536985.1_12082_2_79 | 2880STDY5682874 | Homo_sapiens | United_Kingdom | 2004 |
| GCA_001537005.1_12082_3_48 | 2880STDY5682951 | Homo_sapiens | United_Kingdom | 2007 |
| GCA_001537025.1_12082_2_54 | 2880STDY5682846 | Homo_sapiens | United_Kingdom | 2003 |
| GCA_001537045.1_12082_5_37 | 2880STDY5682914 | Homo_sapiens | United_Kingdom | 2006 |
| GCA_001537065.1_12082_3_13 | 2880STDY5682905 | Homo_sapiens | United_Kingdom | 2005 |
| GCA_001537085.1_12082_3_93 | 2880STDY5683001 | Homo_sapiens | United_Kingdom | 2010 |
| GCA_001537105.1_12045_8_17 | 2880STDY5683024 | Homo_sapiens | United_Kingdom | 2011 |
| GCA_001537125.1_12082_3_20 | 2880STDY5682916 | Homo_sapiens | United_Kingdom | 2006 |
| GCA_001537145.1_12082_2_28 | 2880STDY5682819 | Homo_sapiens | United_Kingdom | 2002 |
| GCA_001537165.1_12082_5_44 | 2880STDY5682944 | Homo_sapiens | United_Kingdom | 2007 |
| GCA_001537185.1_12082_3_26 | 2880STDY5682924 | Homo_sapiens | United_Kingdom | 2006 |
| GCA_001537205.1_12082_2_37 | 2880STDY5682828 | Homo_sapiens | United_Kingdom | 2002 |
| GCA_001537225.1_12082_2_72 | 2880STDY5682866 | Homo_sapiens | United_Kingdom | 2004 |
| GCA_001537245.1_12082_2_77 | 2880STDY5682871 | Homo_sapiens | United_Kingdom | 2004 |
| GCA_001537285.1_12045_8_16 | 2880STDY5683022 | Homo_sapiens | United_Kingdom | 2011 |
| GCA_001537305.1_12082_5_41 | 2880STDY5682930 | Homo_sapiens | United_Kingdom | 2007 |
| GCA_001537325.1_12082_2_53 | 2880STDY5682845 | Homo_sapiens | United_Kingdom | 2003 |
| GCA_001537345.1_12082_2_78 | 2880STDY5682872 | Homo_sapiens | United_Kingdom | 2004 |
| GCA_001537365.1_12082_3_5 | 2880STDY5682897 | Homo_sapiens | United_Kingdom | 2005 |
| GCA_001537405.1_12045_8_26 | 2880STDY5683035 | Homo_sapiens | United_Kingdom | 2006 |
| GCA_001537425.1_12082_2_48 | 2880STDY5682840 | Homo_sapiens | United_Kingdom | 2003 |
| GCA_001537445.1_12045_8_9 | 2880STDY5683014 | Homo_sapiens | United_Kingdom | 2010 |
| GCA_001537465.1_12082_2_82 | 2880STDY5682878 | Homo_sapiens | United_Kingdom | 2004 |
| GCA_001537485.1_12082_2_75 | 2880STDY5682869 | Homo_sapiens | United_Kingdom | 2004 |
| GCA_001537505.1_12082_5_35 | 2880STDY5682898 | Homo_sapiens | United_Kingdom | 2005 |
| GCA_001537525.1_12082_2_25 | 2880STDY5682816 | Homo_sapiens | United_Kingdom | 2001 |
| GCA_001537545.1_12082_3_18 | 2880STDY5682911 | Homo_sapiens | United_Kingdom | 2006 |
| GCA_001537565.1_12045_8_25 | 2880STDY5683034 | Homo_sapiens | United_Kingdom | 2006 |
| GCA_001537585.1_12082_3_71 | 2880STDY5682974 | Homo_sapiens | United_Kingdom | 2009 |
| GCA_001537605.1_12082_2_59 | 2880STDY5682851 | Homo_sapiens | United_Kingdom | 2003 |
| GCA_001537625.1_12082_3_27 | 2880STDY5682926 | Homo_sapiens | United_Kingdom | 2006 |
| GCA_001537645.1_12082_2_39 | 2880STDY5682830 | Homo_sapiens | United_Kingdom | 2002 |
| GCA_001537665.1_12082_2_85 | 2880STDY5682881 | Homo_sapiens | United_Kingdom | 2004 |
| GCA_001537685.1_12082_5_32 | 2880STDY5682853 | Homo_sapiens | United_Kingdom | 2003 |
| GCA_001537705.1_12082_3_82 | 2880STDY5682986 | Homo_sapiens | United_Kingdom | 2009 |
| GCA_001537725.1_12082_3_7 | 2880STDY5682900 | Homo_sapiens | United_Kingdom | 2005 |
| GCA_001537745.1_12082_3_95 | 2880STDY5683003 | Homo_sapiens | United_Kingdom | 2010 |
| GCA_001537765.1_12082_2_29 | 2880STDY5682820 | Homo_sapiens | United_Kingdom | 2002 |
| GCA_001537785.1_12045_8_1 | 2880STDY5683006 | Homo_sapiens | United_Kingdom | 2010 |
| GCA_001537805.1_12082_5_51 | 2880STDY5683016 | Homo_sapiens | United_Kingdom | 2010 |
| GCA_001537825.1_12045_8_21 | 2880STDY5683030 | Homo_sapiens | United_Kingdom | 2003 |
| GCA_001537845.1_12082_2_32 | 2880STDY5682823 | Homo_sapiens | United_Kingdom | 2002 |
| GCA_001537865.1_12082_5_46 | 2880STDY5682949 | Homo_sapiens | United_Kingdom | 2007 |
| GCA_001537885.1_12082_2_34 | 2880STDY5682825 | Homo_sapiens | United_Kingdom | 2002 |
| GCA_001537905.1_12082_5_34 | 2880STDY5682876 | Homo_sapiens | United_Kingdom | 2004 |
| GCA_001537925.1_12082_3_89 | 2880STDY5682995 | Homo_sapiens | United_Kingdom | 2010 |
| GCA_001538005.1_12082_3_46 | 2880STDY5682947 | Homo_sapiens | United_Kingdom | 2007 |
| GCA_001538115.1_12082_3_68 | 2880STDY5682971 | Homo_sapiens | United_Kingdom | 2009 |
| GCA_001538195.1_12045_8_14 | 2880STDY5683020 | Homo_sapiens | United_Kingdom | 2011 |
| GCA_001538275.1_12082_5_36 | 2880STDY5682912 | Homo_sapiens | United_Kingdom | 2006 |
| GCA_001538325.1_12082_5_42 | 2880STDY5682939 | Homo_sapiens | United_Kingdom | 2007 |
| GCA_001538345.1_12082_2_92 | 2880STDY5682888 | Homo_sapiens | United_Kingdom | 2005 |
| GCA_001538365.1_12082_2_91 | 2880STDY5682887 | Homo_sapiens | United_Kingdom | 2005 |
| GCA_001538385.1_12082_5_30 | 2880STDY5682835 | Homo_sapiens | United_Kingdom | 2002 |
| GCA_001538405.1_12082_3_65 | 2880STDY5682968 | Homo_sapiens | United_Kingdom | 2009 |
| GCA_001538425.1_12082_2_74 | 2880STDY5682868 | Homo_sapiens | United_Kingdom | 2004 |
| GCA_001538445.1_12082_2_56 | 2880STDY5682848 | Homo_sapiens | United_Kingdom | 2003 |
| GCA_001538465.1_12045_8_5 | 2880STDY5683010 | Homo_sapiens | United_Kingdom | 2010 |
| GCA_001538485.1_12045_8_15 | 2880STDY5683021 | Homo_sapiens | United_Kingdom | 2011 |
| GCA_001538505.1_12082_3_45 | 2880STDY5682945 | Homo_sapiens | United_Kingdom | 2007 |
| GCA_001538525.1_12082_2_33 | 2880STDY5682824 | Homo_sapiens | United_Kingdom | 2002 |
| GCA_001538545.1_12045_8_11 | 2880STDY5683017 | Homo_sapiens | United_Kingdom | 2010 |
| GCA_001538565.1_12082_3_96 | 2880STDY5683004 | Homo_sapiens | United_Kingdom | 2010 |
| GCA_001538585.1_12082_3_75 | 2880STDY5682979 | Homo_sapiens | United_Kingdom | 2009 |
| GCA_001538605.1_12082_2_30 | 2880STDY5682822 | Homo_sapiens | United_Kingdom | 2002 |
| GCA_001538625.1_12082_2_44 | 2880STDY5682836 | Homo_sapiens | United_Kingdom | 2002 |
| GCA_001538645.1_12082_3_15 | 2880STDY5682907 | Homo_sapiens | United_Kingdom | 2006 |
| GCA_001538665.1_12082_5_47 | 2880STDY5682975 | Homo_sapiens | United_Kingdom | 2009 |
| GCA_001538685.1_12082_3_64 | 2880STDY5682967 | Homo_sapiens | United_Kingdom | 2008 |
| GCA_001538705.1_12045_8_23 | 2880STDY5683032 | Homo_sapiens | United_Kingdom | 2006 |
| GCA_001538725.1_12082_2_60 | 2880STDY5682852 | Homo_sapiens | United_Kingdom | 2003 |
| GCA_001538745.1_12082_3_35 | 2880STDY5682934 | Homo_sapiens | United_Kingdom | 2007 |
| GCA_001538765.1_12082_2_35 | 2880STDY5682826 | Homo_sapiens | United_Kingdom | 2002 |
| GCA_001538785.1_12045_8_18 | 2880STDY5683025 | Homo_sapiens | United_Kingdom | 2011 |
| GCA_001538805.1_12082_2_55 | 2880STDY5682847 | Homo_sapiens | United_Kingdom | 2003 |
| GCA_001538825.1_12082_2_87 | 2880STDY5682883 | Homo_sapiens | United_Kingdom | 2004 |
| GCA_001538845.1_12082_3_90 | 2880STDY5682996 | Homo_sapiens | United_Kingdom | 2010 |
| GCA_001538865.1_12082_3_87 | 2880STDY5682993 | Homo_sapiens | United_Kingdom | 2010 |
| GCA_001538885.1_12082_3_32 | 2880STDY5682931 | Homo_sapiens | United_Kingdom | 2007 |
| GCA_001538905.1_12082_2_23 | 2880STDY5682814 | Homo_sapiens | United_Kingdom | 2001 |
| GCA_001538925.1_12082_3_9 | 2880STDY5682901 | Homo_sapiens | United_Kingdom | 2005 |
| GCA_001538945.1_12082_3_34 | 2880STDY5682933 | Homo_sapiens | United_Kingdom | 2007 |
| GCA_001538965.1_12082_3_39 | 2880STDY5682938 | Homo_sapiens | United_Kingdom | 2007 |
| GCA_001538985.1_12045_8_27 | 2880STDY5683036 | Homo_sapiens | United_Kingdom | 2006 |
| GCA_001539005.1_12082_5_33 | 2880STDY5682873 | Homo_sapiens | United_Kingdom | 2004 |
| GCA_001539025.1_12082_2_27 | 2880STDY5682818 | Homo_sapiens | United_Kingdom | 2002 |
| GCA_001539045.1_12082_3_36 | 2880STDY5682935 | Homo_sapiens | United_Kingdom | 2007 |
| GCA_001539065.1_12082_5_48 | 2880STDY5682988 | Homo_sapiens | United_Kingdom | 2010 |
| GCA_001539085.1_12082_3_12 | 2880STDY5682904 | Homo_sapiens | United_Kingdom | 2005 |
| GCA_001539105.1_12045_8_10 | 2880STDY5683015 | Homo_sapiens | United_Kingdom | 2010 |
| GCA_001539125.1_12045_8_6 | 2880STDY5683011 | Homo_sapiens | United_Kingdom | 2010 |
| GCA_001539145.1_12082_3_16 | 2880STDY5682908 | Homo_sapiens | United_Kingdom | 2006 |
| GCA_001539185.1_12045_8_4 | 2880STDY5683009 | Homo_sapiens | United_Kingdom | 2010 |
| GCA_001539205.1_12082_3_59 | 2880STDY5682962 | Homo_sapiens | United_Kingdom | 2008 |
| GCA_001539225.1_12045_8_7 | 2880STDY5683013 | Homo_sapiens | United_Kingdom | 2010 |
| GCA_001539245.1_12082_3_24 | 2880STDY5682921 | Homo_sapiens | United_Kingdom | 2006 |
| GCA_001539265.1_12082_2_61 | 2880STDY5682854 | Homo_sapiens | United_Kingdom | 2003 |
| GCA_001539285.1_12082_3_37 | 2880STDY5682936 | Homo_sapiens | United_Kingdom | 2007 |
| GCA_001539305.1_12045_8_2 | 2880STDY5683007 | Homo_sapiens | United_Kingdom | 2010 |
| GCA_001539325.1_12082_3_53 | 2880STDY5682956 | Homo_sapiens | United_Kingdom | 2008 |
| GCA_001539345.1_12082_2_88 | 2880STDY5682884 | Homo_sapiens | United_Kingdom | 2004 |
| GCA_001539365.1_12082_2_58 | 2880STDY5682850 | Homo_sapiens | United_Kingdom | 2003 |
| GCA_001539385.1_12082_3_19 | 2880STDY5682913 | Homo_sapiens | United_Kingdom | 2006 |
| GCA_001539405.1_12082_3_38 | 2880STDY5682937 | Homo_sapiens | United_Kingdom | 2007 |
| GCA_001539425.1_12082_2_81 | 2880STDY5682877 | Homo_sapiens | United_Kingdom | 2004 |
| GCA_001539445.1_12082_3_62 | 2880STDY5682965 | Homo_sapiens | United_Kingdom | 2008 |
| GCA_001539465.1_12082_5_50 | 2880STDY5683000 | Homo_sapiens | United_Kingdom | 2010 |
| GCA_001539485.1_12045_8_3 | 2880STDY5683008 | Homo_sapiens | United_Kingdom | 2010 |
| GCA_001539505.1_12082_2_40 | 2880STDY5682831 | Homo_sapiens | United_Kingdom | 2002 |
| GCA_001539525.1_12082_5_52 | 2880STDY5683027 | Homo_sapiens | United_Kingdom | 2009 |
| GCA_001539545.1_12082_3_50 | 2880STDY5682953 | Homo_sapiens | United_Kingdom | 2008 |
| GCA_001539565.1_12082_3_1 | 2880STDY5682893 | Homo_sapiens | United_Kingdom | 2005 |
| GCA_001539585.1_12082_2_69 | 2880STDY5682863 | Homo_sapiens | United_Kingdom | 2004 |
| GCA_001539605.1_12082_3_80 | 2880STDY5682984 | Homo_sapiens | United_Kingdom | 2009 |
| GCA_001539625.1_12082_3_44 | 2880STDY5682943 | Homo_sapiens | United_Kingdom | 2007 |
| GCA_001539645.1_12082_2_46 | 2880STDY5682838 | Homo_sapiens | United_Kingdom | 2003 |
| GCA_001539665.1_12082_3_51 | 2880STDY5682954 | Homo_sapiens | United_Kingdom | 2008 |
| GCA_001539685.1_12082_3_79 | 2880STDY5682983 | Homo_sapiens | United_Kingdom | 2009 |
| GCA_001539705.1_12082_2_51 | 2880STDY5682843 | Homo_sapiens | United_Kingdom | 2003 |
| GCA_001539725.1_12082_2_47 | 2880STDY5682839 | Homo_sapiens | United_Kingdom | 2003 |
| GCA_001539745.1_12082_2_93 | 2880STDY5682889 | Homo_sapiens | United_Kingdom | 2005 |
| GCA_001539765.1_12082_2_65 | 2880STDY5682858 | Homo_sapiens | United_Kingdom | 2003 |
| GCA_001539785.1_12082_2_36 | 2880STDY5682827 | Homo_sapiens | United_Kingdom | 2002 |
| GCA_001539805.1_12082_2_42 | 2880STDY5682834 | Homo_sapiens | United_Kingdom | 2002 |
| GCA_001539825.1_12082_3_74 | 2880STDY5682978 | Homo_sapiens | United_Kingdom | 2009 |
| GCA_001539845.1_12082_2_73 | 2880STDY5682867 | Homo_sapiens | United_Kingdom | 2004 |
| GCA_001539865.1_12082_3_11 | 2880STDY5682903 | Homo_sapiens | United_Kingdom | 2005 |
| GCA_001539885.1_12082_2_71 | 2880STDY5682865 | Homo_sapiens | United_Kingdom | 2004 |
| GCA_001539905.1_12082_3_58 | 2880STDY5682961 | Homo_sapiens | United_Kingdom | 2008 |
| GCA_001539925.1_12082_2_80 | 2880STDY5682875 | Homo_sapiens | United_Kingdom | 2004 |
| GCA_001539945.1_12082_3_60 | 2880STDY5682963 | Homo_sapiens | United_Kingdom | 2008 |
| GCA_001539965.1_12082_2_38 | 2880STDY5682829 | Homo_sapiens | United_Kingdom | 2002 |
| GCA_001539985.1_12045_8_24 | 2880STDY5683033 | Homo_sapiens | United_Kingdom | 2006 |
| GCA_001540005.1_12082_3_55 | 2880STDY5682958 | Homo_sapiens | United_Kingdom | 2008 |
| GCA_001540025.1_12082_3_40 | 2880STDY5682940 | Homo_sapiens | United_Kingdom | 2007 |
| GCA_001540045.1_12082_3_77 | 2880STDY5682981 | Homo_sapiens | United_Kingdom | 2009 |
| GCA_001540065.1_12082_3_84 | 2880STDY5682990 | Homo_sapiens | United_Kingdom | 2010 |
| GCA_001540085.1_12082_5_40 | 2880STDY5682925 | Homo_sapiens | United_Kingdom | 2006 |
| GCA_001540105.1_12082_3_10 | 2880STDY5682902 | Homo_sapiens | United_Kingdom | 2005 |
| GCA_001540125.1_12045_8_28 | 2880STDY5683037 | Homo_sapiens | United_Kingdom | 2007 |
| GCA_001540145.1_12082_3_33 | 2880STDY5682932 | Homo_sapiens | United_Kingdom | 2007 |
| GCA_001540165.1_12082_3_91 | 2880STDY5682997 | Homo_sapiens | United_Kingdom | 2010 |
| GCA_001540185.1_12082_2_41 | 2880STDY5682832 | Homo_sapiens | United_Kingdom | 2002 |
| GCA_001540205.1_12082_2_50 | 2880STDY5682842 | Homo_sapiens | United_Kingdom | 2003 |
| GCA_001540225.1_12045_8_22 | 2880STDY5683031 | Homo_sapiens | United_Kingdom | 2006 |
| GCA_001540245.1_12082_3_22 | 2880STDY5682918 | Homo_sapiens | United_Kingdom | 2006 |
| GCA_001540265.1_12082_2_57 | 2880STDY5682849 | Homo_sapiens | United_Kingdom | 2003 |
| GCA_001540285.1_12082_3_21 | 2880STDY5682917 | Homo_sapiens | United_Kingdom | 2006 |
| GCA_001540305.1_12082_2_94 | 2880STDY5682890 | Homo_sapiens | United_Kingdom | 2005 |
| GCA_001540325.1_12082_2_95 | 2880STDY5682891 | Homo_sapiens | United_Kingdom | 2005 |
| GCA_001540345.1_12082_3_67 | 2880STDY5682970 | Homo_sapiens | United_Kingdom | 2009 |
| GCA_001540365.1_12082_2_68 | 2880STDY5682862 | Homo_sapiens | United_Kingdom | 2003 |
| GCA_001540385.1_12082_2_84 | 2880STDY5682880 | Homo_sapiens | United_Kingdom | 2004 |
| GCA_001540405.1_12082_5_45 | 2880STDY5682948 | Homo_sapiens | United_Kingdom | 2007 |
| GCA_001540425.1_12082_2_62 | 2880STDY5682855 | Homo_sapiens | United_Kingdom | 2003 |
| GCA_001540445.1_12082_3_72 | 2880STDY5682976 | Homo_sapiens | United_Kingdom | 2009 |
| GCA_001540465.1_12082_5_38 | 2880STDY5682915 | Homo_sapiens | United_Kingdom | 2006 |
| GCA_001540485.1_12082_3_23 | 2880STDY5682919 | Homo_sapiens | United_Kingdom | 2006 |
| GCA_001540505.1_12082_3_78 | 2880STDY5682982 | Homo_sapiens | United_Kingdom | 2009 |
| GCA_001540525.1_12082_2_76 | 2880STDY5682870 | Homo_sapiens | United_Kingdom | 2004 |
| GCA_001540545.1_12082_3_66 | 2880STDY5682969 | Homo_sapiens | United_Kingdom | 2009 |
| GCA_001540565.1_12082_3_94 | 2880STDY5683002 | Homo_sapiens | United_Kingdom | 2010 |
| GCA_001540585.1_12082_3_85 | 2880STDY5682991 | Homo_sapiens | United_Kingdom | 2010 |
| GCA_001540605.1_12082_2_83 | 2880STDY5682879 | Homo_sapiens | United_Kingdom | 2004 |
| GCA_001540625.1_12082_2_89 | 2880STDY5682885 | Homo_sapiens | United_Kingdom | 2004 |
| GCA_001540645.1_12045_8_13 | 2880STDY5683019 | Homo_sapiens | United_Kingdom | 2010 |
| GCA_001540665.1_12045_8_12 | 2880STDY5683018 | Homo_sapiens | United_Kingdom | 2010 |
| GCA_001540685.1_12082_3_6 | 2880STDY5682899 | Homo_sapiens | United_Kingdom | 2005 |
| GCA_001540705.1_12082_3_70 | 2880STDY5682973 | Homo_sapiens | United_Kingdom | 2009 |
| GCA_001540725.1_12082_2_52 | 2880STDY5682844 | Homo_sapiens | United_Kingdom | 2003 |
| GCA_001540745.1_12082_3_4 | 2880STDY5682896 | Homo_sapiens | United_Kingdom | 2005 |
| GCA_001540765.1_12082_2_96 | 2880STDY5682892 | Homo_sapiens | United_Kingdom | 2005 |
| GCA_001540785.1_12082_3_83 | 2880STDY5682987 | Homo_sapiens | United_Kingdom | 2009 |
| GCA_001540805.1_12082_2_45 | 2880STDY5682837 | Homo_sapiens | United_Kingdom | 2003 |
| GCA_001540825.1_12082_3_54 | 2880STDY5682957 | Homo_sapiens | United_Kingdom | 2008 |
| GCA_001564475.1_12082_3_81 | 2880STDY5682985 | Homo_sapiens | United_Kingdom | 2009 |
| GCA_001566695.1_ASM156669v1 | 3691F | Homo_sapiens | Brazil | 16-Oct-2014 |
| GCA_001594385.1_ASM159438v1 | ICU1_2a | Homo_sapiens | USA | 30-Oct-2014 |
| GCA_001643155.1_ASM164315v1 | 092713_C_TSB | Opossum | USA | 27-Sep-2013 |
| GCA_001672055.1_ASM167205v1 | U36365 | Homo_sapiens | India | 30-Dec-2015 |
| GCA_001716895.1_ASM171689v1 | UENF_22GI |  | Brazil | 2013 |
| GCA_001756295.1_ASM175629v1 | TM | termite | USA | Apr-2013 |
| GCA_001853455.1_ASM185345v1 | ano1 | Anopheles_stephensi | _ | - |
| GCA_001853495.1_ASM185349v1 | ano2 | Anopheles_stephensi | _ | - |
| GCA_001889685.1_ASM188968v1 | sicaria_Ss1_ | Apis_mellifera | USA | 20-Dec-2014 |
| GCA_001902635.1_ASM190263v1 | MSU97 |  | Venezuela | 2016 |
| GCA_001908015.1_ASM190801v1 | 189 | Homo_sapiens | Russia | 2012-10-11 |
| GCA_001908035.1_ASM190803v1 | 99 | Homo_sapiens | Russia | 2009-09-11 |
| GCA_001909165.1_ASM190916v1 | SM03 | Homo_sapiens | India | 16-Oct-2012 |
| GCA_001914155.1_ASM191415v1 | GCA_001914155 | Homo_sapiens | USA | 2013/2014 |
| GCA_001932655.1_ASM193265v1 | AS1 | Anopheles_stephensi | China | 2013-02-28 |
| GCA_001940505.1_ASM194050v1 | BJL200 |  | _ | - |
| GCA_001975745.1_ASM197574v1 | 19F | Atelopus_zeteki | USA | Mar-2016 |
| GCA_002007925.2_ASM200792v2 | 1274 | Agave_sisalana | Brazil | 2010 |
| GCA_002029205.1_ASM202920v1 | CAPREX_SY13 |  | United_Kingdom | 2016-02-01 |
| GCA_002029225.1_ASM202922v1 | CAPREX_SY21 |  | United_Kingdom | 2016-02-01 |
| GCA_002094145.1_ASM209414v1 | MEW06 |  | China | 15-Oct-2014 |
| GCA_002104095.1_ASM210409v1 | D_3 | Homo_sapiens | USA | 2014 |
| GCA_002104105.1_ASM210410v1 | D_1 | Homo_sapiens | USA | 2014 |
| GCA_002104115.1_ASM210411v1 | D_2 | Homo_sapiens | USA | 2014 |
| GCA_002108655.1_ASM210865v1 | Z6 |  | China | Jul-2016 |
| GCA_002118055.1_ASM211805v1 | ML2637 | Homo_sapiens | South_Africa | 08-Jul-2016 |
| GCA_002152845.1_ASM215284v1 | MGH135 | Homo_sapiens | USA | 2015 |
| GCA_002153355.1_ASM215335v1 | MGH136 | Homo_sapiens | USA | 2015 |
| GCA_002205475.1_ASM220547v1 | S2I7 |  | India | 10-Jan-2015 |
| GCA_002220515.1_ASM222051v1 | UMH2 | Homo_sapiens | USA | Jan-2014 |
| GCA_002220535.1_ASM222053v1 | UMH8 | Homo_sapiens | USA | Aug-2013 |
| GCA_002220555.1_ASM222055v1 | UMH9 | Homo_sapiens | USA | Aug-2014 |
| GCA_002220575.1_ASM222057v1 | UMH11 | Homo_sapiens | USA | May-2014 |
| GCA_002220595.1_ASM222059v1 | UMH12 | Homo_sapiens | USA | Aug-2014 |
| GCA_002220615.1_ASM222061v1 | UMH1 | Homo_sapiens | USA | Nov-2013 |
| GCA_002220635.1_ASM222063v1 | UMH5 | Homo_sapiens | USA | Apr-2014 |
| GCA_002220655.1_ASM222065v1 | UMH3 | Homo_sapiens | USA | Mar-2014 |
| GCA_002220675.1_ASM222067v1 | UMH6 | Homo_sapiens | USA | Jul-2013 |
| GCA_002220695.1_ASM222069v1 | UMH10 | Homo_sapiens | USA | May-2014 |
| GCA_002220715.1_ASM222071v1 | UMH7 | Homo_sapiens | USA | Sep-2013 |
| GCA_002250685.1_ASM225068v1 | at10508 | Homo_sapiens | Austria | 2017-01-21 |
| GCA_002264105.1_ASM226410v1 | SE768 | Homo_sapiens | China | 07-May-2014 |
| GCA_002264175.1_ASM226417v1 | SE4145 | Homo_sapiens | China | 21-Jul-2015 |
| GCA_002264285.1_ASM226428v1 | SE3605 | Homo_sapiens | China | 08-May-2015 |
| GCA_002265665.1_ASM226566v1 | EGD_HP20_1 |  | India | 2013-10-24 |
| GCA_002325865.1_ASM232586v1 | GCA_002325865 |  | USA | - |
| GCA_002592035.1_ASM259203v1 | KHCo_24B | Gossypium_hirsutum | India | 10-Oct-2015 |
| GCA_002738105.1_ASM273810v1 | 14ES | Homo_sapiens | Romania | 03-May-2012 |
| GCA_002738145.1_ASM273814v1 | 4TM | Homo_sapiens | Romania | 05-Feb-2015 |
| GCA_002738155.1_ASM273815v1 | 7209 | Homo_sapiens | Romania | 28-Mar-2013 |
| GCA_002738185.1_ASM273818v1 | 9580 | Homo_sapiens | Romania | 08-Aug-2015 |
| GCA_002762595.1_ASM276259v1 | K27 |  | _ | - |
| GCA_002810285.1_ASM281028v1 | 12TM | Homo_sapiens | Romania | 21-Oct-2014 |
| GCA_002872515.1_ASM287251v1 | SOLR4 |  | Brazil | 2015 |
| GCA_002886905.1_ASM288690v1 | YDC107_2 | Homo_sapiens | USA | - |
| GCA_002887105.1_ASM288710v1 | YD509_2 | Homo_sapiens | USA | - |
| GCA_002899335.1_ASM289933v1 | JES_110 |  | USA | Aug-2016 |
| GCA_002899355.1_ASM289935v1 | ADJS_2D_White | Neoscapteriscus_borellii | USA | 02-Jun-2015 |
| GCA_002899375.1_ASM289937v1 | ADJS_2C_Purple | Neoscapteriscus_borellii | USA | 03-Jun-2015 |
| GCA_002899415.1_ASM289941v1 | ADJS_2C_Red | Neoscapteriscus_borellii | USA | 01-Jun-2015 |
| GCA_002915435.1_ASM291543v1 | KZ19 | Apis_mellifera | USA | Apr-2016 |
| GCA_002915445.1_ASM291544v1 | KZ11 | Apis_mellifera | USA | Apr-2016 |
| GCA_002915475.1_ASM291547v1 | KZ2 | Apis_mellifera | USA | Apr-2016 |
| GCA_002920335.1_ASM292033v1 | ID147729 | Homo_sapiens | Canada | - |
| GCA_002920345.1_ASM292034v1 | ID148138 | Homo_sapiens | Canada | - |
| GCA_002920375.1_ASM292037v1 | ID148696 |  | Canada | - |
| GCA_002920385.1_ASM292038v1 | ID148299 |  | Canada | - |
| GCA_002920415.1_ASM292041v1 | ID148137 | Homo_sapiens | Canada | - |
| GCA_002920435.1_ASM292043v1 | ID149855 | Homo_sapiens | Canada | - |
| GCA_002920455.1_ASM292045v1 | ID147991 | Homo_sapiens | Canada | - |
| GCA_002920475.1_ASM292047v1 | ID149856 | Homo_sapiens | Canada | - |
| GCA_002920485.1_ASM292048v1 | ID147728 | Homo_sapiens | Canada | - |
| GCA_002920515.1_ASM292051v1 | ID148587 | Homo_sapiens | Canada | - |
| GCA_002946295.1_ASM294629v1 | GCA_002946295 | Homo_sapiens | China | 2015-07-28 |
| GCA_002947235.1_ASM294723v1 | AR_0027 |  | _ | - |
| GCA_002996885.1_ASM299688v1 | AR_0091 |  | _ | - |
| GCA_002997125.1_ASM299712v1 | AR_0099 |  | _ | - |
| GCA_003031545.1_ASM303154v1 | 95 | Homo_sapiens | USA | 2015 |
| GCA_003031645.1_ASM303164v1 | BWH_35 | Homo_sapiens | USA | 2012 |
| GCA_003071565.1_ASM307156v1 | AR_0124 |  | _ | - |
| GCA_003071585.1_ASM307158v1 | AR_0130 |  | _ | - |
| GCA_003071605.1_ASM307160v1 | AR_0123 |  | _ | - |
| GCA_003071625.1_ASM307162v1 | AR_0121 |  | _ | - |
| GCA_003146705.1_ASM314670v1 | CAV1761 | Homo_sapiens | USA | 01-Mar-2014 |
| GCA_003182655.1_ASM318265v1 | SGAir0764 |  | Singapore | 07-Dec-2016 |
| GCA_003186475.1_ASM318647v1 | 332 | Homo_sapiens | USA | 2016 |
| GCA_003204075.1_ASM320407v1 | AR_0131 |  | _ | - |
| GCA_003204405.1_ASM320440v1 | AR_0122 |  | _ | - |
| GCA_003204525.1_ASM320452v1 | 1756 | Homo_sapiens | Brazil | 2013 |
| GCA_003204555.1_ASM320455v1 | 630 | Homo_sapiens | Brazil | 2010 |
| GCA_003204565.1_ASM320456v1 | 642 | Homo_sapiens | Brazil | 2010 |
| GCA_003204595.1_ASM320459v1 | 1052 | Homo_sapiens | Brazil | 2011 |
| GCA_003204615.1_ASM320461v1 | 1058 | Homo_sapiens | Brazil | 2011 |
| GCA_003204635.1_ASM320463v1 | 1309 | Homo_sapiens | Brazil | 2012 |
| GCA_003204645.1_ASM320464v1 | 1763 | Homo_sapiens | Brazil | 2013 |
| GCA_003204675.1_ASM320467v1 | 1053 | Homo_sapiens | Brazil | 2011 |
| GCA_003204685.1_ASM320468v1 | 1057 | Homo_sapiens | Brazil | 2011 |
| GCA_003204715.1_ASM320471v1 | 1257 | Homo_sapiens | Brazil | 2012 |
| GCA_003204725.1_ASM320472v1 | 1509 | Homo_sapiens | Brazil | 2012 |
| GCA_003204755.1_ASM320475v1 | 1283 | Homo_sapiens | Brazil | 2012 |
| GCA_003204765.1_ASM320476v1 | 1299 | Homo_sapiens | Brazil | 2012 |
| GCA_003204795.1_ASM320479v1 | 1707 | Homo_sapiens | Brazil | 2013 |
| GCA_003204805.1_ASM320480v1 | 4116 | Homo_sapiens | Brazil | 2014 |
| GCA_003204835.1_ASM320483v1 | 1056 | Homo_sapiens | Brazil | 2011 |
| GCA_003204855.1_ASM320485v1 | 1703 | Homo_sapiens | Brazil | 2013 |
| GCA_003204865.1_ASM320486v1 | 2031 |  | Brazil | 2012 |
| GCA_003204885.1_ASM320488v1 | 1844 | Homo_sapiens | Brazil | 2013 |
| GCA_003204905.1_ASM320490v1 | 1673 | Homo_sapiens | Brazil | 2013 |
| GCA_003204935.1_ASM320493v1 | 2032 |  | Brazil | 2012 |
| GCA_003204985.1_ASM320498v1 | 2039 |  | Brazil | 2012 |
| GCA_003204995.1_ASM320499v1 | 1054 | Homo_sapiens | Brazil | 2011 |
| GCA_003241435.1_ASM324143v1 | GCA_003241435 |  | USA | 01-Oct-2013 |
| GCA_003284885.1_ASM328488v1 | 1510 | Homo_sapiens | Brazil | 2012 |
| GCA_003355135.1_ASM335513v1 | N4_5 |  | USA | 01-Jan-1995 |
| GCA_003400385.1_ASM340038v1 | CRE94 | Homo_sapiens | USA | 05-Dec-2016 |
| GCA_003400545.1_ASM340054v1 | CRE78 | Homo_sapiens | USA | 30-Sep-2016 |
| GCA_003400615.1_ASM340061v1 | CRE72 | Homo_sapiens | USA | 26-Aug-2016 |
| GCA_003401215.1_ASM340121v1 | CRE44 | Homo_sapiens | USA | 25-Dec-2015 |
| GCA_003402775.1_ASM340277v1 | CRE35 | Homo_sapiens | USA | 02-Sep-2015 |
| GCA_003425745.1_ASM342574v1 | 163 | Homo_sapiens | Argentina | 2016 |
| GCA_003428265.1_ASM342826v1 | KCTC_42172 |  | _ | - |
| GCA_003484665.1_ASM348466v1 | GCA_003484665 |  | _ | - |
| GCA_003516165.1_ASM351616v1 | KS10 |  | USA | 07-Aug-2006 |
| GCA_003516185.1_ASM351618v1 | EL1 |  | USA | 29-Jun-2002 |
| GCA_003591985.1_ASM359198v1 | RCE05_sm | Homo_sapiens | Russia | 2013 |
| GCA_003602305.1_ASM360230v1 | 4F_69 |  | Spain | 04-Nov-1970 |
| GCA_003605765.1_ASM360576v1 | O7_16 | Homo_sapiens | Spain | 21-Nov-2016 |
| GCA_003605795.1_ASM360579v1 | O11_16 | Homo_sapiens | Spain | 09-Dec-2016 |
| GCA_003626775.1_ASM362677v1 | 6F_69 |  | Spain | 06-Nov-1970 |
| GCA_003666885.1_ASM366688v1 | Mex_1 | Homo_sapiens | Mexico | 2007 |
| GCA_003666905.1_ASM366690v1 | CHE4 | Homo_sapiens | Canada | 2004 |
| GCA_003666915.1_ASM366691v1 | AW | Homo_sapiens | Switzerland | 2006 |
| GCA_003666945.1_ASM366694v1 | S8 | Homo_sapiens | United_Kingdom | 1982 |
| GCA_003666955.1_ASM366695v1 | USA_1 | Homo_sapiens | USA | 2012 |
| GCA_003666965.1_ASM366696v1 | S6 | Homo_sapiens | United_Kingdom | 1982 |
| GCA_003703735.1_ASM370373v1 | 15F_69 |  | Spain | 15-Nov-1970 |
| GCA_003935125.1_ASM393512v1 | SM_196 |  | Pakistan | 2016-04 |
| GCA_003935135.1_ASM393513v1 | SM_321 |  | Pakistan | 2016-04 |
| GCA_003935145.1_ASM393514v1 | SM_362 |  | Pakistan | 2016-07 |
| GCA_003935225.1_ASM393522v1 | SM_153 |  | Pakistan | 2016-01 |
| GCA_003935235.1_ASM393523v1 | SM_079 |  | Pakistan | 2016-01 |
| GCA_003935245.1_ASM393524v1 | SM_076 |  | Pakistan | 2016-01 |
| GCA_003935265.1_ASM393526v1 | SM_188 |  | Pakistan | 2016-03 |
| GCA_003935355.1_ASM393535v1 | SM_045 |  | Pakistan | 2016-02 |
| GCA_003935635.1_ASM393563v1 | SM_037 |  | Pakistan | 2016-02 |
| GCA_003935755.1_ASM393575v1 | SM_204 |  | Pakistan | 2016-01 |
| GCA_003935775.1_ASM393577v1 | SM_190 |  | Pakistan | 2016-03 |
| GCA_003935805.1_ASM393580v1 | SM_164 |  | Pakistan | 2016-02 |
| GCA_003935875.1_ASM393587v1 | SM_090 |  | Pakistan | 2016-02 |
| GCA_003957865.1_ASM395786v1 | WWI31 |  | USA | 07-Aug-2006 |
| GCA_003957915.1_ASM395791v1 | PDL100 |  | USA | 1999 |
| GCA_003957935.1_ASM395793v1 | KS65 |  | USA | 07-Aug-2006 |
| GCA_003957945.1_ASM395794v1 | KS40 |  | USA | 07-Aug-2006 |
| GCA_003957975.1_ASM395797v1 | KS25 |  | USA | 07-Aug-2006 |
| GCA_003957985.1_ASM395798v1 | KS23 |  | USA | 07-Aug-2006 |
| GCA_003958045.1_ASM395804v1 | KS12 |  | USA | 07-Aug-2006 |
| GCA_003958065.1_ASM395806v1 | KS9 |  | USA | 07-Aug-2006 |
| GCA_003958075.1_ASM395807v1 | EL121 |  | USA | 29-Jul-2003 |
| GCA_003958085.1_ASM395808v1 | KS1 |  | USA | 07-Aug-2006 |
| GCA_003958095.1_ASM395809v1 | EL120 |  | USA | 29-Jul-2003 |
| GCA_003958135.1_ASM395813v1 | EL118 |  | USA | 29-Jul-2003 |
| GCA_003958165.1_ASM395816v1 | EL117 |  | USA | 29-Jul-2003 |
| GCA_003958175.1_ASM395817v1 | EL115 |  | USA | 29-Jul-2003 |
| GCA_003958185.1_ASM395818v1 | EL114 |  | USA | 29-Jul-2003 |
| GCA_003958205.1_ASM395820v1 | EL113 |  | USA | 29-Jul-2003 |
| GCA_003958215.1_ASM395821v1 | EL108 |  | USA | 28-Jul-2003 |
| GCA_003958265.1_ASM395826v1 | EL98 |  | USA | 28-Jul-2003 |
| GCA_003958275.1_ASM395827v1 | EL97 |  | USA | 28-Jul-2003 |
| GCA_003958295.1_ASM395829v1 | EL96 |  | USA | 28-Jul-2003 |
| GCA_003958305.1_ASM395830v1 | EL95 |  | USA | 28-Jul-2003 |
| GCA_003958335.1_ASM395833v1 | EL85 |  | USA | 28-Jul-2003 |
| GCA_003958365.1_ASM395836v1 | EL84 |  | USA | 28-Jul-2003 |
| GCA_003958385.1_ASM395838v1 | EL3 |  | USA | 29-Jun-2002 |
| GCA_003958395.1_ASM395839v1 | KS45 |  | USA | 07-Aug-2006 |
| GCA_003958405.1_ASM395840v1 | KS16 |  | USA | 07-Aug-2006 |
| GCA_003958415.1_ASM395841v1 | KS5 |  | USA | 07-Aug-2006 |
| GCA_003958445.1_ASM395844v1 | EL122 |  | USA | 29-Jul-2003 |
| GCA_003958485.1_ASM395848v1 | EL110 |  | USA | 28-Jul-2003 |
| GCA_003958495.1_ASM395849v1 | EL109 |  | USA | 28-Jul-2003 |
| GCA_003958505.1_ASM395850v1 | EL41 |  | USA | 28-Sep-2003 |
| GCA_003958535.1_ASM395853v1 | EL6 |  | USA | 29-Jun-2002 |
| GCA_003958565.1_ASM395856v1 | EL60 |  | USA | 28-Jul-2003 |
| GCA_003958575.1_ASM395857v1 | EL116 |  | USA | 29-Jul-2003 |
| GCA_003958605.1_ASM395860v1 | EL119 |  | USA | 29-Jul-2003 |
| GCA_003967055.1_ASM396705v1 | AS_1 |  | Japan | - |
| GCA_004109355.1_ASM410935v1 | O1_16 | Homo_sapiens | Spain | 11-Nov-2016 |
| GCA_004109385.1_ASM410938v1 | O10_16 | Homo_sapiens | Spain | 08-Dec-2016 |
| GCA_004179515.1_ASM417951v1 | ICR003201 | Homo_sapiens | France | 2016 |
| GCA_004179565.1_ASM417956v1 | ICR003202 | Homo_sapiens | France | 2016 |
| GCA_004196355.1_ASM419635v1 | RPH1 | Rhodnius_prolixus | Brazil | 2016 |
| GCA_004196365.1_ASM419636v1 | RPA1 | Rhodnius_prolixus | Brazil | 2016 |
| GCA_004326135.1_ASM432613v1 | SM6a |  | _ | 01-Jan-1966 |
| GCA_004378065.1_ASM437806v1 | 13F_69 |  | Spain | 13-Nov-1970 |
| GCA_004570635.1_ASM457063v1 | N2 | Homo_sapiens | Egypt | Feb-2017 |
| GCA_004684145.1_ASM468414v1 | DSM_17174 |  | Japan | 1998-01-01 |
| GCA_006494455.1_ASM649445v1 | M4 | Homo_sapiens | Chile | 24-Apr-2014 |
| GCA_006711125.1_ASM671112v1 | WVU_004 | Homo_sapiens | USA | Apr-2019 |
| GCA_006711145.1_ASM671114v1 | WVU_005 | Homo_sapiens | USA | Apr-2019 |
| GCA_006711245.1_ASM671124v1 | WVU_006 | Homo_sapiens | USA | Apr-2019 |
| GCA_006711405.1_ASM671140v1 | WVU_007 | Homo_sapiens | USA | Apr-2019 |
| GCA_006711525.1_ASM671152v1 | WVU_008 | Homo_sapiens | USA | Apr-2019 |
| GCA_006715005.1_ASM671500v1 | 106R |  | USA | - |
| GCA_006716725.1_ASM671672v1 | WVU_009 | Homo_sapiens | USA | Apr-2019 |
| GCA_006716825.1_ASM671682v1 | WVU_010 | Homo_sapiens | USA | Apr-2019 |
| GCA_006842785.1_ASM684278v1 | WVU_002 | Homo_sapiens | USA | 2018-07-02 |
| GCA_006974205.1_ASM697420v1 | ATCC_13880 |  | USA | 1969 |
| GCA_007280475.1_ASM728047v1 | S11 |  | Germany | 2015-05-02 |
| GCA_007954045.1_ASM795404v1 | KCJ3K309 | Homo_sapiens | USA | Jun-2019 |
| GCA_007954245.1_ASM795424v1 | KCJ3K435 | Homo_sapiens | USA | Jul-2019 |
| GCA_008011745.1_ASM801174v1 | S16 |  | Germany | 2015-05-02 |
| GCA_008011755.1_ASM801175v1 | S14 |  | Germany | 2015-05-02 |
| GCA_008011775.1_ASM801177v1 | S12 |  | Germany | 2015-05-02 |
| GCA_008011855.1_ASM801185v1 | S3 |  | Germany | 2015-05-02 |
| GCA_008011865.1_ASM801186v1 | S5 |  | Germany | 2015-05-02 |
| GCA_008122445.1_ASM812244v1 | C7 | Sarracenia | USA | 06-Nov-2010 |
| GCA_008180355.1_ASM818035v1 | KCJ3K308 | Homo_sapiens | USA | 2019-06-20 |
| GCA_008364225.1_ASM836422v1 | S13 |  | Germany | 2015-05-02 |
| GCA_008364235.1_ASM836423v1 | S18 |  | Germany | 2015-05-02 |
| GCA_008364245.1_ASM836424v1 | S15 |  | Germany | 2015-05-02 |
| GCA_008364255.1_ASM836425v1 | S10 |  | Germany | 2015-05-02 |
| GCA_008364265.2_ASM836426v2 | S7_1 |  | Germany | 2015-05-02 |
| GCA_008364335.1_ASM836433v1 | S2 |  | Germany | 2015-05-02 |
| GCA_008830745.1_ASM883074v1 | 188J2 | Homo_sapiens | France | 2017 |
| GCA_008868645.1_ASM886864v1 | 2280 | Homo_sapiens | France | 2019 |
| GCA_008931425.1_ASM893142v1 | E28 |  | Australia | 12-Oct-2012 |
| GCA_009746745.1_ASM974674v1 | BTL07 |  | Bangladesh | - |
| GCA_009833165.1_ASM983316v1 | 2838a | Homo_sapiens | China | 17-Dec-2018 |
| GCA_009833175.1_ASM983317v1 | 3024a | Homo_sapiens | China | 25-Dec-2018 |
| GCA_009833185.1_ASM983318v1 | 3460 | Homo_sapiens | China | 10-Jan-2019 |
| GCA_009833195.1_ASM983319v1 | 4201a | Homo_sapiens | China | 27-Feb-2019 |
| GCA_009833205.1_ASM983320v1 | 2730 | Homo_sapiens | China | 05-Dec-2018 |
| GCA_009833265.1_ASM983326v1 | 1203 | Homo_sapiens | China | 23-Sep-2018 |
| GCA_009833285.1_ASM983328v1 | 1614 | Homo_sapiens | China | 12-Oct-2018 |
| GCA_009833295.1_ASM983329v1 | C91 | Homo_sapiens | China | 04-Jun-2018 |
| GCA_009833305.1_ASM983330v1 | 1140 | Homo_sapiens | China | 24-Aug-2018 |
| GCA_009833345.1_ASM983334v1 | 1330 | Homo_sapiens | China | 30-Sep-2018 |
| GCA_009833365.1_ASM983336v1 | C40 | Homo_sapiens | China | 28-Apr-2018 |
| GCA_009833385.1_ASM983338v1 | C3 | Homo_sapiens | China | 17-Apr-2018 |
| GCA_009833405.1_ASM983340v1 | C21 | Homo_sapiens | China | 20-Apr-2018 |
| GCA_009833425.1_ASM983342v1 | 3717 | Homo_sapiens | China | 07-Feb-2019 |
| GCA_009833465.1_ASM983346v1 | 3725 | Homo_sapiens | China | 31-Jan-2019 |
| GCA_009833525.1_ASM983352v1 | 2686 | Homo_sapiens | China | 04-Dec-2018 |
| GCA_009833535.1_ASM983353v1 | 2291 | Homo_sapiens | China | 12-Nov-2018 |
| GCA_009833545.1_ASM983354v1 | 2079 | Homo_sapiens | China | 31-Oct-2018 |
| GCA_009833585.1_ASM983358v1 | 1758 | Homo_sapiens | China | 17-Oct-2018 |
| GCA_009833595.1_ASM983359v1 | 1486 | Homo_sapiens | China | 27-Sep-2018 |
| GCA_009833625.1_ASM983362v1 | 972 | Homo_sapiens | China | 14-Sep-2018 |
| GCA_009833635.1_ASM983363v1 | C156 | Homo_sapiens | China | 09-Jul-2018 |
| GCA_009833655.1_ASM983365v1 | 939 | Homo_sapiens | China | 22-Aug-2018 |
| GCA_009833675.1_ASM983367v1 | C141 | Homo_sapiens | China | 04-Jul-2018 |
| GCA_009833705.1_ASM983370v1 | C153 | Homo_sapiens | China | 10-Jul-2018 |
| GCA_009833725.1_ASM983372v1 | C126 | Homo_sapiens | China | 26-Jun-2018 |
| GCA_009833745.1_ASM983374v1 | C110a | Homo_sapiens | China | 16-Jun-2018 |
| GCA_009833755.1_ASM983375v1 | C112 | Homo_sapiens | China | 15-Jun-2018 |
| GCA_009833765.1_ASM983376v1 | C83 | Homo_sapiens | China | 28-May-2018 |
| GCA_009833805.1_ASM983380v1 | C64 | Homo_sapiens | China | 17-May-2018 |
| GCA_009833815.1_ASM983381v1 | C60 | Homo_sapiens | China | 17-May-2018 |
| GCA_009833845.1_ASM983384v1 | C45 | Homo_sapiens | China | 26-Apr-2018 |
| GCA_009833865.1_ASM983386v1 | C47 | Homo_sapiens | China | 30-Apr-2018 |
| GCA_009833875.1_ASM983387v1 | C59 | Homo_sapiens | China | 18-May-2018 |
| GCA_009833905.1_ASM983390v1 | C8 | Homo_sapiens | China | 12-Apr-2018 |
| GCA_009833915.1_ASM983391v1 | C5 | Homo_sapiens | China | 13-Apr-2018 |
| GCA_009834305.1_ASM983430v1 | N10A28 | Apis_mellifera | USA | 23-Feb-2011 |
| GCA_009858195.1_ASM985819v1 | 1602 | Homo_sapiens | China | 2018-10-21 |
| GCA_009909345.1_ASM990934v1 | 4201 | Homo_sapiens | China | 27-Feb-2019 |
| GCA_009909365.1_ASM990936v1 | 3024 | Homo_sapiens | China | 25-Dec-2018 |
| GCA_009909385.1_ASM990938v1 | 1140_ | Homo_sapiens | China | 05-Sep-2018 |
| GCA_009909405.1_ASM990940v1 | 2838 | Homo_sapiens | China | 17-Dec-2018 |
| GCA_009909425.1_ASM990942v1 | C110b | Homo_sapiens | China | 16-Jun-2018 |
| GCA_009936295.1_ASM993629v1 | ATCC_274 |  | _ | - |
| GCA_009938955.1_ASM993895v1 | SER_525 | Homo_sapiens | USA | 2018 |
| GCA_009939145.1_ASM993914v1 | SER_502 | Homo_sapiens | USA | 2018 |
| GCA_009939185.1_ASM993918v1 | SER_501 | Homo_sapiens | USA | 2018 |
| GCA_010078255.1_ASM1007825v1 | SER_514 | Homo_sapiens | USA | 2018 |
| GCA_010078335.1_ASM1007833v1 | SER_505 | Homo_sapiens | USA | 2018 |
| GCA_010078355.1_ASM1007835v1 | SER_508 | Homo_sapiens | USA | 2018 |
| GCA_010092645.1_ASM1009264v1 | SER_523 | Homo_sapiens | USA | 2018 |
| GCA_010092665.1_ASM1009266v1 | SER_520 | Homo_sapiens | USA | 2018 |
| GCA_010092675.1_ASM1009267v1 | SER_522 | Homo_sapiens | USA | 2018 |
| GCA_010092745.1_ASM1009274v1 | SER_518 | Homo_sapiens | USA | 2018 |
| GCA_010092755.1_ASM1009275v1 | SER_500 | Homo_sapiens | USA | 2018 |
| GCA_010092765.1_ASM1009276v1 | SER_517 | Homo_sapiens | USA | 2018 |
| GCA_010499315.1_ASM1049931v1 | S722 | Homo_sapiens | China | 11-Dec-2017 |
| GCA_010499325.1_ASM1049932v1 | S1464 | Homo_sapiens | China | 02-Apr-2019 |
| GCA_010499335.1_ASM1049933v1 | S1461 | Homo_sapiens | China | 03-Apr-2019 |
| GCA_010499345.1_ASM1049934v1 | S717 | Homo_sapiens | China | 20-Nov-2017 |
| GCA_010499355.1_ASM1049935v1 | S716 | Homo_sapiens | China | 12-Nov-2017 |
| GCA_010499415.1_ASM1049941v1 | S697 | Homo_sapiens | China | 08-Nov-2017 |
| GCA_010499445.1_ASM1049944v1 | S708 | Homo_sapiens | China | 19-Oct-2017 |
| GCA_010588255.1_ASM1058825v1 | AS012490 | Homo_sapiens | USA | 2016-04-23 |
| GCA_010590205.1_ASM1059020v1 | AS012470 | Homo_sapiens | USA | 2016-01-20 |
| GCA_010590285.1_ASM1059028v1 | AS012466 | Homo_sapiens | USA | 2015-07-23 |
| GCA_010590485.1_ASM1059048v1 | AS012454 | Homo_sapiens | USA | 2015-09-08 |
| GCA_010590545.1_ASM1059054v1 | AS012452 | Homo_sapiens | USA | 2015-08-05 |
| GCA_010590895.1_ASM1059089v1 | AS012433 | Homo_sapiens | USA | 2015-02-03 |
| GCA_010590935.1_ASM1059093v1 | AS012431 | Homo_sapiens | USA | 2015-01-31 |
| GCA_010590995.1_ASM1059099v1 | AS012428 | Homo_sapiens | USA | 2015-01-26 |
| GCA_010591095.1_ASM1059109v1 | AS012422 | Homo_sapiens | USA | 2016-03-30 |
| GCA_010592785.1_ASM1059278v1 | AS012388 | Homo_sapiens | USA | 2015-10-14 |
| GCA_010594485.1_ASM1059448v1 | AS012375 | Homo_sapiens | USA | 2016-06-08 |
| GCA_010597575.1_ASM1059757v1 | AS012333 | Homo_sapiens | USA | 2014-05-22 |
| GCA_010597745.1_ASM1059774v1 | AS012323 | Homo_sapiens | USA | 2016-05-02 |
| GCA_010597835.1_ASM1059783v1 | AS012322 | Homo_sapiens | USA | 2016-04-14 |
| GCA_010598015.1_ASM1059801v1 | AS012309 | Homo_sapiens | USA | 2015-07-26 |
| GCA_010598185.1_ASM1059818v1 | AS012298 | Homo_sapiens | USA | 2015-10-06 |
| GCA_010598195.1_ASM1059819v1 | AS012297 | Homo_sapiens | USA | 2015-10-06 |
| GCA_010598525.1_ASM1059852v1 | AS012269 | Homo_sapiens | USA | 2016-04-21 |
| GCA_010598685.1_ASM1059868v1 | AS012260 | Homo_sapiens | USA | 2015-12-01 |
| GCA_010598745.1_ASM1059874v1 | AS012259 | Homo_sapiens | USA | 2015-11-24 |
| GCA_010598915.1_ASM1059891v1 | AS012250 | Homo_sapiens | USA | 2015-09-09 |
| GCA_010599015.1_ASM1059901v1 | AS012240 | Homo_sapiens | USA | 2015-06-29 |
| GCA_010602435.1_ASM1060243v1 | AS012296 | Homo_sapiens | USA | 2015-09-21 |
| GCA_010612285.1_ASM1061228v1 | AS012458 | Homo_sapiens | USA | 2015-05-05 |
| GCA_010612305.1_ASM1061230v1 | AS012386 | Homo_sapiens | USA | 2015-10-02 |
| GCA_010612345.1_ASM1061234v1 | AS012324 | Homo_sapiens | USA | 2015-07-13 |
| GCA_010612385.1_ASM1061238v1 | AS012299 | Homo_sapiens | USA | 2015-11-04 |
| GCA_011029435.1_ASM1102943v1 | SMM_61 | Homo_sapiens | Lebanon | 2018-11-10 |
| GCA_011029825.1_ASM1102982v1 | SMM_28 | Homo_sapiens | Lebanon | 2017-01-16 |
| GCA_011602465.1_ASM1160246v1 | BP2 | Jatropha_curcas | Brazil | 2010 |
| GCA_011684105.1_ASM1168410v1 | 11H | Bos_taurus | South_Africa | 2018-04-18 |
| GCA_011769885.1_ASM1176988v1 | SER00094 | Homo_sapiens | USA | 2017 |
| GCA_012273115.1_ASM1227311v1 | FDAARGOS_659 | Homo_sapiens | USA | - |
| GCA_012952305.1_ASM1295230v1 | SER00066 | Homo_sapiens | USA | 2017 |
| GCA_012956545.1_ASM1295654v1 | SR415 | Homo_sapiens | USA | Nov-2019 |
| GCA_012956635.1_ASM1295663v1 | S1SWMC |  | Brazil | 2018 |
| GCA_013047045.1_ASM1304704v1 | C2016001558 | Homo_sapiens | USA | 01-Jan-2016 |
| GCA_013112395.1_ASM1311239v1 | FZSF02 |  | China | Oct-2014 |
| GCA_013122155.1_ASM1312215v1 | FY | Drosophila_melanogaster | China | 2016-07-25 |
| GCA_013169055.1_ASM1316905v1 | C1_05 | Homo_sapiens | Colombia | 2013-09-14 |
| GCA_013169905.1_ASM1316990v1 | C1_15 | Homo_sapiens | Colombia | 2013-11-22 |
| GCA_013169945.1_ASM1316994v1 | C1_207 | Homo_sapiens | Colombia | 2015-08-17 |
| GCA_013169995.1_ASM1316999v1 | C3_37 | Homo_sapiens | Colombia | 2015-02-06 |
| GCA_013170015.1_ASM1317001v1 | C1_69 | Homo_sapiens | Colombia | 2014-05-20 |
| GCA_013170095.1_ASM1317009v1 | C3_26_2 | Homo_sapiens | Colombia | 2014-11-28 |
| GCA_013256815.1_ASM1325681v1 | LY1 | Cell_culture | China | 2019-08-15 |
| GCA_013277695.1_ASM1327769v1 | 50986 | Canis_familiaris | France | 2009-09-10 |
| GCA_013277705.1_ASM1327770v1 | 51034 | Felis_catus | France | 2017-01-16 |
| GCA_013277715.1_ASM1327771v1 | 50995 | Felis_catus | France | 2014-08-25 |
| GCA_013277725.1_ASM1327772v1 | 51748 |  | France | 2019-01-07 |
| GCA_013280415.1_ASM1328041v1 | 51745 | Canis_familiaris | France | 2018-09-14 |
| GCA_013302905.1_ASM1330290v1 | ZZCCN01 | Bos_taurus | China | 2018-09-11 |
| GCA_013309735.1_ASM1330973v1 | P60_CPSm | Homo_sapiens | Spain | 13-Dec-2016 |
| GCA_013309755.1_ASM1330975v1 | H28_CPSm | Homo_sapiens | Spain | 27-Nov-2017 |
| GCA_013309765.1_ASM1330976v1 | M52_CPSm | Homo_sapiens | Spain | 01-Oct-2018 |
| GCA_013309775.1_ASM1330977v1 | I54_CPSm | Homo_sapiens | Spain | 22-Jan-2018 |
| GCA_013309805.1_ASM1330980v1 | B77_CPSm | Homo_sapiens | Spain | 09-Mar-2017 |
| GCA_013367735.1_ASM1336773v1 | JW_CZ2 |  | China | 2014-06-04 |
| GCA_013369025.1_ASM1336902v1 | 1912768W |  | China | 2017-12-01 |
| GCA_013369395.1_ASM1336939v1 | 1912768WR |  | China | 2017-12-01 |
| GCA_013377375.1_ASM1337737v1 | 1912768R |  | China | 2015-09-01 |
| GCA_013425845.1_ASM1342584v1 | ICU_4 | Homo_sapiens | Brazil | 2013 |
| GCA_013425865.1_ASM1342586v1 | ICU_3 | Homo_sapiens | Brazil | 2013 |
| GCA_013425885.1_ASM1342588v1 | ICU_2 | Homo_sapiens | Brazil | 2013 |
| GCA_013426135.1_ASM1342613v1 | 12_2010 | Homo_sapiens | Canada | 2010 |
| GCA_013426155.1_ASM1342615v1 | 11_2010 | Homo_sapiens | Canada | 2010 |
| GCA_014333335.1_ASM1433333v1 | MGH246 | Homo_sapiens | USA | - |
| GCA_014595515.1_ASM1459551v1 | Sm3 | Homo_sapiens | Japan | 2009 |
| GCA_014595555.1_ASM1459555v1 | Sm4 | Homo_sapiens | Japan | 2009 |
| GCA_014595565.1_ASM1459556v1 | Sm1 | Homo_sapiens | Japan | 2009 |
| GCA_014595605.1_ASM1459560v1 | Sm7 | Homo_sapiens | Japan | 2009 |
| GCA_014595635.1_ASM1459563v1 | Sm6 | Homo_sapiens | Japan | 2009 |
| GCA_014596125.1_ASM1459612v1 | Sm2 | Homo_sapiens | Japan | 2009 |
| GCA_014596145.1_ASM1459614v1 | Sm10 | Homo_sapiens | Japan | 2010 |
| GCA_014596155.1_ASM1459615v1 | Sm8 | Homo_sapiens | Japan | 2009 |
| GCA_014596165.1_ASM1459616v1 | Sm14 | Homo_sapiens | Japan | 2010 |
| GCA_014596205.1_ASM1459620v1 | Sm11 | Homo_sapiens | Japan | 2010 |
| GCA_014596225.1_ASM1459622v1 | Sm18 | Homo_sapiens | Japan | 2010 |
| GCA_014596245.1_ASM1459624v1 | Sm20 | Homo_sapiens | Japan | 2010 |
| GCA_014947025.1_ASM1494702v1 | CFSAN059607 | Homo_sapiens | Pakistan | 2004 |
| GCA_014947035.1_ASM1494703v1 | CFSAN059609 | Homo_sapiens | Pakistan | 1998 |
| GCA_014947055.1_ASM1494705v1 | CFSAN059606 | Homo_sapiens | Pakistan | 2004 |
| GCA_015074945.1_ASM1507494v1 | SCH909 | Homo_sapiens | Greece | 1988 |
| GCA_015160915.1_ASM1516091v1 | SCQ1 | Bombyx_mori | China | May-2009 |
| GCA_015209035.1_ASM1520903v1 | H8980R | Homo_sapiens | South_Africa | 19-Feb-2019 |
| GCA_015548865.1_ASM1554886v1 | 1001295B_180824_A7 | Homo_sapiens | USA | 2018-08-24 |
| GCA_900029885.1_Sm_SMB2099 | SMB2099 |  | _ | - |
| GCA_900108835.1_SM1978 | GCA_900108835 |  | _ | - |
| GCA_900456855.1_59318_A01 | NCTC13920 |  | _ | - |
| GCA_900456915.1_56433_D01 | NCTC13382 |  | _ | - |
| GCA_900457055.1_32117_E02 | NCTC10211 |  | _ | - |
| GCA_900518895.1_NIG-Sm-8-18 | GCA_900518895 |  | _ | - |
| GCA_900518925.1_NIG-Sm-2-17 | GCA_900518925 |  | _ | - |
| GCA_900518945.1_NIG-Sm-3-18 | GCA_900518945 |  | _ | - |
| GCA_900518955.1_NIG-Sm-6-18 | GCA_900518955 |  | _ | - |
| GCA_900518975.1_NIG-Sm-1-17 | GCA_900518975 |  | _ | - |
| GCA_902165925.1_26009_2_8 | 4928STDY7387874 |  | _ | - |
| GCA_902166755.1_26009_2_35 | 4928STDY7387938 |  | _ | - |
| GCA_902386095.1_UHGG_MGYG-HGUT-02350 | GCA_902386095 | Homo_sapiens | _ | - |
| GCA_902387935.1_UHGG_MGYG-HGUT-02518 | GCA_902387935 | Homo_sapiens | _ | - |
| GCA_902754045.1_SFB6 | GCA_902754045 |  | _ | - |
| GCA_904866365.1_MSB1_9C | GCA_904866365 |  | _ | - |
