# Supplementary material for: Healthcare-associated infections caused by chlorhexidine-tolerant Serratia marcescens carrying a promiscuous IncHI2 multi-drug resistance plasmid in a veterinary hospital
Source: PLoS One. 2022 Mar 17;17(3):e0264848. doi: 10.1371/journal.pone.0264848 (PMC8929579; doi:10.1371/journal.pone.0264848)
Supplement: S4 Table — (DOCX) [file pone.0264848.s007.docx]

Table S4. Genes present in the chlorhexidine-tolerant strains CM2015_854 and CM2017_569 but absent from the chlorhexidine-susceptible strains.

| Locus_tag | | Roary | Genomic Island Island_Viewer_4 | | Prokka_annotation | | KofamKOALA | | | | | | | | | | BlastP_BacMet_database | | | | | | | | | | |
| --- | --- | --- | --- | --- | --- | --- | --- | --- | --- | --- | --- | --- | --- | --- | --- | --- | --- | --- | --- | --- | --- | --- | --- | --- | --- | --- | --- |
| CM2015_854 | CM2017_569 | Annotation | CM2015_854 | CM2017_569 | CM2015_854 | CM2017_569 | KO CM2015-854 | thrshld CM2015-854 | score CM2015-854 | E-value CM2015-854 | KO definition CM2015-854 | KO CM2017_569 | thrshld CM2017_569 | score CM2017_569 | E-value CM2017_569 | KO definition CM2017_569 | Subject_ID | pident | length | mismatch | gapopen | qstart | qend | sstart | send | evalue | bitscore |
| LBHGKGNI_00307 | LHADMLFC_04765 | Tellurite resistance protein TehA | . | . | NA | NA | K03304 | 173.13 | 309.5 | 1.70E-94 | tellurite resistance protein | K03304 | 173.13 | 307.2 | 9.40E-94 | tellurite resistance protein | BAC0384\|tehA\|sp\|P25396\|TEHA_ECOLI | 48.387 | 310 | 160 | 0 | 7 | 316 | 6 | 315 | 2.81E-86 | 258 |
| LBHGKGNI_00404 | LHADMLFC_00804 | Multiple antibiotic resistance protein MarA | Yes | . | marA Multiple antibiotic resistance protein MarA 430570:430977 reverse MW:15970 | NA | . | . | . | . | . | . | . | . | . | . | NA | NA | NA | NA | NA | NA | NA | NA | NA | NA | NA |
| LBHGKGNI_00405 | LHADMLFC_00805 | Transcriptional regulatory protein BaeR | Yes | . | baeR_1 Transcriptional regulatory protein BaeR 431323:431982 forward MW:24590 | NA | K18144 | 262.03 | 275 | 3.40E-84 | two-component system, OmpR family, response regulator AdeR | K18144 | 262.03 | 273.7 | 9.60E-84 | two-component system, OmpR family, response regulator AdeR | NA | NA | NA | NA | NA | NA | NA | NA | NA | NA | NA |
| LBHGKGNI_00406 | LHADMLFC_00807 | Adaptive-response sensory-kinase SasA | Yes | . | sasA_1 Adaptive-response sensory-kinase SasA 432024:433163 forward MW:42742 | NA | K18143 | 344.37 | 368.7 | 1.90E-112 | two-component system, OmpR family, sensor histidine kinase AdeS [EC:2.7.13.3] | . | . | . | . | . | NA | NA | NA | NA | NA | NA | NA | NA | NA | NA | NA |
| LBHGKGNI_00407 | LHADMLFC_00808 | putative efflux pump periplasmic linker TtgA | Yes | Yes | ttgA putative efflux pump periplasmic linker TtgA 433589:434371 forward MW:27430 | mdtE Multidrug resistance protein MdtE 878192:879313 forward MW:40013 | . | . | . | . | . | . | . | . | . | . | NA | NA | NA | NA | NA | NA | NA | NA | NA | NA | NA |
| LBHGKGNI_00408 | LHADMLFC_00809 | Multidrug export protein AcrF | Yes | Yes | czcA_1 Cobalt-zinc-cadmium resistance protein CzcA 434371:437424 forward MW:110810 | acrF Multidrug export protein AcrF 879313:882366 forward MW:110912 | K21134 | 1304.73 | 1314 | 0 | multidrug efflux pump | K21134 | 1304.73 | 1314.6 | 0 | multidrug efflux pump | BAC0242\|mexK\|tr\|Q9HXW4\|Q9HXW4_PSEAE | 45.142 | 1019 | 546 | 4 | 9 | 1015 | 3 | 1020 | 0 | 842 |
| LBHGKGNI_00410 | LHADMLFC_00811 | Intermembrane transport lipoprotein PqiC | . | . | NA | NA | K09857 | 89.93 | 186.5 | 3.80E-57 | uncharacterized protein | K09857 | 89.93 | 188.8 | 7.80E-58 | uncharacterized protein | NA | NA | NA | NA | NA | NA | NA | NA | NA | NA | NA |
| LBHGKGNI_00411 | LHADMLFC_00812 | Intermembrane transport protein PqiB | . | . | NA | NA | K06192 | 246.23 | 598.4 | 1.10E-181 | paraquat-inducible protein B | K06192 | 246.23 | 598.8 | 9.50E-182 | paraquat-inducible protein B | NA | NA | NA | NA | NA | NA | NA | NA | NA | NA | NA |
| LBHGKGNI_00412 | LHADMLFC_00813 | Intermembrane transport protein PqiA | Yes | . | pqiA_1 Intermembrane transport protein PqiA 440625:441848 reverse MW:45539 | NA | K03808 | 53.43 | 415.2 | 2.20E-126 | paraquat-inducible protein A | K03808 | 53.43 | 415.9 | 1.50E-126 | paraquat-inducible protein A | NA | NA | NA | NA | NA | NA | NA | NA | NA | NA | NA |
| LBHGKGNI_00413 | LHADMLFC_00814 | putative protein | Yes | . | LBHGKGNI_00413 hypothetical protein 442008:442937 reverse MW:35435 | NA | K09824 | 88.47 | 295.8 | 2.90E-90 | uncharacterized protein | K09824 | 88.47 | 295.8 | 3.20E-90 | uncharacterized protein | NA | NA | NA | NA | NA | NA | NA | NA | NA | NA | NA |
| LBHGKGNI_00414 | LHADMLFC_00815 | Ferri-bacillibactin esterase BesA | Yes | . | besA_1 Ferri-bacillibactin esterase BesA 443532:444311 forward MW:29288 | NA | . | . | . | . | . | . | . | . | . | . | NA | NA | NA | NA | NA | NA | NA | NA | NA | NA | NA |
| LBHGKGNI_00417 | LHADMLFC_00818 | HTH-type transcriptional regulator DmlR | . | . | NA | NA | . | . | . | . | . | . | . | . | . | . | NA | NA | NA | NA | NA | NA | NA | NA | NA | NA | NA |
| LBHGKGNI_00507 | LHADMLFC_00900 | tRNA nuclease CdiA | . | . | NA | NA | K15125 | 281.23 | 335.9 | 3.50E-102 | filamentous hemagglutinin | K15125 | 281.23 | 335.8 | 4.30E-102 | filamentous hemagglutinin | NA | NA | NA | NA | NA | NA | NA | NA | NA | NA | NA |
| LBHGKGNI_00508 | LHADMLFC_00901 | Hemolysin transporter protein ShlB | . | . | NA | NA | . | . | . | . | . | . | . | . | . | . | NA | NA | NA | NA | NA | NA | NA | NA | NA | NA | NA |
| LBHGKGNI_00596 | LHADMLFC_00969 | Protein UshA | . | . | NA | NA | . | . | . | . | . | K11751 | 431.43 | 484.7 | 4.00E-147 | 5'-nucleotidase / UDP-sugar diphosphatase [EC:3.1.3.5 3.6.1.45] | NA | NA | NA | NA | NA | NA | NA | NA | NA | NA | NA |
| LBHGKGNI_00604 | LHADMLFC_00978 | HTH-type transcriptional activator AllS | . | . | NA | NA | K10972 | 363.8 | 482.3 | 3.50E-147 | LysR family transcriptional regulator, transcriptional activator of the allD operon | K10972 | 363.8 | 482.3 | 3.80E-147 | LysR family transcriptional regulator, transcriptional activator of the allD operon | NA | NA | NA | NA | NA | NA | NA | NA | NA | NA | NA |
| LBHGKGNI_00605 | LHADMLFC_00979 | Ureidoglycolate lyase | . | . | NA | NA | K01483 | 51.3 | 188.1 | 1.20E-57 | ureidoglycolate lyase [EC:4.3.2.3] | K01483 | 51.3 | 188.1 | 1.40E-57 | ureidoglycolate lyase [EC:4.3.2.3] | NA | NA | NA | NA | NA | NA | NA | NA | NA | NA | NA |
| LBHGKGNI_00606 | LHADMLFC_00980 | HTH-type transcriptional repressor AllR | . | . | NA | NA | K10973 | 323.37 | 369.8 | 3.90E-113 | IclR family transcriptional regulator, negative regulator of allantoin and glyoxylate utilization operons | K10973 | 323.37 | 369.6 | 4.60E-113 | IclR family transcriptional regulator, negative regulator of allantoin and glyoxylate utilization operons | BAC0196\|iclR\|sp\|P16528\|ICLR_ECOLI | 43.446 | 267 | 148 | 2 | 5 | 268 | 8 | 274 | 1.69E-78 | 234 |
| LBHGKGNI_00607 | LHADMLFC_00981 | Glyoxylate carboligase | . | . | NA | NA | K01608 | 692.17 | 1108 | 0 | tartronate-semialdehyde synthase [EC:4.1.1.47] | K01608 | 692.17 | 1108 | 0 | tartronate-semialdehyde synthase [EC:4.1.1.47] | NA | NA | NA | NA | NA | NA | NA | NA | NA | NA | NA |
| LBHGKGNI_00608 | LHADMLFC_00982 | Hydroxypyruvate isomerase | . | . | NA | NA | K01816 | 143.07 | 357.7 | 5.00E-109 | hydroxypyruvate isomerase [EC:5.3.1.22] | K01816 | 143.07 | 357.7 | 5.40E-109 | hydroxypyruvate isomerase [EC:5.3.1.22] | NA | NA | NA | NA | NA | NA | NA | NA | NA | NA | NA |
| LBHGKGNI_00609 | LHADMLFC_00983 | 2-hydroxy-3-oxopropionate reductase | . | . | NA | NA | K00042 | 390.6 | 426.8 | 5.50E-130 | 2-hydroxy-3-oxopropionate reductase [EC:1.1.1.60] | K00042 | 390.6 | 427.3 | 4.20E-130 | 2-hydroxy-3-oxopropionate reductase [EC:1.1.1.60] | NA | NA | NA | NA | NA | NA | NA | NA | NA | NA | NA |
| LBHGKGNI_00610 | LHADMLFC_00984 | Putative metabolite transport protein NicT | . | . | NA | NA | . | . | . | . | . | . | . | . | . | . | NA | NA | NA | NA | NA | NA | NA | NA | NA | NA | NA |
| LBHGKGNI_00611 | LHADMLFC_00985 | Glycerate 3-kinase | . | . | NA | NA | K00865 | 63.23 | 531.8 | 1.10E-161 | glycerate 2-kinase [EC:2.7.1.165] | K00865 | 63.23 | 533.6 | 3.60E-162 | glycerate 2-kinase [EC:2.7.1.165] | NA | NA | NA | NA | NA | NA | NA | NA | NA | NA | NA |
| LBHGKGNI_00612 | LHADMLFC_00986 | (S)-ureidoglycine aminohydrolase | . | Yes | NA | allE (S)-ureidoglycine aminohydrolase 1066336:1067121 reverse MW:28842 | K14977 | 67.07 | 292.4 | 3.00E-89 | (S)-ureidoglycine aminohydrolase [EC:3.5.3.26] | K14977 | 67.07 | 292.4 | 3.30E-89 | (S)-ureidoglycine aminohydrolase [EC:3.5.3.26] | NA | NA | NA | NA | NA | NA | NA | NA | NA | NA | NA |
| LBHGKGNI_00613 | LHADMLFC_00987 | putative sulfoacetate transporter SauU | . | Yes | NA | sauU_1 putative sulfoacetate transporter SauU 1067179:1068438 reverse MW:44988 | . | . | . | . | . | . | . | . | . | . | NA | NA | NA | NA | NA | NA | NA | NA | NA | NA | NA |
| LBHGKGNI_00614 | LHADMLFC_00988 | Allantoate amidohydrolase | Yes | Yes | allC Allantoate amidohydrolase 647448:648695 reverse MW:45763 | allC Allantoate amidohydrolase 1068435:1069682 reverse MW:45791 | K02083 | 457.87 | 463.8 | 4.40E-141 | allantoate deiminase [EC:3.5.3.9] | K02083 | 457.87 | 462.5 | 1.10E-140 | allantoate deiminase [EC:3.5.3.9] | NA | NA | NA | NA | NA | NA | NA | NA | NA | NA | NA |
| LBHGKGNI_00615 | LHADMLFC_00989 | Ureidoglycolate dehydrogenase (NAD(+)) | Yes | Yes | allD Ureidoglycolate dehydrogenase (NAD(+)) 648868:649917 reverse MW:37618 | allD Ureidoglycolate dehydrogenase (NAD(+)) 1069855:1070904 reverse MW:37618 | K00073 | 370.8 | 528.6 | 5.40E-161 | ureidoglycolate dehydrogenase (NAD+) [EC:1.1.1.350] | K00073 | 370.8 | 528.6 | 5.80E-161 | ureidoglycolate dehydrogenase (NAD+) [EC:1.1.1.350] | NA | NA | NA | NA | NA | NA | NA | NA | NA | NA | NA |
| LBHGKGNI_00616 | LHADMLFC_00990 | Protein FdrA | . | . | NA | NA | K02381 | 455.1 | 672 | 4.90E-204 | FdrA protein | K02381 | 455.1 | 671.5 | 7.30E-204 | FdrA protein | NA | NA | NA | NA | NA | NA | NA | NA | NA | NA | NA |
| LBHGKGNI_00619 | LHADMLFC_00993 | Carbamate kinase 1 | . | Yes | NA | arcC1 Carbamate kinase 1 1075112:1076014 forward MW:31902 | K00926 | 120.7 | 394.5 | 4.30E-120 | carbamate kinase [EC:2.7.2.2] | K00926 | 120.7 | 392.7 | 1.60E-119 | carbamate kinase [EC:2.7.2.2] | NA | NA | NA | NA | NA | NA | NA | NA | NA | NA | NA |
| LBHGKGNI_00703 | LHADMLFC_01013 | Toluene efflux pump outer membrane protein TtgI | Yes | Yes | ttgI Toluene efflux pump outer membrane protein TtgI 717713:719128 forward MW:51963 | ttgI Toluene efflux pump outer membrane protein TtgI 1092839:1094254 forward MW:51949 | . | . | . | . | . | . | . | . | . | . | NA | NA | NA | NA | NA | NA | NA | NA | NA | NA | NA |
| LBHGKGNI_00705 | LHADMLFC_01015 | putative ABC transporter ATP-binding protein | Yes | Yes | LBHGKGNI_00705 putative ABC transporter ATP-binding protein 720282:721007 forward MW:26117 | LHADMLFC_01015 putative ABC transporter ATP-binding protein 1095408:1096133 forward MW:26117 | K02003 | 287.77 | 295.3 | 5.50E-90 | putative ABC transport system ATP-binding protein | K02003 | 287.77 | 295.3 | 6.00E-90 | putative ABC transport system ATP-binding protein | NA | NA | NA | NA | NA | NA | NA | NA | NA | NA | NA |
| LBHGKGNI_00706 | LHADMLFC_01016 | p-hydroxybenzoic acid efflux pump subunit AaeA | Yes | Yes | aaeA_1 p-hydroxybenzoic acid efflux pump subunit AaeA 721018:721926 forward MW:32507 | aaeA_1 p-hydroxybenzoic acid efflux pump subunit AaeA 1096144:1097052 forward MW:32477 | . | . | . | . | . | . | . | . | . | . | NA | NA | NA | NA | NA | NA | NA | NA | NA | NA | NA |
| LBHGKGNI_00707 | LHADMLFC_01017 | 2-hydroxy-3-keto-5-methylthiopentenyl-1-phosphate phosphatase | Yes | Yes | LBHGKGNI_00707 hypothetical protein 721958:722173 forward MW:7948 | mtnX 2-hydroxy-3-keto-5-methylthiopentenyl-1- phosphate phosphatase 1097084:1097785 forward MW:26249 | . | . | . | . | . | K08966 | 122.33 | 252.6 | 2.80E-77 | 2-hydroxy-3-keto-5-methylthiopentenyl-1-phosphate phosphatase [EC:3.1.3.87] | NA | NA | NA | NA | NA | NA | NA | NA | NA | NA | NA |
| LBHGKGNI_00710 | LHADMLFC_01018 | Thermostable monoacylglycerol lipase | Yes | Yes | LBHGKGNI_00710 Thermostable monoacylglycerol lipase 723722:724612 forward MW:32645 | LHADMLFC_01018 Thermostable monoacylglycerol lipase 1097782:1098672 forward MW:32645 | K03928 | 128.23 | 241.8 | 7.80E-74 | carboxylesterase [EC:3.1.1.1] | K03928 | 128.23 | 241.8 | 8.40E-74 | carboxylesterase [EC:3.1.1.1] | NA | NA | NA | NA | NA | NA | NA | NA | NA | NA | NA |
| LBHGKGNI_00711 | LHADMLFC_01019 | 4-amino-4-deoxy-L-arabinose-phosphoundecaprenol flippase subunit ArnE | Yes | Yes | arnE_1 4-amino-4-deoxy-L-arabinose-phosphoundecaprenol flippase subunit ArnE 724609:724968 forward MW:12901 | arnE_1 4-amino-4-deoxy-L-arabinose-phosphoundecaprenol flippase subunit ArnE 1098669:1099028 forward MW:12901 | . | . | . | . | . | . | . | . | . | . | NA | NA | NA | NA | NA | NA | NA | NA | NA | NA | NA |
| LBHGKGNI_00714 | LHADMLFC_01022 | 5-aminovalerate aminotransferase DavT | Yes | Yes | davT 5-aminovalerate aminotransferase DavT 727493:728869 reverse MW:51470 | davT 5-aminovalerate aminotransferase DavT 1101553:1102929 reverse MW:51470 | . | . | . | . | . | . | . | . | . | . | NA | NA | NA | NA | NA | NA | NA | NA | NA | NA | NA |
| LBHGKGNI_00716 | LHADMLFC_01025 | Sensor histidine kinase RcsC | Yes | Yes | sasA_2 Adaptive-response sensory-kinase SasA 729178:730236 reverse MW:39139 | rcsC_2 Sensor histidine kinase RcsC 1104483:1105496 reverse MW:37727 | K18143 | 344.37 | 389.8 | 7.60E-119 | two-component system, OmpR family, sensor histidine kinase AdeS [EC:2.7.13.3] | K18143 | 344.37 | 356.3 | 1.30E-108 | two-component system, OmpR family, sensor histidine kinase AdeS [EC:2.7.13.3] | NA | NA | NA | NA | NA | NA | NA | NA | NA | NA | NA |
| LBHGKGNI_00717 | LHADMLFC_01026 | Phosphate regulon transcriptional regulatory protein PhoB | Yes | Yes | phoB_2 Phosphate regulon transcriptional regulatory protein PhoB 730238:730933 reverse MW:26535 | phoB_2 Phosphate regulon transcriptional regulatory protein PhoB 1105498:1106193 reverse MW:26535 | K18144 | 262.03 | 309.8 | 7.80E-95 | two-component system, OmpR family, response regulator AdeR | K18144 | 262.03 | 309.8 | 8.50E-95 | two-component system, OmpR family, response regulator AdeR | NA | NA | NA | NA | NA | NA | NA | NA | NA | NA | NA |
| LBHGKGNI_00721 | LHADMLFC_01029 | Aclacinomycin methylesterase RdmC | Yes | Yes | rdmC_1 Aclacinomycin methylesterase RdmC 736300:737175 reverse MW:32104 | rdmC_1 Aclacinomycin methylesterase RdmC 1111558:1112433 reverse MW:32119 | . | . | . | . | . | . | . | . | . | . | NA | NA | NA | NA | NA | NA | NA | NA | NA | NA | NA |
| LBHGKGNI_00722 | LHADMLFC_01030 | Baeyer-Villiger monooxygenase | Yes | Yes | LBHGKGNI_00722 Baeyer-Villiger monooxygenase 737172:738698 reverse MW:57469 | LHADMLFC_01030 Baeyer-Villiger monooxygenase 1112430:1113956 reverse MW:57469 | K10215 | 383.8 | 735.8 | 2.10E-223 | monooxygenase [EC:1.14.13.-] | K10215 | 383.8 | 735.8 | 2.30E-223 | monooxygenase [EC:1.14.13.-] | NA | NA | NA | NA | NA | NA | NA | NA | NA | NA | NA |
| LBHGKGNI_00723 | LHADMLFC_01033 | HTH-type transcriptional regulator BetI | Yes | Yes | betI_1 HTH-type transcriptional regulator BetI 739011:739604 reverse MW:21953 | betI_1 HTH-type transcriptional regulator BetI 1115494:1116087 reverse MW:21953 | K23778 | 108.57 | 109.2 | 2.30E-33 | TetR/AcrR family transcriptional regulator, regulator of biofilm formation and stress response | K23778 | 108.57 | 109.2 | 2.50E-33 | TetR/AcrR family transcriptional regulator, regulator of biofilm formation and stress response | NA | NA | NA | NA | NA | NA | NA | NA | NA | NA | NA |
| LBHGKGNI_00726 | LHADMLFC_01036 | Gamma-glutamylputrescine oxidoreductase | Yes | Yes | puuB_1 Gamma-glutamylputrescine oxidoreductase 742514:743881 forward MW:48838 | puuB_2 Gamma-glutamylputrescine oxidoreductase 1118997:1120364 forward MW:48838 | K09471 | 196.87 | 351.1 | 6.80E-107 | gamma-glutamylputrescine oxidase [EC:1.4.3.-] | K09471 | 196.87 | 351.1 | 7.40E-107 | gamma-glutamylputrescine oxidase [EC:1.4.3.-] | NA | NA | NA | NA | NA | NA | NA | NA | NA | NA | NA |
| LBHGKGNI_00731 | LHADMLFC_01041 | 1-deoxyxylulose-5-phosphate synthase YajO | . | . | NA | NA | K23107 | 337.13 | 349.7 | 1.40E-106 | 1-deoxyxylulose-5-phosphate synthase [EC:1.1.-.-] | K23107 | 337.13 | 349.7 | 1.50E-106 | 1-deoxyxylulose-5-phosphate synthase [EC:1.1.-.-] | NA | NA | NA | NA | NA | NA | NA | NA | NA | NA | NA |
| LBHGKGNI_00732 | LHADMLFC_01042 | HTH-type transcriptional regulator PgrR | . | . | NA | NA | K03566 | 201.83 | 207.8 | 2.10E-63 | LysR family transcriptional regulator, glycine cleavage system transcriptional activator | K03566 | 201.83 | 207.8 | 2.20E-63 | LysR family transcriptional regulator, glycine cleavage system transcriptional activator | NA | NA | NA | NA | NA | NA | NA | NA | NA | NA | NA |
| LBHGKGNI_00754 | LHADMLFC_01065 | Putative O-antigen transporter | . | . | NA | NA | . | . | . | . | . | . | . | . | . | . | NA | NA | NA | NA | NA | NA | NA | NA | NA | NA | NA |
| LBHGKGNI_00980 | LHADMLFC_01289 | 2-(hydroxymethyl)glutarate dehydrogenase | . | . | NA | NA | . | . | . | . | . | K00020 | 225.47 | 251.5 | 1.40E-76 | 3-hydroxyisobutyrate dehydrogenase [EC:1.1.1.31] | NA | NA | NA | NA | NA | NA | NA | NA | NA | NA | NA |
| LBHGKGNI_01087 | LHADMLFC_01394 | Common pilus major fimbrillin subunit EcpA | . | . | NA | NA | K21964 | 82.2 | 273 | 1.10E-83 | Mat/Ecp fimbriae major subunit | K21964 | 82.2 | 273.7 | 7.30E-84 | Mat/Ecp fimbriae major subunit | NA | NA | NA | NA | NA | NA | NA | NA | NA | NA | NA |
| LBHGKGNI_01088 | LHADMLFC_01395 | putative fimbrial chaperone EcpB | . | . | NA | NA | K21965 | 149.5 | 238.3 | 4.90E-73 | Mat/Ecp fimbriae periplasmic chaperone | K21965 | 149.5 | 238.3 | 5.40E-73 | Mat/Ecp fimbriae periplasmic chaperone | NA | NA | NA | NA | NA | NA | NA | NA | NA | NA | NA |
| LBHGKGNI_01089 | LHADMLFC_01396 | putative outer membrane usher protein EcpC | . | . | NA | NA | K21966 | 207.1 | 735.4 | 9.90E-223 | Mat/Ecp fimbriae outer membrane usher protein | K21966 | 207.1 | 734.7 | 1.70E-222 | Mat/Ecp fimbriae outer membrane usher protein | NA | NA | NA | NA | NA | NA | NA | NA | NA | NA | NA |
| LBHGKGNI_01090 | LHADMLFC_01397 | Fimbria adhesin EcpD | . | . | NA | NA | K21967 | 170.9 | 804.2 | 3.20E-244 | Mat/Ecp fimbriae adhesin | K21967 | 170.9 | 807.2 | 4.30E-245 | Mat/Ecp fimbriae adhesin | NA | NA | NA | NA | NA | NA | NA | NA | NA | NA | NA |
| LBHGKGNI_01091 | LHADMLFC_01398 | putative fimbrial chaperone EcpE | . | . | NA | NA | K21968 | 192 | 282.4 | 1.20E-86 | Mat/Ecp fimbriae periplasmic chaperone | K21968 | 192 | 282.4 | 1.30E-86 | Mat/Ecp fimbriae periplasmic chaperone | NA | NA | NA | NA | NA | NA | NA | NA | NA | NA | NA |
| LBHGKGNI_01128 | LHADMLFC_01436 | Proline/betaine transporter | . | . | NA | NA | K03762 | 472.83 | 482.5 | 1.50E-146 | MFS transporter, MHS family, proline/betaine transporter | K03762 | 472.83 | 484.6 | 3.80E-147 | MFS transporter, MHS family, proline/betaine transporter | NA | NA | NA | NA | NA | NA | NA | NA | NA | NA | NA |
| LBHGKGNI_01179 | LHADMLFC_01489 | D-inositol-3-phosphate glycosyltransferase | Yes | Yes | mshA_1 D-inositol-3-phosphate glycosyltransferase 1198790:1199833 forward MW:39351 | mshA_1 D-inositol-3-phosphate glycosyltransferase 1575040:1576083 forward MW:39252 | . | . | . | . | . | . | . | . | . | . | NA | NA | NA | NA | NA | NA | NA | NA | NA | NA | NA |
| LBHGKGNI_01180 | LHADMLFC_01490 | D-inositol-3-phosphate glycosyltransferase | Yes | Yes | mshA_2 D-inositol-3-phosphate glycosyltransferase 1199908:1201065 forward MW:43736 | mshA_2 D-inositol-3-phosphate glycosyltransferase 1576158:1577315 forward MW:43736 | . | . | . | . | . | . | . | . | . | . | NA | NA | NA | NA | NA | NA | NA | NA | NA | NA | NA |
| LBHGKGNI_01190 | LHADMLFC_01500 | dTDP-4-dehydrorhamnose 3,5-epimerase | . | . | NA | NA | K01790 | 199.8 | 298.1 | 4.20E-91 | dTDP-4-dehydrorhamnose 3,5-epimerase [EC:5.1.3.13] | K01790 | 199.8 | 298.1 | 4.60E-91 | dTDP-4-dehydrorhamnose 3,5-epimerase [EC:5.1.3.13] | NA | NA | NA | NA | NA | NA | NA | NA | NA | NA | NA |
| LBHGKGNI_01191 | LHADMLFC_01501 | dTDP-4-dehydrorhamnose reductase | . | . | NA | NA | K00067 | 183.07 | 385.8 | 1.90E-117 | dTDP-4-dehydrorhamnose reductase [EC:1.1.1.133] | K00067 | 183.07 | 385.8 | 2.10E-117 | dTDP-4-dehydrorhamnose reductase [EC:1.1.1.133] | NA | NA | NA | NA | NA | NA | NA | NA | NA | NA | NA |
| LBHGKGNI_01192 | LHADMLFC_01502 | Putative O-antigen transporter | . | . | NA | NA | K18799 | 205.83 | 209.7 | 6.30E-64 | O-antigen flippase | K18799 | 205.83 | 209.7 | 6.80E-64 | O-antigen flippase | NA | NA | NA | NA | NA | NA | NA | NA | NA | NA | NA |
| LBHGKGNI_01197 | LHADMLFC_01507 | Mannose-1-phosphate guanylyltransferase RfbM | . | . | NA | NA | K00971 | 112.37 | 452 | 2.30E-137 | mannose-1-phosphate guanylyltransferase [EC:2.7.7.13] | K00971 | 112.37 | 451.5 | 3.50E-137 | mannose-1-phosphate guanylyltransferase [EC:2.7.7.13] | NA | NA | NA | NA | NA | NA | NA | NA | NA | NA | NA |
| LBHGKGNI_01198 | LHADMLFC_01508 | Phosphomannomutase/phosphoglucomutase | . | . | NA | NA | K01840 | 452.37 | 505 | 3.50E-153 | phosphomannomutase [EC:5.4.2.8] | K01840 | 452.37 | 499.8 | 1.40E-151 | phosphomannomutase [EC:5.4.2.8] | NA | NA | NA | NA | NA | NA | NA | NA | NA | NA | NA |
| LBHGKGNI_01243 | LHADMLFC_01543 | 5'-methylthioadenosine/S-adenosylhomocysteine nucleosidase | . | . | NA | NA | K01243 | 109.37 | 171.9 | 1.90E-52 | adenosylhomocysteine nucleosidase [EC:3.2.2.9] | K01243 | 109.37 | 171.9 | 2.10E-52 | adenosylhomocysteine nucleosidase [EC:3.2.2.9] | NA | NA | NA | NA | NA | NA | NA | NA | NA | NA | NA |
| LBHGKGNI_01245 | LHADMLFC_01545 | HTH-type transcriptional regulator TrpI | . | . | NA | NA | K03566 | 201.83 | 263 | 3.50E-80 | LysR family transcriptional regulator, glycine cleavage system transcriptional activator | K03566 | 201.83 | 263.3 | 2.90E-80 | LysR family transcriptional regulator, glycine cleavage system transcriptional activator | NA | NA | NA | NA | NA | NA | NA | NA | NA | NA | NA |
| LBHGKGNI_01246 | LHADMLFC_01546 | D-threonate kinase | . | . | NA | NA | K22129 | 273.9 | 341.4 | 6.80E-104 | D-threonate/D-erythronate kinase [EC:2.7.1.219 2.7.1.220] | K22129 | 273.9 | 339.6 | 2.50E-103 | D-threonate/D-erythronate kinase [EC:2.7.1.219 2.7.1.220] | NA | NA | NA | NA | NA | NA | NA | NA | NA | NA | NA |
| LBHGKGNI_01247 | LHADMLFC_01547 | D-threonate 4-phosphate dehydrogenase | Yes | . | pdxA2 D-threonate 4-phosphate dehydrogenase 1273845:1274846 forward MW:35100 | NA | K00097 | 412.37 | 453.7 | 6.00E-138 | 4-hydroxythreonine-4-phosphate dehydrogenase [EC:1.1.1.262] | K00097 | 412.37 | 453.7 | 6.50E-138 | 4-hydroxythreonine-4-phosphate dehydrogenase [EC:1.1.1.262] | NA | NA | NA | NA | NA | NA | NA | NA | NA | NA | NA |
| LBHGKGNI_01416 | LHADMLFC_01681 | Transcriptional regulatory protein RcsB | . | . | NA | NA | . | . | . | . | . | . | . | . | . | . | NA | NA | NA | NA | NA | NA | NA | NA | NA | NA | NA |
| LBHGKGNI_01418 | LHADMLFC_01683 | putative fimbrial chaperone YehC | . | . | NA | NA | K15540 | 260.83 | 262.6 | 2.50E-80 | chaperone protein EcpD | K15540 | 260.83 | 262.3 | 3.40E-80 | chaperone protein EcpD | NA | NA | NA | NA | NA | NA | NA | NA | NA | NA | NA |
| LBHGKGNI_01419 | LHADMLFC_01684 | Outer membrane usher protein YehB | . | . | NA | NA | K07347 | 607.23 | 745.7 | 7.60E-226 | outer membrane usher protein | K07347 | 607.23 | 748.5 | 1.10E-226 | outer membrane usher protein | NA | NA | NA | NA | NA | NA | NA | NA | NA | NA | NA |
| LBHGKGNI_01494 | LHADMLFC_01874 | Type IV secretion system protein VirB11 | Yes | Yes | LBHGKGNI_01494 Type IV secretion system protein VirB11 1530010:1531044 reverse MW:36740 | LHADMLFC_01874 hypothetical protein 1989786:1990856 forward MW:37710 | K20527 | 301.23 | 512.9 | 4.00E-156 | type IV secretion system protein TrbB [EC:7.4.2.8] | K20527 | 301.23 | 522 | 7.50E-159 | type IV secretion system protein TrbB [EC:7.4.2.8] | NA | NA | NA | NA | NA | NA | NA | NA | NA | NA | NA |
| LBHGKGNI_01562 | LHADMLFC_01770 | Citrate lyase subunit beta-like protein | Yes | . | LBHGKGNI_01562 Citrate lyase subunit beta-like protein 1597870:1598694 reverse MW:30267 | NA | K01644 | 156 | 270.4 | 2.70E-82 | citrate lyase subunit beta / citryl-CoA lyase [EC:4.1.3.34] | K01644 | 156 | 270.4 | 2.90E-82 | citrate lyase subunit beta / citryl-CoA lyase [EC:4.1.3.34] | NA | NA | NA | NA | NA | NA | NA | NA | NA | NA | NA |
| LBHGKGNI_01565 | LHADMLFC_01772 | Ferric-anguibactin receptor FatA | Yes | Yes | fatA Ferric-anguibactin receptor FatA 1602177:1603976 forward MW:64771 | fatA Ferric-anguibactin receptor FatA 1883908:1886019 forward MW:76063 | K02014 | 166.57 | 212.5 | 1.00E-64 | iron complex outermembrane recepter protein | K02014 | 166.57 | 249.3 | 8.20E-76 | iron complex outermembrane recepter protein | NA | NA | NA | NA | NA | NA | NA | NA | NA | NA | NA |
| LBHGKGNI_01566 | LHADMLFC_01773 | Diaminopimelate decarboxylase | Yes | Yes | lysA_1 Diaminopimelate decarboxylase 1603988:1605388 forward MW:51574 | lysA_1 Diaminopimelate decarboxylase 1886031:1887431 forward MW:51575 | . | . | . | . | . | . | . | . | . | . | NA | NA | NA | NA | NA | NA | NA | NA | NA | NA | NA |
| LBHGKGNI_01567 | LHADMLFC_01774 | Cysteine synthase | Yes | Yes | cysK_1 Cysteine synthase 1605400:1606413 forward MW:36059 | cysK_1 Cysteine synthase 1887443:1888456 forward MW:36041 | . | . | . | . | . | . | . | . | . | . | NA | NA | NA | NA | NA | NA | NA | NA | NA | NA | NA |
| LBHGKGNI_01569 | LHADMLFC_01776 | Staphyloferrin B transporter | Yes | Yes | sbnD_2 Staphyloferrin B transporter 1607573:1608799 forward MW:43925 | sbnD_2 Staphyloferrin B transporter 1889616:1890842 forward MW:43869 | K23361 | 307.77 | 357.2 | 7.10E-109 | MFS transporter, DHA1 family, staphyloferrin B biosynthesis exporter | K23361 | 307.77 | 355.9 | 1.90E-108 | MFS transporter, DHA1 family, staphyloferrin B biosynthesis exporter | NA | NA | NA | NA | NA | NA | NA | NA | NA | NA | NA |
| LBHGKGNI_01571 | LHADMLFC_01778 | putative cysteine desulfurase | Yes | Yes | csd putative cysteine desulfurase 1609925:1611109 forward MW:43744 | csd putative cysteine desulfurase 1891968:1893152 forward MW:43730 | . | . | . | . | . | . | . | . | . | . | NA | NA | NA | NA | NA | NA | NA | NA | NA | NA | NA |
| LBHGKGNI_01658 | LHADMLFC_01908 | L-2-hydroxyglutarate dehydrogenase | . | . | NA | NA | K15736 | 576.8 | 640.7 | 2.50E-194 | (S)-2-hydroxyglutarate dehydrogenase [EC:1.1.5.13] | K15736 | 576.8 | 641.7 | 1.40E-194 | (S)-2-hydroxyglutarate dehydrogenase [EC:1.1.5.13] | NA | NA | NA | NA | NA | NA | NA | NA | NA | NA | NA |
| LBHGKGNI_01659 | LHADMLFC_01909 | HTH-type transcriptional regulator LutR | . | . | NA | NA | K05799 | 138.07 | 202.3 | 1.20E-61 | GntR family transcriptional regulator, transcriptional repressor for pyruvate dehydrogenase complex | K05799 | 138.07 | 203.2 | 6.90E-62 | GntR family transcriptional regulator, transcriptional repressor for pyruvate dehydrogenase complex | NA | NA | NA | NA | NA | NA | NA | NA | NA | NA | NA |
| LBHGKGNI_01702 | LHADMLFC_01951 | L-threo-3-hydroxyaspartate ammonia-lyase | . | . | NA | NA | K22589 | 432.47 | 527.9 | 1.10E-160 | threo-3-hydroxy-L-aspartate ammonia-lyase [EC:4.3.1.16] | K22589 | 432.47 | 526 | 4.30E-160 | threo-3-hydroxy-L-aspartate ammonia-lyase [EC:4.3.1.16] | NA | NA | NA | NA | NA | NA | NA | NA | NA | NA | NA |
| LBHGKGNI_01703 | LHADMLFC_01952 | Transcriptional regulator DauR | . | . | NA | NA | . | . | . | . | . | . | . | . | . | . | NA | NA | NA | NA | NA | NA | NA | NA | NA | NA | NA |
| LBHGKGNI_01995 | LHADMLFC_02210 | Catechol O-methyltransferase | . | . | NA | NA | K00588 | 110.2 | 154.5 | 2.70E-47 | caffeoyl-CoA O-methyltransferase [EC:2.1.1.104] | K00588 | 110.2 | 155.5 | 1.50E-47 | caffeoyl-CoA O-methyltransferase [EC:2.1.1.104] | NA | NA | NA | NA | NA | NA | NA | NA | NA | NA | NA |
| LBHGKGNI_02028 | LHADMLFC_02243 | Thiol-disulfide oxidoreductase ResA | . | . | NA | NA | . | . | . | . | . | . | . | . | . | . | NA | NA | NA | NA | NA | NA | NA | NA | NA | NA | NA |
| LBHGKGNI_02029 | LHADMLFC_02244 | HTH-type transcriptional regulator ArgP | . | . | NA | NA | . | . | . | . | . | . | . | . | . | . | NA | NA | NA | NA | NA | NA | NA | NA | NA | NA | NA |
| LBHGKGNI_02076 | LHADMLFC_02291 | HTH-type transcriptional regulator PgrR | . | . | NA | NA | . | . | . | . | . | . | . | . | . | . | NA | NA | NA | NA | NA | NA | NA | NA | NA | NA | NA |
| LBHGKGNI_02077 | LHADMLFC_02292 | putative protein YcjY | . | . | NA | NA | . | . | . | . | . | . | . | . | . | . | NA | NA | NA | NA | NA | NA | NA | NA | NA | NA | NA |
| LBHGKGNI_02127 | LHADMLFC_02335 | Aurachin B dehydrogenase | . | . | NA | NA | . | . | . | . | . | . | . | . | . | . | NA | NA | NA | NA | NA | NA | NA | NA | NA | NA | NA |
| LBHGKGNI_02163 | LHADMLFC_02389 | Maltodextrin phosphorylase | . | . | NA | NA | K00688 | 72.7 | 1086.4 | 0 | glycogen phosphorylase [EC:2.4.1.1] | K00688 | 72.7 | 1086.2 | 0 | glycogen phosphorylase [EC:2.4.1.1] | NA | NA | NA | NA | NA | NA | NA | NA | NA | NA | NA |
| LBHGKGNI_02165 | LHADMLFC_02391 | Regulator of RpoS | . | . | NA | NA | K07315 | 146.03 | 248.8 | 7.80E-76 | phosphoserine phosphatase RsbU/P [EC:3.1.3.3] | K07315 | 146.03 | 248.6 | 9.50E-76 | phosphoserine phosphatase RsbU/P [EC:3.1.3.3] | NA | NA | NA | NA | NA | NA | NA | NA | NA | NA | NA |
| LBHGKGNI_02166 | LHADMLFC_02392 | Putative anti-sigma factor antagonist | . | . | NA | NA | K04749 | 64.6 | 96.5 | 1.80E-29 | anti-sigma B factor antagonist | K04749 | 64.6 | 96.5 | 2.00E-29 | anti-sigma B factor antagonist | NA | NA | NA | NA | NA | NA | NA | NA | NA | NA | NA |
| LBHGKGNI_02167 | LHADMLFC_02393 | Serine-protein kinase RsbW | . | . | NA | NA | K04757 | 51.53 | 90.4 | 1.20E-27 | serine/threonine-protein kinase RsbW [EC:2.7.11.1] | K04757 | 51.53 | 88.2 | 5.70E-27 | serine/threonine-protein kinase RsbW [EC:2.7.11.1] | NA | NA | NA | NA | NA | NA | NA | NA | NA | NA | NA |
| LBHGKGNI_02170 | LHADMLFC_02396 | Beta-galactosidase | Yes | . | lacZ_1 Beta-galactosidase 2202077:2204260 reverse MW:82618 | NA | K01192 | 399.33 | 410 | 2.10E-124 | beta-mannosidase [EC:3.2.1.25] | K01192 | 399.33 | 410.2 | 1.90E-124 | beta-mannosidase [EC:3.2.1.25] | NA | NA | NA | NA | NA | NA | NA | NA | NA | NA | NA |
| LBHGKGNI_02171 | LHADMLFC_02397 | Sensor histidine kinase RcsC | Yes | . | rcsC_2 Sensor histidine kinase RcsC 2204257:2206938 reverse MW:98296 | NA | K11527 | 632.93 | 693.8 | 2.30E-210 | two-component system, sensor histidine kinase and response regulator [EC:2.7.13.3] | K11527 | 632.93 | 697.4 | 2.00E-211 | two-component system, sensor histidine kinase and response regulator [EC:2.7.13.3] | NA | NA | NA | NA | NA | NA | NA | NA | NA | NA | NA |
| LBHGKGNI_02176 | LHADMLFC_02402 | Cellulose synthase 1 | Yes | . | acsAB Cellulose synthase 1 2211705:2213543 reverse MW:69825 | NA | K00694 | 279.13 | 483.9 | 5.80E-147 | cellulose synthase (UDP-forming) [EC:2.4.1.12] | K00694 | 279.13 | 484.9 | 3.20E-147 | cellulose synthase (UDP-forming) [EC:2.4.1.12] | NA | NA | NA | NA | NA | NA | NA | NA | NA | NA | NA |
| LBHGKGNI_02261 | LHADMLFC_02508 | HTH-type transcriptional regulator BenM | . | . | NA | NA | . | . | . | . | . | . | . | . | . | . | NA | NA | NA | NA | NA | NA | NA | NA | NA | NA | NA |
| LBHGKGNI_02266 | LHADMLFC_02513 | Vitamin B12 import ATP-binding protein BtuD | . | . | NA | NA | K02049 | 352.6 | 378 | 6.10E-115 | NitT/TauT family transport system ATP-binding protein | K02049 | 352.6 | 370.2 | 1.50E-112 | NitT/TauT family transport system ATP-binding protein | NA | NA | NA | NA | NA | NA | NA | NA | NA | NA | NA |
| LBHGKGNI_02382 | LHADMLFC_02620 | 16S rRNA endonuclease CdiA | Yes | . | cdiA_2 tRNA nuclease CdiA 2438072:2438878 reverse MW:27994 | NA | . | . | . | . | . | . | . | . | . | . | NA | NA | NA | NA | NA | NA | NA | NA | NA | NA | NA |
| LBHGKGNI_02396 | LHADMLFC_02626 | Toxin CdiA | . | Yes | NA | cdiA_4 Toxin CdiA 2768766:2769392 reverse MW:20283 | . | . | . | . | . | . | . | . | . | . | NA | NA | NA | NA | NA | NA | NA | NA | NA | NA | NA |
| LBHGKGNI_02730 | LHADMLFC_03005 | Lipoprotein E | . | Yes | NA | hel_2 Lipoprotein E 3161704:3162519 reverse MW:31113 | . | . | . | . | . | . | . | . | . | . | NA | NA | NA | NA | NA | NA | NA | NA | NA | NA | NA |
| LBHGKGNI_02734 | LHADMLFC_03009 | tRNA(fMet)-specific endonuclease VapC | . | Yes | NA | vapC_1 tRNA(fMet)-specific endonuclease VapC 3166601:3166999 reverse MW:14930 | K18828 | 103.9 | 157.8 | 2.50E-48 | tRNA(fMet)-specific endonuclease VapC [EC:3.1.-.-] | K18828 | 103.9 | 156.6 | 6.60E-48 | tRNA(fMet)-specific endonuclease VapC [EC:3.1.-.-] | NA | NA | NA | NA | NA | NA | NA | NA | NA | NA | NA |
| LBHGKGNI_02735 | LHADMLFC_03010 | Antitoxin VapB2 | . | Yes | NA | vapB2 Antitoxin VapB2 3166999:3167232 reverse MW:8841 | K18829 | 38.57 | 76.1 | 2.20E-23 | antitoxin VapB | K18829 | 38.57 | 76.1 | 2.40E-23 | antitoxin VapB | NA | NA | NA | NA | NA | NA | NA | NA | NA | NA | NA |
| LBHGKGNI_02809 | LHADMLFC_03100 | Protein UmuC | . | Yes | NA | umuC_3 Protein UmuC 3285799:3286017 reverse MW:7954 | . | . | . | . | . | . | . | . | . | . | NA | NA | NA | NA | NA | NA | NA | NA | NA | NA | NA |
| LBHGKGNI_03021 | LHADMLFC_03313 | HTH-type transcriptional regulator TsaR | . | . | NA | NA | K14057 | 267.67 | 360.2 | 6.30E-110 | LysR family transcriptional regulator, regulator of abg operon | K14057 | 267.67 | 360.7 | 5.10E-110 | LysR family transcriptional regulator, regulator of abg operon | NA | NA | NA | NA | NA | NA | NA | NA | NA | NA | NA |
| LBHGKGNI_03022 | LHADMLFC_03314 | Hippurate hydrolase | . | . | NA | NA | K01451 | 497.7 | 690.2 | 2.30E-209 | hippurate hydrolase [EC:3.5.1.32] | K01451 | 497.7 | 683.2 | 3.20E-207 | hippurate hydrolase [EC:3.5.1.32] | NA | NA | NA | NA | NA | NA | NA | NA | NA | NA | NA |
| LBHGKGNI_03023 | LHADMLFC_03315 | Lysine/arginine/ornithine-binding periplasmic protein | . | . | NA | NA | K02030 | 86.17 | 161.3 | 2.60E-49 | polar amino acid transport system substrate-binding protein | K02030 | 86.17 | 161.3 | 2.80E-49 | polar amino acid transport system substrate-binding protein | NA | NA | NA | NA | NA | NA | NA | NA | NA | NA | NA |
| LBHGKGNI_03100 | LHADMLFC_03386 | Outer membrane porin C | . | . | NA | NA | . | . | . | . | . | K09475 | 575.27 | 599.1 | 2.10E-182 | outer membrane pore protein C | BAC0529\|kpnO\|tr\|C4XBC3\|C4XBC3_KLEPN | 63.467 | 323 | 101 | 4 | 1 | 320 | 57 | 365 | 1.37E-138 | 392 |
| LBHGKGNI_03275 | LHADMLFC_03560 | putative major fimbrial subunit LpfA | Yes | Yes | lpfA_4 putative major fimbrial subunit LpfA 3397209:3397745 forward MW:18392 | lpfA_3 putative major fimbrial subunit LpfA 3783974:3784510 forward MW:18392 | K07345 | 89.47 | 131.8 | 2.80E-40 | major type 1 subunit fimbrin (pilin) | K07345 | 89.47 | 131.8 | 3.00E-40 | major type 1 subunit fimbrin (pilin) | NA | NA | NA | NA | NA | NA | NA | NA | NA | NA | NA |
| LBHGKGNI_03276 | LHADMLFC_03561 | putative fimbrial chaperone YraI | Yes | Yes | yraI putative fimbrial chaperone YraI 3397815:3398495 forward MW:25592 | yraI putative fimbrial chaperone YraI 3784580:3785260 forward MW:25569 | K07346 | 171 | 188 | 1.80E-57 | fimbrial chaperone protein | K07346 | 171 | 188.8 | 1.00E-57 | fimbrial chaperone protein | NA | NA | NA | NA | NA | NA | NA | NA | NA | NA | NA |
| LBHGKGNI_03277 | LHADMLFC_03562 | putative outer membrane usher protein ElfC | Yes | Yes | elfC putative outer membrane usher protein ElfC 3398517:3400985 forward MW:91172 | elfC_1 putative outer membrane usher protein ElfC 3785282:3787750 forward MW:91205 | K07347 | 607.23 | 792.4 | 5.70E-240 | outer membrane usher protein | K07347 | 607.23 | 792 | 7.90E-240 | outer membrane usher protein | NA | NA | NA | NA | NA | NA | NA | NA | NA | NA | NA |
| LBHGKGNI_03370 | LHADMLFC_03653 | Molybdenum-pterin-binding protein MopA | . | . | NA | NA | K02019 | 69.03 | 147 | 5.10E-45 | molybdate transport system regulatory protein | K02019 | 69.03 | 144.9 | 2.30E-44 | molybdate transport system regulatory protein | NA | NA | NA | NA | NA | NA | NA | NA | NA | NA | NA |
| LBHGKGNI_03829 | LHADMLFC_03991 | Ribonuclease toxin YhaV | Yes | Yes | yhaV Ribonuclease toxin YhaV 3985167:3985631 reverse MW:17643 | yhaV Ribonuclease toxin YhaV 4239402:4239866 reverse MW:17643 | K19155 | 63.87 | 221.6 | 7.70E-68 | toxin YhaV [EC:3.1.-.-] | K19155 | 63.87 | 221.6 | 8.30E-68 | toxin YhaV [EC:3.1.-.-] | NA | NA | NA | NA | NA | NA | NA | NA | NA | NA | NA |
| LBHGKGNI_04126 | LHADMLFC_04245 | HTH-type transcriptional regulator DmlR | . | . | NA | NA | . | . | . | . | . | . | . | . | . | . | NA | NA | NA | NA | NA | NA | NA | NA | NA | NA | NA |
| LBHGKGNI_04199 | LHADMLFC_04316 | Major phosphate-irrepressible acid phosphatase | . | . | NA | NA | K09474 | 130.7 | 221.1 | 1.60E-67 | acid phosphatase (class A) [EC:3.1.3.2] | K09474 | 130.7 | 221.1 | 1.70E-67 | acid phosphatase (class A) [EC:3.1.3.2] | NA | NA | NA | NA | NA | NA | NA | NA | NA | NA | NA |
| LBHGKGNI_04284 | LHADMLFC_04400 | Tyrocidine synthase 3 | . | . | NA | NA | K03367 | 559.97 | 597.2 | 1.80E-181 | D-alanine--poly(phosphoribitol) ligase subunit 1 [EC:6.1.1.13] | K22148 | 1278.13 | 1295.8 | 0 | ferricrocin synthase | NA | NA | NA | NA | NA | NA | NA | NA | NA | NA | NA |
| LBHGKGNI_04294 | LHADMLFC_04411 | Cyclic-di-GMP-binding biofilm dispersal mediator protein | . | . | NA | NA | K00059 | 273.2 | 288.9 | 5.60E-88 | 3-oxoacyl-[acyl-carrier protein] reductase [EC:1.1.1.100] | . | . | . | . | . | NA | NA | NA | NA | NA | NA | NA | NA | NA | NA | NA |
| LBHGKGNI_04446 | LHADMLFC_04634 | Phosphotriesterase homology protein | Yes | . | php Phosphotriesterase homology protein 4635925:4636818 reverse MW:33188 | NA | K07048 | 276.83 | 328.8 | 2.90E-100 | phosphotriesterase-related protein | K07048 | 276.83 | 328.8 | 3.20E-100 | phosphotriesterase-related protein | NA | NA | NA | NA | NA | NA | NA | NA | NA | NA | NA |
| LBHGKGNI_04447 | LHADMLFC_04633 | Phosphopentomutase | . | . | NA | NA | K01839 | 128.9 | 312.1 | 4.10E-95 | phosphopentomutase [EC:5.4.2.7] | K01839 | 128.9 | 312.1 | 4.50E-95 | phosphopentomutase [EC:5.4.2.7] | NA | NA | NA | NA | NA | NA | NA | NA | NA | NA | NA |
| LBHGKGNI_04448 | LHADMLFC_04632 | putative protein YhfX | . | . | NA | NA | . | . | . | . | . | . | . | . | . | . | NA | NA | NA | NA | NA | NA | NA | NA | NA | NA | NA |
| LBHGKGNI_04519 | LHADMLFC_02855 | Type IV secretion system protein virB4 | Yes | Yes | virB4_2 Type IV secretion system protein virB4 4723557:4726007 reverse MW:89461 | virB4_2 Type IV secretion system protein virB4 3007840:3010290 forward MW:89461 | K20530 | 499.83 | 1426.5 | 0 | type IV secretion system protein TrbE [EC:7.4.2.8] | K20530 | 499.83 | 1426.5 | 0 | type IV secretion system protein TrbE [EC:7.4.2.8] | NA | NA | NA | NA | NA | NA | NA | NA | NA | NA | NA |
| LBHGKGNI_04524 | LHADMLFC_02850 | Conjugal transfer protein TraG | Yes | Yes | traG_2 Conjugal transfer protein TraG 4728215:4730221 reverse MW:73153 | traG_2 Conjugal transfer protein TraG 3003626:3005632 forward MW:73153 | K03205 | 127.77 | 628.8 | 6.90E-191 | type IV secretion system protein VirD4 [EC:7.4.2.8] | K03205 | 127.77 | 628.8 | 7.50E-191 | type IV secretion system protein VirD4 [EC:7.4.2.8] | NA | NA | NA | NA | NA | NA | NA | NA | NA | NA | NA |
| LBHGKGNI_04526 | LHADMLFC_02848 | Hca operon transcriptional activator HcaR | Yes | Yes | hcaR_4 Hca operon transcriptional activator HcaR 4730717:4731661 reverse MW:34975 | hcaR_2 Hca operon transcriptional activator HcaR 3002186:3003130 forward MW:34975 | . | . | . | . | . | . | . | . | . | . | NA | NA | NA | NA | NA | NA | NA | NA | NA | NA | NA |
| LBHGKGNI_04527 | LHADMLFC_02847 | Methyl viologen resistance protein SmvA | Yes | Yes | smvA_2 Methyl viologen resistance protein SmvA 4731677:4733197 reverse MW:52264 | smvA_2 Methyl viologen resistance protein SmvA 3000650:3002170 forward MW:52264 | K08167 | 423.9 | 551.6 | 2.00E-167 | MFS transporter, DHA2 family, multidrug resistance protein | K08167 | 423.9 | 551.6 | 2.10E-167 | MFS transporter, DHA2 family, multidrug resistance protein | BAC0025\|amvA\|tr\|C4PAW9\|C4PAW9_ACIBA | 43.699 | 492 | 275 | 1 | 7 | 498 | 3 | 492 | 1.74E-141 | 412 |
| LBHGKGNI_04528 | LHADMLFC_02846 | Outer membrane protein OprJ | Yes | Yes | oprJ Outer membrane protein OprJ 4733261:4734745 reverse MW:53211 | oprJ Outer membrane protein OprJ 2999102:3000586 forward MW:53211 | K08721 | 647.3 | 787.2 | 3.50E-239 | outer membrane protein, multidrug efflux system | K08721 | 647.3 | 787.2 | 3.80E-239 | outer membrane protein, multidrug efflux system | BAC0291\|oprJ\|sp\|Q51397\|OPRJ_PSEAE | 61.845 | 477 | 176 | 2 | 10 | 484 | 2 | 474 | 0 | 546 |
| LBHGKGNI_04529 | LHADMLFC_02845 | Multidrug resistance protein MexB | Yes | Yes | mexB Multidrug resistance protein MexB 4734742:4737885 reverse MW:112538 | mexB Multidrug resistance protein MexB 2995962:2999105 forward MW:112552 | K18296 | 1730.2 | 1875.4 | 0 | multidrug efflux pump | K18296 | 1730.2 | 1875.5 | 0 | multidrug efflux pump | BAC0237\|mexD\|tr\|Q51396\|Q51396_PSEAI | 63.961 | 1035 | 364 | 5 | 1 | 1030 | 1 | 1031 | 0 | 1280 |
| LBHGKGNI_04530 | LHADMLFC_02844 | Toluene efflux pump periplasmic linker protein TtgG | Yes | Yes | ttgG Toluene efflux pump periplasmic linker protein TtgG 4737960:4739111 reverse MW:40709 | ttgG Toluene efflux pump periplasmic linker protein TtgG 2994736:2995887 forward MW:40709 | K18295 | 466.03 | 498.3 | 1.00E-151 | membrane fusion protein, multidrug efflux system | K18295 | 466.03 | 498.3 | 1.10E-151 | membrane fusion protein, multidrug efflux system | BAC0236\|mexC\|tr\|Q51395\|Q51395_PSEAI | 54.496 | 367 | 160 | 4 | 5 | 370 | 9 | 369 | 7.45E-131 | 376 |
| LBHGKGNI_04540 | LHADMLFC_02834 | Chromosome-partitioning ATPase Soj | Yes | Yes | soj_2 Chromosome-partitioning ATPase Soj 4745567:4746205 reverse MW:23001 | soj_2 Chromosome-partitioning ATPase Soj 2987642:2988280 forward MW:23001 | K03496 | 137.5 | 155.9 | 1.40E-47 | chromosome partitioning protein | K03496 | 137.5 | 155.9 | 1.50E-47 | chromosome partitioning protein | NA | NA | NA | NA | NA | NA | NA | NA | NA | NA | NA |
| LBHGKGNI_04552 | LHADMLFC_02822 | Putative nuclease YhcG | Yes | Yes | yhcG Putative nuclease YhcG 4758899:4759987 reverse MW:41291 | yhcG_2 Putative nuclease YhcG 2973860:2974948 forward MW:41291 | . | . | . | . | . | . | . | . | . | . | NA | NA | NA | NA | NA | NA | NA | NA | NA | NA | NA |
| LBHGKGNI_04853 | LHADMLFC_00554 | Toxin YjjJ | . | . | NA | NA | K07154 | 36.93 | 103.7 | 8.60E-32 | serine/threonine-protein kinase HipA [EC:2.7.11.1] | K07154 | 36.93 | 104.1 | 7.10E-32 | serine/threonine-protein kinase HipA [EC:2.7.11.1] | NA | NA | NA | NA | NA | NA | NA | NA | NA | NA | NA |
| LBHGKGNI_05176 | LHADMLFC_00417 | Linear gramicidin synthase subunit D | . | . | NA | NA | . | . | . | . | . | . | . | . | . | . | NA | NA | NA | NA | NA | NA | NA | NA | NA | NA | NA |
| LBHGKGNI_05198 | LHADMLFC_00434 | D-serine transporter DsdX | Yes | Yes | dsdX D-serine transporter DsdX 5486714:5488096 reverse MW:48476 | dsdX D-serine transporter DsdX 484203:485576 reverse MW:48270 | K03299 | 82.03 | 476.6 | 5.50E-145 | gluconate:H+ symporter, GntP family | K03299 | 82.03 | 449.9 | 7.50E-137 | gluconate:H+ symporter, GntP family | NA | NA | NA | NA | NA | NA | NA | NA | NA | NA | NA |
| LBHGKGNI_05199 | LHADMLFC_00435 | D-erythronate dehydrogenase | Yes | Yes | denD D-erythronate dehydrogenase 5488099:5489064 reverse MW:34155 | denD D-erythronate dehydrogenase 485573:486538 reverse MW:34162 | K22025 | 368 | 463.3 | 3.00E-141 | D-erythronate 2-dehydrogenase [EC:1.1.1.410] | K22025 | 368 | 464.5 | 1.40E-141 | D-erythronate 2-dehydrogenase [EC:1.1.1.410] | NA | NA | NA | NA | NA | NA | NA | NA | NA | NA | NA |
| LBHGKGNI_05200 | LHADMLFC_00436 | Glucitol operon repressor | Yes | Yes | srlR_6 Glucitol operon repressor 5489095:5489862 reverse MW:28811 | srlR_1 Glucitol operon repressor 486569:487336 reverse MW:28796 | . | . | . | . | . | . | . | . | . | . | NA | NA | NA | NA | NA | NA | NA | NA | NA | NA | NA |
| LBHGKGNI_05352 | LHADMLFC_02367 | Mercuric reductase | . | . | NA | NA | . | . | . | . | . | K00520 | 459.57 | 833.8 | 1.20E-252 | mercuric reductase [EC:1.16.1.1] | NA | NA | NA | NA | NA | NA | NA | NA | NA | NA | NA |
| LBHGKGNI_05353 | LHADMLFC_02368 | Mercuric transport protein MerC | . | . | NA | NA | . | . | . | . | . | K19058 | 39.77 | 92.2 | 3.00E-28 | mercuric ion transport protein | NA | NA | NA | NA | NA | NA | NA | NA | NA | NA | NA |
| LBHGKGNI_05354 | LHADMLFC_02369 | Mercuric transport protein periplasmic component | . | . | NA | NA | . | . | . | . | . | K08364 | 83.07 | 111.2 | 5.10E-34 | periplasmic mercuric ion binding protein | NA | NA | NA | NA | NA | NA | NA | NA | NA | NA | NA |
| LBHGKGNI_05355 | LHADMLFC_02370 | Mercuric transport protein MerT | . | . | NA | NA | . | . | . | . | . | K08363 | 52.83 | 148.5 | 1.40E-45 | mercuric ion transport protein | NA | NA | NA | NA | NA | NA | NA | NA | NA | NA | NA |
| LBHGKGNI_05356 | LHADMLFC_02371 | Mercuric resistance operon regulatory protein | . | . | NA | NA | . | . | . | . | . | K08365 | 154.5 | 212.8 | 2.10E-65 | MerR family transcriptional regulator, mercuric resistance operon regulatory protein | NA | NA | NA | NA | NA | NA | NA | NA | NA | NA | NA |
| LBHGKGNI_05375 | LHADMLFC_04212 | S-(hydroxymethyl)glutathione dehydrogenase | . | Yes | NA | frmA_2 S-(hydroxymethyl)glutathione dehydrogenase 4475459:4476568 forward MW:39273 | . | . | . | . | . | K00121 | 622.43 | 683.8 | 1.80E-207 | S-(hydroxymethyl)glutathione dehydrogenase / alcohol dehydrogenase [EC:1.1.1.284 1.1.1.1] | NA | NA | NA | NA | NA | NA | NA | NA | NA | NA | NA |
| LBHGKGNI_05379 | LHADMLFC_02359 | Dihydropteroate synthase | . | Yes | NA | folP_2 Dihydropteroate synthase 2479919:2480758 forward MW:30126 | . | . | . | . | . | K18974 | 472.53 | 488.4 | 5.10E-149 | dihydropteroate synthase type 1 [EC:2.5.1.15] | NA | NA | NA | NA | NA | NA | NA | NA | NA | NA | NA |
| LBHGKGNI_05493 | LHADMLFC_02355 | Tyrosine recombinase XerC | . | . | NA | NA | . | . | . | . | . | . | . | . | . | . | NA | NA | NA | NA | NA | NA | NA | NA | NA | NA | NA |
| LBHGKGNI_05600 | LHADMLFC_02353 | Tn3 family transposase TnAs2 | . | . | NA | NA | . | . | . | . | . | . | . | . | . | . | NA | NA | NA | NA | NA | NA | NA | NA | NA | NA | NA |
| LBHGKGNI_05693 | LHADMLFC_03095 | Copper-sensing transcriptional repressor CsoR | . | Yes | NA | csoR Copper-sensing transcriptional repressor CsoR 3280530:3280799 forward MW:10096 | . | . | . | . | . | K07807 | 90.2 | 141.1 | 7.60E-44 | uncharacterized protein | NA | NA | NA | NA | NA | NA | NA | NA | NA | NA | NA |
| LBHGKGNI_05695 | LHADMLFC_03097 | Protein YgiW | . | Yes | NA | ygiW_1 Protein YgiW 3282126:3282554 forward MW:15943 | . | . | . | . | . | . | . | . | . | . | NA | NA | NA | NA | NA | NA | NA | NA | NA | NA | NA |
